# Combinatorial treatment with traditional medicinal preparations and VEGFR-tyrosine kinase inhibitors for middle-advanced primary liver cancer: A systematic review and meta-analysis

**DOI:** 10.1371/journal.pone.0313443

**Published:** 2024-11-22

**Authors:** Hui-Bo Yu, Jia-Qi Hu, Bao-Jin Han, Yan-Yuan Du, Shun-Tai Chen, Xin Chen, Hong-Tai Xiong, Jin Gao, Hong-Gang Zheng

**Affiliations:** 1 Department of Oncology, Guang’anmen Hospital, China Academy of Chinese Medical Sciences, Beijing, China; 2 Graduate College, Beijing University of Chinese Medicine, Beijing, China; Al-Azhar University Faculty of Pharmacy for Boys, EGYPT

## Abstract

**Background:**

This study aimed to investigate the therapeutic efficacy and safety of Traditional medicine preparations (TMPs) given in combination with vascular endothelial growth factor receptor (VEGFR)-associated multi-targeted tyrosine kinase inhibitors (TKIs) for the treatment of middle to advanced-stage primary liver cancer (PLC).

**Methods:**

This systematic literature survey employed 10 electronic databases and 2 clinical trial registration platforms to identify relevant studies on the use of TMPs + VEGFR-TKIs to treat patients with middle-advanced PLC. Furthermore, a meta-analysis was performed following the PRISMA guidelines using the risk ratio (RR) at 95% confidence intervals (CI) or standardized mean difference as effect measures.

**Results:**

A total of 26 studies comprising 1678 middle-advanced PLC patients were selected. The meta-analysis revealed that compared with VEGFR-TKI mono-treatment, the co-therapy of TMPs + VEGFR-TKIs considerably enhanced the objective response rate (RR = 1.49, 95% CI: 1.31–1.69), disease control rate (RR = 1.23, 95% CI: 1.16–1.30), and one-year overall survival (RR = 1.49, 95% CI: 1.28–1.74). Furthermore, the co-therapy was associated with reduced incidences of liver dysfunction (RR = 0.64, 95% CI: 0.45–0.91), proteinuria (RR = 0.43, 95% CI: 0.24–0.75), hypertension (RR = 0.66, 95% CI: 0.53–0.83), hand-foot skin reactions (RR = 0.63, 95% CI: 0.49–0.80), myelosuppression (RR = 0.63, 95% CI: 0.46–0.87), and gastrointestinal reactions (RR = 0.64, 95% CI: 0.45–0.92). Moreover, the co-therapy indicated no increase in the incidences of rash and fatigue.

**Conclusion:**

This systematic analysis revealed that co-therapy with TMPs + VEGFR-TKIs has a higher effectiveness and safety profile for treating middle-advanced PLC patients. However, further validation using randomized control trials is required.

**PROSPERO registration no:**

CRD42022350634.

## Introduction

Malignant transformation of hepatocytes and bile duct cells leads to the development of primary liver cancer (PLC). It is among the most prevalent primary tumor manifestations. In 2020, PLC was ranked 6^th^ most prevalent malignancy and the 3^rd^ highest cause of death globally, accounting for about 910,000 novel PLC patients and 830,000 PLC-related deaths [1]. Currently, surgery is the most effective method for achieving long-term survival in PLC patients. However, because of the lack of apparent symptoms at early stages, PLC onset is relatively insidious, and its progression is rapid. Therefore, most PLC patients are diagnosed at intermediate or advanced stages when the best opportunity for radical treatment has been lost.

The Vascular Endothelial Growth Factor (VEGF) family comprises VEGF-A, VEGF-B, VEGF-C, VEGF-D, and placental growth factor [2]. These proteins predominantly bind VEGF receptor-1 (VEGFR-1) or Fms-like Tyrosine Kinase-1, and VEGFR-2 (also called kinase insert domain-containing receptor) [2, 3]. This interaction activates downstream signaling pathways crucial for endothelial cell proliferation, differentiation, and migration, as well as the regulation of vascular permeability, which are essential for angiogenesis. Angiogenesis ensures a steady supply of oxygen and nutrients during tumor development and progression. VEGF, secreted by tumor cells and their microenvironment, binds to VEGFR-2 and exerts a pivotal role in vascular permeability and neo-angiogenesis [4]. In 1993, a monoclonal antibody targeting and neutralizing VEGFA was identified to inhibit tumor growth in xenograft models, which opened translational avenues for targeting VEGF-VEGFR signaling [5]. These therapeutic agents can be broadly categorized into those which target the VEGF ligand and those which inhibit the cell surface receptor [6]. VEGFR-TKIs is a class of small molecules targeted therapies that can selectively inhibit the phosphorylation of tyrosine kinase receptors, thereby suppressing tumor angiogenesis. Recently, the National Comprehensive Cancer Network guidelines have listed several VEGFR-TKIs (e.g., sorafenib, lenvatinib) as the first-line treatment for middle-advanced PLC based on their survival benefits observed in different countries and regions [7–12]. Furthermore, alternative VEGFR-TKIs such as regorafenib and apatinib are also being used [13]. Although the advent of targeted therapy for PLC treatment marks a significant epoch, some randomized controlled trials indicated that sorafenib-treated patients had a median overall survival (OS) of only 10.7 months [10], while that of lenvatinib-treated patients was 13.6 months [9]. Moreover, several adverse drug reactions (ADRs) have also been associated with oral targeted drugs, including hypertension, myelosuppression, neurotoxicity, gastrointestinal reactions, and drug resistance, which severely influence the treatment outcome for PLC [14].

In recent years, in addition to targeted therapies, significant breakthroughs in immunotherapy have also been made. A meta-analysis revealed that in patients undergoing immunotherapy (either monotherapy or in combination with other anticancer agents), the pooled odds ratio was 1.67 [95% confidence interval (CI): 1.52–1.84]. Compared to control treatments, immune checkpoint inhibitors have indicated substantially increased rates of achieving complete response [15]. Moreover, gender was observed to influence the efficacy of immune checkpoint inhibitors in cancer patients, with males generally experiencing greater overall benefit after this therapy [16, 17]. It has been indicated that patients with advanced hepatocellular carcinoma (HCC) can benefit from immunotherapy [18]. Furthermore, for treating HCC, many studies have revealed the efficacy of immunotherapy, such as Tremelimumab + Durvalumab and Durvalumab alone [19], as well as the combination of immunotherapy with targeted therapy, such as Atezolizumab + Bevacizumab [20]. The National Comprehensive Cancer Network (2024) guidelines have also approved these treatment strategies.

Despite advancements in treatment options, PLC remains a significant burden for individuals and society. Therefore, alternative therapeutic modalities are urgently required for prolonged survival, enhanced quality of life, and reduced ADRs in PLC cases. New complementary treatment combined with targeted therapy is a possible avenue for exploration. Tradition medicinal preparations (TMPs) comprise traditional prescriptions, extracts, injections, proprietary Chinese medicines, and clinician-prepared decoctions. Several clinical trials have indicated that combining TMPs and loco-regional or systemic therapies increases PLC treatment efficacy [21–23], reduces ADRs [24], and improves patient’s quality of life [25], highlighting its potential as a promising approach. Furthermore, mechanistic studies have revealed that nobiletin, a *Citrus aurantium* L. (Rutaceae) extract, inhibits proliferation, promotes apoptosis [26], reverses multidrug resistance [27], and inhibits metastasis [28] in HCC. Moreover, in HCC, ginsenoside Rg3 and Huaier granule have been indicated to inhibit tumor proliferation [29, 30], reduce tumor cell viability [31], decrease tumor metastasis, prolong survival in mouse models [32], and improve the efficacy of the chemotherapeutic drug [33]. Some traditional medicines and their extracts such as the compound Kushen injection [34], artesunate [35], emodin [36], and catalpol [37] can sensitize HCC cells to the anticancer activity of sorafenib, possibly by combating targeted drug resistance and increasing the effect of tyrosine kinase inhibitors (TKIs).

Much literature indicated that VEGFR-TKIs have enhanced the efficacy and reduced adverse drug reactions in PLC patients treated with combined TMPs + VEGFR-TKIs therapy compared to VEGFR-TKIs alone. These findings suggest that compared to current therapeutic modalities, TMPs may serve as effective complementary or alternative treatments for PLC with more favorable risk-benefit profiles. However, because of the limited number of clinical trials investigating the co-treatment of TMPs + VEGFR-TKIs and their small sample sizes, the evidence for its potential use is less convincing. Therefore, our objective is to assess the therapeutic efficacy and safety of TMPs in combination with VEGFR-TKIs for middle-to-advanced PLC, aiming to provide substantial evidence.

## Methodology

### Experimental designs

This study performed a systematic review and meta-analysis based on the guideline of Preferred Reporting Items for Systematic Reviews and Meta-Analysis (PRISMA; S1 File). The protocol has been registered in PROSPERO (CRD42022350634).

### Eligibility criteria

#### Investigation profiles

Randomized controlled trials in English or Chinese languages were selected for this study.

#### Patients

This study included patients diagnosed with middle-advanced PLC according to pathological, cytological, and/or imaging diagnosis criteria. There were no restrictions on patient sex or age.

#### Interventions

There were two cohorts in this study; the experimental cohort received TMPs combined with VEGFR-TKIs, while the control cohort was administered with the same VEGFR-TKIs regimen without TMPs. All formulations or administrations of TMPs, such as decoction, pill, granule, and injection were included. Furthermore, all studies with a minimum treatment course of four weeks were included.

#### Primary outcomes

The primary outcome for treating tumor progression was measured through objective response rate (ORR): complete response (CR) and partial response (PR), together with disease control rate (DCR): CR + PR + stable disease (SD). Outcomes were evaluated before the trial started and after the follow-up ended. For assessment criteria, the guidelines of Response Evaluation Criteria in Solid Tumors (RECIST) [38] and the World Health Organization (WHO) [39] were followed.

#### Secondary outcomes

The secondary outcomes included one-year OS, quality-of-life, level of alpha-fetoprotein (AFP), and ADRs. The Karnofsky Performance Status (KPS) was employed to assess the quality of life. Furthermore, ADR incidences were evaluated for liver dysfunction, proteinuria, hypertension, hand-foot skin reactions, gastrointestinal reactions, myelosuppression, fatigue, and rash. Secondary outcomes were also evaluated before the trial started and after the follow-up ended.

### Exclusion criteria

Studies with (1) incomplete data that could not be used for further analyses, (2) duplicated data, (3) lack of a suitable control cohort, (4) uncertain tumor stage, and (5) not consistent TMPs use in a trial were excluded.

### Literature survey

All randomized controlled trials, published in both Chinese and English, were searched from January 1, 2000 until April 12, 2024. The scientific investigation repositories employed included PubMed, EMBASE, Cochrane Central Register of Controlled Trials (CENTRAL), Turning Research into Practice (TRIP), Latin American and Caribbean Health Sciences Literature (LILACS), Alt HealthWatch, China National Knowledge Infrastructure (CNKI), Chinese Biomedical Literature Database (CBM), Wangfang Datasets Knowledge Service Platform, Chinese Scientific Journal Database (VIP database), clinicaltrials.gov, and Chinese Clinical Trial Registry.

Search terms employed for English databases included: “cancer*”, “carcinoma”, “neoplasms”, “hepatocellular”, “traditional medicine”, “complementary therapies”, “Chinese herbal”, “herbal medicine”, “Sorafenib”, “target therapy”, “Vascular Endothelial Growth Factor”, “lenvatinib”, “apatinib”, “regorafenib”, together with “random”. For Chinese repositories, synonymous Chinese terms were utilized (S2 File indicates full search strategy). References from related studies were also reviewed to retrieve additional studies. This investigation was conducted by two assessors and any dispute was settled by a third reviewer [40].

### Published studies screening and dataset extraction

Study eligibility was independently assessed by two assessors and any disputes were settled by consensus conversation with a third assessor. The following information was extracted: 1. General [title, author, year(s)]; 2. Methodology (study design, baseline comparability, randomization and blinding, loss of follow-up, and selective reporting); 3. Patients (diagnostic criteria, sample size, age, sex, stage of PLC); 4. Intervention (treatment regimens, composition of TMPs, drug dosage, delivery route, therapeutic timeframe); and 5. Investigation endpoints.

### Methodology quality evaluation

The selected studies were independently evaluated by two reviewers and any disagreement was resolved by a third reviewer. The quality of these studies was assessed using the Cochrane risk-of-bias tool covering seven domains: random sequence generation, allocation concealment, blinding of participants and personnel, blinding of outcome assessment, incomplete outcome data, selective reporting, and other biases. Each domain was scored for low, unclear, or high risk of bias [40].

### Datasets analyses

Meta-analysis was performed by two reviewers independently using the RevMan5.4 and Stata17 software. Using the risk ratio (RR), which represents the ratio of the studied outcomes between the co-treatment group and the mono treatment group, along with 95% confidence intervals (CI), dichotomous variables were identified. For continuous variables, the standardized mean difference (SMD) with 95% CI was employed.

The heterogeneity of the selected trials was evaluated and those with *I*^*2*^ < 75% (defining no significant statistical heterogeneity) were included for meta-analysis. Furthermore, data from trials with *I*^*2*^ ≤ 25% were pooled with a fixed-effect model (FEM). Trials with 25% < *I*^*2*^ < 75% were assessed for heterogeneity sources *via* the sensitive and sub-cohort analyses. Studies that achieved *I*^*2*^ ≤ 25% were evaluated through FEM, while those that did not were analyzed through a random-effects model (REM) within meta-analyses. Moreover, data from significantly heterogeneous trials (*I*^*2*^ ≥ 75%) were not pooled because such large heterogeneity cannot be explained *via* sub-cohort analyses. To eliminate the possible publication bias, funnel-plot, Begg’s test, and Egger’s test were performed for meta-analysis with ≥ 10 trials.

Subgroup analyses were conducted in accordance with the target drug regimen, course of treatment, KPS score, and form of TMPs (e.g., decoction, granule, pill, and powder). Sensitivity assessment indicated whether primary analyses of trials with and without high risks are sufficiently robust, and if the FEM or REM-based meta-analyses are equally reliable.

### Proof quality evaluation

Proof quality from study endpoints was independently evaluated by two reviewers based on the GRADE (Grading of Recommendations Assessment Development and Evaluation) criteria [41], which included 5 domains: risk of bias, inconsistency of trials, indirectness of evidence, imprecision of results, and publication bias. For each domain, four levels of scoring (high, moderate, low, and very low) were applied. Any dispute was settled through a third evaluator.

### Sensitivity analyses of the administered TMPs interventions

To explore the effect of mono or co-treatment of traditional medicines on the efficacy of VEGFR-TKIs in PLC patients, sensitivity analyses of multi-component TMPs were performed based on the composition of TMPs. The rationale of this approach was provided by Chen *et al*. that the anti-tumor properties of a particular traditional medicine can be reflected in pooled results of the studies on this medicine [42]. In this research, some multi-component TMPs and their several formulations or combinations were studied. Thus, the increased efficacy of medicine by the addition of TMPs with common traditional medicines can be explored by the pooled RRs in study cohorts. Moreover, it may identify specific traditional medicine combinations that contribute the most to the treatment of PLC.

This approach involved producing a matrix of pooled RRs from multiple sub-cohort studies *via* a multilevel procedure. Level 1) all studies using a single identical traditional medicine were regarded as a sub-cohort and the pooled RR and *I*^*2*^ for ORR and DCR were determined. Dataset outcomes were compiled in descending order and any considerable dataset outcomes were recorded. Traditional medicines from sub-cohorts with no major influence on ORR/DCR and major heterogeneity (*I*^2^ > 30%) were excluded from the study. Level 2, the same pair of traditional medicines within TMP interventions were identified as sub-cohorts, and the RRs were calculated. Level 3, combinations of three medicinal plants were identified until no eligible combinations were available. Traditional medicines that met the following inclusion criteria [42] were selected for further analysis:

1. Higher sub-cohort RRs for experimental *vs*. control.
2. Higher sub-cohort RRs than those within the total pool.
3. No significant heterogeneity (*I*^*2*^ ≤ 30%).
4. The sub-cohort RRs were significant at multilevel combinations.

## Results

### Literature search

**Fig 1:**
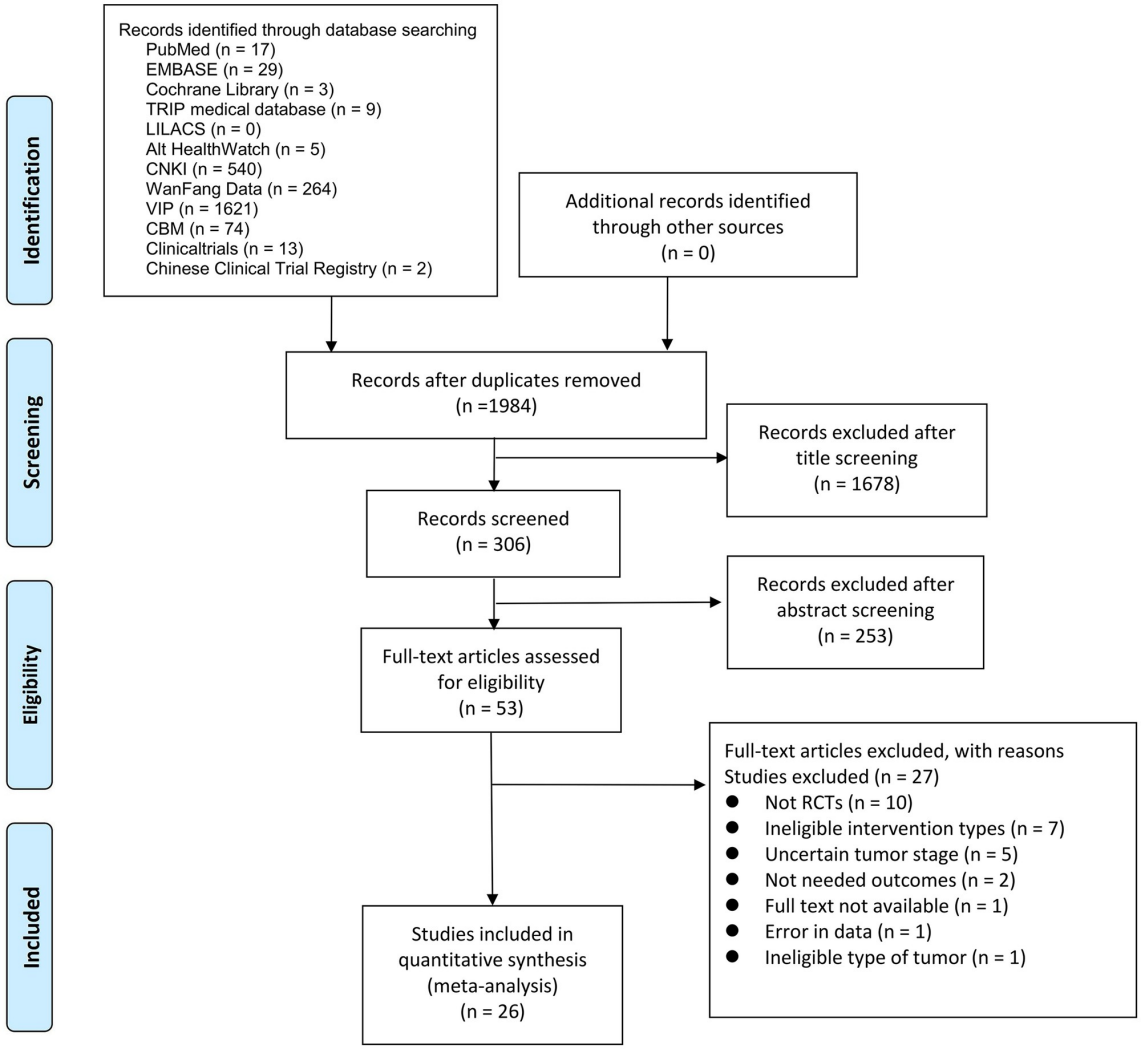
Flow chart illustrating the literature survey strategy.

A total of 2577 potential studies were identified (Fig 1). After removing the duplicated articles, 1984 investigations remained (S3 File). Title screening excluded 1678 articles and 306 remained after abstract screening. Finally, after the full-text screening, 53 articles remained, of which 26 met the inclusion criteria, and therefore were selected for further analyses. Dataset collection and quantitative synthesis were performed for the 26 eligible studies.

### Profiling of analyzed studies

**Fig 2:**
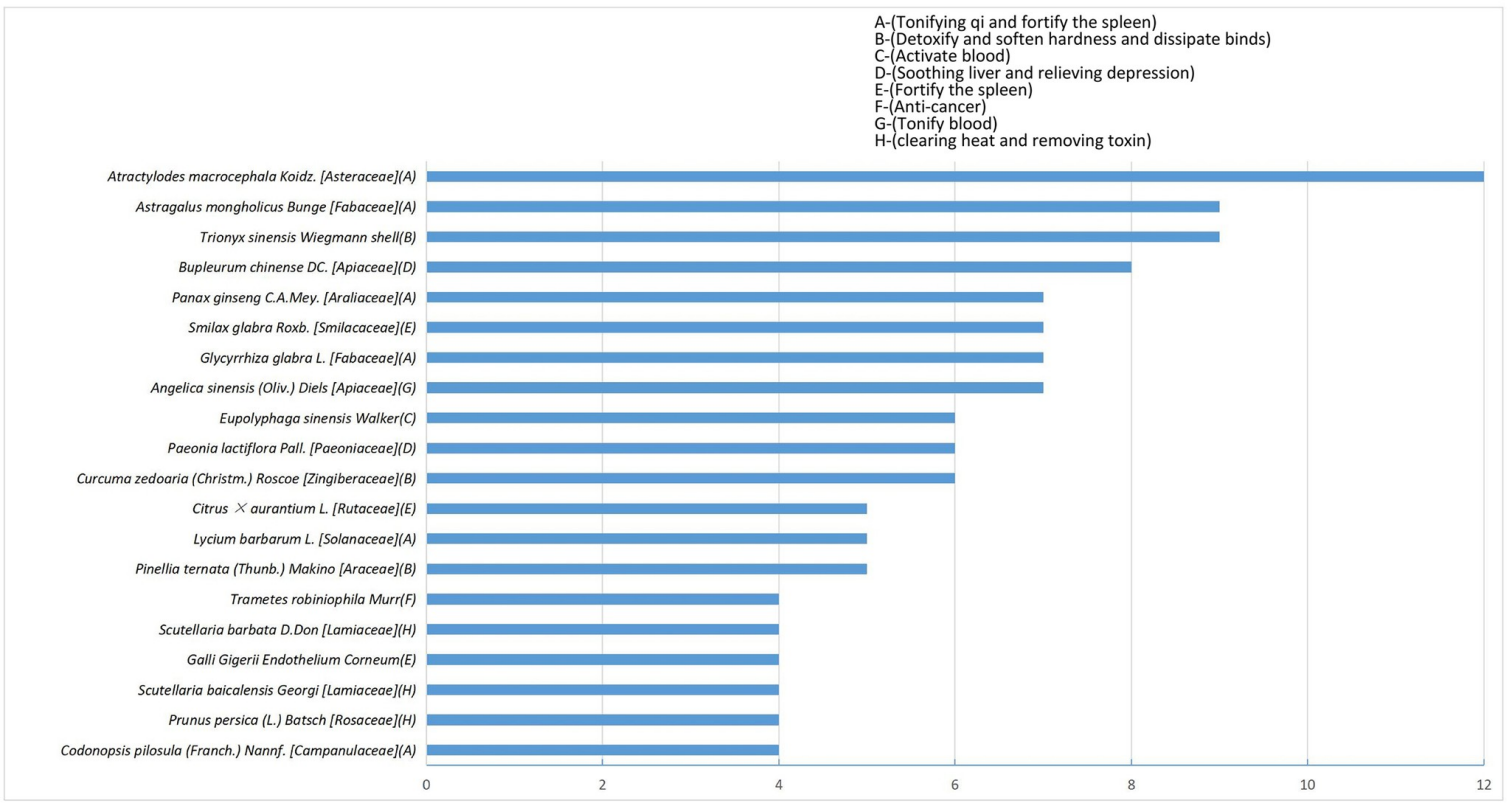
Frequency and functional classification of the top 20 traditional medicines.

This investigation included 26 randomized controlled trials [43–68] comprising 1678 patients (841 experimental and 837 control). Table 1 reflects the basic profiles of the selected investigations. The sample size of the studies was between 38–119. In the TMP cohort, 15 studies [43, 44, 46–50, 52, 54, 55, 57, 59, 61, 63, 66] used decoction, 6 utilized granules [53, 58, 62, 64, 65, 68], 2 used pill [45, 51], 1 employed powder [67], 1 used bolus [60], and only 1 study utilized injection [56]. All studies used the oral route of delivery, except for one study [56] which used intravenous administration. The general principles of TMPs included heat dissipation toxin removal, tonifying, and blood activation, liver soothing, depression relief, qi tonifying, and spleen fortifying. In total, 24 TMPs were included, involving 105 types of traditional medicines. Table 2 presents the specific formulations, medicines, dosages, and drug quality control. The frequency of use of the top 20 traditional medicines is provided in Fig 2. For VEGFR-TKI regimens, 15 studies [43–45, 47, 51, 52, 55, 56, 58, 60, 62, 63, 65–67] used sorafenib, 6 used apatinib [46, 49, 54, 57, 61, 64], 4 used lenvatinib [48, 50, 59, 68], and 1 used regorafenib [53]. All VEGFR-TKIs were administered orally. Moreover, 21 studies [43–46, 50, 52–65, 67, 68] reported ORR and DCR based on the RECIST or WHO guidelines. Eight studies [43, 44, 47, 58, 60, 65–67] reported one-year OS, ten [43, 47, 48, 52, 58, 59, 61, 62, 64, 68] reported quality of life according to the Karnofsky Performance Status (KPS), fourteen [44, 46, 48, 50, 52–54, 58, 60–62, 64, 67, 68] indicated the AFP levels, and 20 studies [44–46, 48–54, 56, 58, 60–62, 64–68] revealed ADRs.

**Table 1:** Characteristics of studies on middle-advanced PLC with TMPs and VEGFR-TKIs.

Study ID	Simple Size (T/C)	M/F	QoL score	Stage	Age(T/C), median or mean±SD	Interventions			Course of Treatment	Outcomes
						TMPs	Drug delivery	Targeted drugs Regimen		
DuanKN 2018	23/22	T:17/6 C:15/7	/	III, IV	T(65.66±2.12) C(65.70±2.23)	Self prescribed Decoction, qd	Orally	Sorafenib,400mg, bid	2m	123
FangHS 2015	30/30	T:17/13 C:16/14	KPS>60	III:7 IVa:6 IVb:47	T(59.50±11.23) C(57.90±10.59)	Shentao Ruangan Decoction,150-200mL, qd	Orally	Sorafenib,400mg, bid	1m	1245
FengLH 2012	30/29	T:25/5 C:25/4	KPS>60	II, III, IV	T:56.4 C:55.8	Hua Chan Su Pills:1.2g, tid	Orally	Sorafenib,400mg, bid	2m	15
GaoYY 2022	47/47	T:29/18 C:31/16	/	IV:94	T(62.17±6.03) C(61.51±6.28)	Peiyuan Kangai Decoction, 200mL, bid	Orally	Apatinib,500mg, qd	2m	145
JinZ 2022	29/29	T:18/12 C:20/10	KPS≥30	IIb, IIIa, IIIb	T(56.7±4.6) C(56.4±4.3)	Huisheng oral liquid, 10mL, tid	Orally	Lenvatinib, >60kg~12mg, qd <60kg~8mg, qd	2m	145
HanGM 2021	29/29	T:10/19 C:13/16	/	III, IV	T(53.36±10.25) C(53.73±10.41)	Jiawei Chaihu Biejia Decoction, bid	Orally	Sorafenib,400mg, bid	2m	23
JiangXQ 2022	37/38	T:20/17 C:22/16	KPS≥30	III:21 IV:54	T(61.21±4.28) C(61.28±4.30)	Chaihu Shugan Huayu Decoction, 200mL, bid	Orally	Lenvatinib, >60kg~12mg, qd <60kg~8mg, qd d1-d21,21d/C	3cycles	345
JinJ 2020	29/28	T:23/6 C:23/5	KPS≥60	IIIa:15 IIIb:18 IV:24	unclear	Ganji Decoction, bid	Orally	Apatinib, 250mg, qd,d1-d28,28d/C	2C	5
LiDQ 2018	30/30	36/24	ECOG 0–2	III:20 IV:40	(51.12±10.01)	Wu Zhi Pills: 0.93g, tid	Orally	Sorafenib,400mg, bid	1m	5
LiuJP 2018	33/33	T:16/17 C:18/15	KPS>60	III, IV	T(56.6±5.5) C(56.9±5.5)	Qinghuo Tongluo Decoction, 200mL, tid	Orally	Sorafenib,400mg, bid	3m	1345
MaJR 2022	55/55	T:30/25 C:32/23	/	IIb:30 IIIa:67 IIIb:13	T(54.96±9.15) C(55.31±9.20)	Huazhi Rougan Granule, 8g, tid	Orally	Regorafenib,160mg, qd, d1-d21,28d/C	3cycles	145
MaYK 2018	20/20	T:17/3 C:15/5	KPS≥70	BCLC: C:33 D:7	T(58.8±7.7) C(59.6±9.3)	Jianpi Jiedu Decoction, 150mL, bid	Orally	Apatinib,250mg, qd	2m	145
QiaoCX 2015	20/20	T:14/6 C:19/1	/	II, III, IV	T:median:49 C:median:46.5	Fuzheng Guben Decoction, 100mL, bid	Orally	Sorafenib,400mg, bid	2m	1
SunJ 2022	60/59	T:32/28 C:34/25	KPS T(79.63±10.12) C(80.48±10.23)	III:62 IV:57	T(53.96±4.58) C(54.15±4.63)	Elemene Injection, 0.6g, qd	Intravenously	Sorafenib,400mg, bid	6m	15
SunY 2019	30/30	T:17/13 C:16/14	KPS>60	II:15 III:21 IV:24	T(65.69±1.38) C(65.53±1.25)	Jiawei Yiguan Decoction, 120mL, bid	Orally	Apatinib,500mg, qd	>1m	1
TangYF 2018	57/56	T:40/17 C:41/15	/	III, IV	T(51.97±7.41) C(52.34±7.27)	Huai Er Granule: 20g, tid	Orally	Sorafenib,400mg, bid	2m	12345
TuXL 2021	30/30	T:19/11 C:18/12	/	BCLC: C:60	T(53.93±5.29) C(53.56±6.12)	Jianpi Yanggan Jiedu Decoction, 150mL, bid	Orally	Lenvatinib, >60kg~12mg, qd <60kg~8mg, qd	2m	13
WangGT 2016	18/20	24/14	/	IVa:36 IVb:2	(45.2±4.6)	Songxiang Bolus,20g, bid	Orally	Sorafenib,400mg, bid	Average duration of medication 157.4d	1245
WuYW 2018	30/30	T:27/3 C:28/2	KPS≥60	III:16 IV:44	T(58.04±11.41) C(60.8±14.11)	Jianpi Rougan Decoction,125mL, bid	Orally	Apatinib,250mg, qd, d1-d28,28d/C	>1cycles	1345
YangCJ 2021	27/26	T:19/8 C:20/6	KPS≥60	BCLC: B:18 C:35	T(56.96±9.89) C(54.88±10.25)	Jiawei Xiao Chaihu Granule, tid	Orally	Sorafenib,400mg, bid	2m	1345
YuJF 2021	33/32	T:27/6 C:27/5	ECOG 0–2	BCLC: C:65	T(47.33±12.35) C(48.82±12.34)	Fuling Sini Decoction	Orally	Sorafenib,400mg, bid	>1m	1
Zhang QH 2019	30/30	T:23/7 C:21/9	KPS>60	III, IV	T(49.63±9.06) C(52.57±8.92)	Huai Er Granule: 20g, tid	Orally	Sorafenib,400mg, bid	2m	125
ZhangZ 2019	28/28	T:18/10 C:20/8	/	BCLC: B, C	T(55.93±7.03) C(56.68±6.56)	Yiqi Huayu Jiedu Decoction, bid	Orally	Sorafenib,400mg, bid	Until the patient had grade 3 or 4 adverse reactions or died	25
ZhanLH 2022	30/30	T:18/12 C:21/9	KPS≥60	T:IIb:15, IIIa:15 C:IIb:13,IIIa:17	T(58.83±10.93) C(58.80±12.93)	Chai Shao Granule, bid	Orally	Apatinib,750mg, qd	3m	1345
ZhouFJ 2020	30/30	T:17/13 C:18/12	KPS≥60	III, IV	T(59.51±11.22) C(59.80±10.58)	Jiajian Sanjia Powder, 10g, tid	Orally	Sorafenib,400mg, bid, d1-d28,28d/C	>1cycles	1245
ZhuX 2023	26/26	T:21/5 C:22/4	KPS≥60	III:40 IV:12	T(60.00±9.45) C(61.38±9.19)	Danzhi Xiaoyao Granule, 300ml, bid	Orally	Lenvatinib, >60kg~12mg, qd <60kg~8mg, qd	2m	135

**Table 2:** The formulation, medicines and dosage, and control of drug quality.

Study	Formulation	Source	Species, concentration	Quality control reported? (Y/N)	Chemical analysis reported? (Y/N)
DuanKN 2018	Self prescribed Decoction	Shanxi Taiyuan Hospital of Traditional Chinese Medicine	*Dioscorea oppositifolia* L. [Dioscoreaceae] 30g, *Akebia trifoliata* (Thunb.) Koidz. [Lardizabalaceae] 30g, *Astragalus mongholicus* Bunge [Fabaceae] 20-30g, Galli Gigerii Endothelium Corneum 20g, *Rehmannia glutinosa* (Gaertn.) DC. [Orobanchaceae] 15-20g, *Areca catechu* L. [Arecaceae] 15g, *Solanum nigrum* L. [Solanaceae] 15g, *Scutellaria barbata* D.Don [Lamiaceae] 15g, *Paeonia lactiflora* Pall. [Paeoniaceae] 15g, *Atractylodes macrocephala* Koidz. [Asteraceae] 10g, *Eupolyphaga sinensis* Walker 5g, *Scolopendra subspinipes mutilans* 2.	Y—Quality controlled by Shanxi Taiyuan Hospital of Traditional Chinese Medicine	N
FangHS 2015	Shentao Ruangan Decoction	The first affiliated hospital of Guangzhou university of Chinese medicine	*Panax ginseng* C.A. Mey.15g, *Prunus persica* (L.) Batsch [Rosaceae]10g, *Angelica sinensis* (Oliv.) Diels [Apiaceae]10g, *Agrimonia pilosa* var. nepalensis (D.Don) Nakai [Rosaceae]30g, *Cremastra appendiculata* (D.Don) Makino [Orchidaceae]15g, *Scutellaria barbata* D.Don [Lamiaceae]15g, *Atractylodes macrocephala* Koidz. [Asteraceae]15g, *Glycyrrhiza glabra* L. [Fabaceae]6g.	Y—Quality controlled by the first affiliated hospital of Guangzhou university of Chinese medicine	N
FengLH 2012	Hua Chan Su Pills	China resources Jinchan Pharmaceuticals Co. Ltd	Bufonis Corium.	Y- Prepared according to The Pharmacopoeia of the People’s Republic of China	Y- TLC
GaoYY 2022	Peiyuan Kangai Decoction	Characteristic Medical Center of the Chinese Armed Police Force	*Astragalus mongholicus* Bunge [Fabaceae] 30g, *Atractylodes macrocephala* Koidz. [Asteraceae]15g, *Ligustrum lucidum* W.T.Aiton [Oleaceae]15g, *Scrophularia ningpoensis* Hemsl. [Scrophulariaceae] 15g, *Eucommia ulmoides* Oliv. [Eucommiaceae]15g, *Hordeum vulgare* L. [Poaceae]15g, *Cullen corylifolium* (L.) Medik. [Fabaceae]15g, *Pinellia ternata* (Thunb.) Makino [Araceae]15g, *Citrus × aurantium* L.10g, *Wurfbainia villosa* (Lour.) Skornick. & A.D.Poulsen [Zingiberaceae]10g, *Curcuma aromatica* Salisb. [Zingiberaceae]10g, *Eupolyphaga sinensis* Walker 10g, *Gypsophila vaccaria* (L.) Sm. [Caryophyllaceae] 12g.	Y—Quality controlled by Characteristic Medical Center of the Chinese Armed Police Force	N
JinZ 2022	Huisheng oral liquid	Chengdu Di’ao Group Tianfu Pharmaceutical Stock Co.,Ltd.	*Panax ginseng* C.A.Mey., *Angelica sinensis* (Oliv.) Diels [Apiaceae], *Paeonia lactiflora* Pall. [Paeoniaceae], *Curcuma longa* L. [Zingiberaceae], *Tetradium ruticarpum* (A.Juss.) T.G.Hartley [Rutaceae], *Alisma plantago-aquatica* subsp. orientale (Sam.) Sam. [Alismataceae], *Rheum palmatum* L. [Polygonaceae], *Cyperus rotundus* L. [Cyperaceae], *Conioselinum anthriscoides ’Chuanxiong’* [Apiaceae], *Sparganium stoloniferum* (Buch.-Ham. ex Graebn.) Buch.-Ham. ex Juz. [Typhaceae], *Prunus persica* (L.) Batsch [Rosaceae], *Boswellia sacra* Flück. [Burseraceae], *Commiphora myrrha* (T.Nees) Engl. [Burseraceae], *Trionyx sinensis Wiegmann shell*, *Syzygium aromaticum* (L.) Merr. & L.M.Perry [Myrtaceae], *Corydalis yanhusuo* (Y.H.Chou & Chun C.Hsu) W.T.Wang ex Z.Y.Su & C.Y.Wu [Papaveraceae], *Rehmannia glutinosa* (Gaertn.) DC. [Orobanchaceae].	Y- Prepared according to The Pharmacopoeia of the People’s Republic of China	Y- TLC, HPLC
HanGM 2021	Jiawei Chaihu Biejia Decoction	Yuebei people’s Hospital	*Pseudostellaria heterophylla* (Miq.) Pax [Caryophyllaceae] 12g, *Panax japonicus* (T.Nees) C.A.Mey. [Araliaceae]12g, *Atractylodes macrocephala* Koidz. [Asteraceae]12g, *Smilax glabra* Roxb. [Smilacaceae]15g, *Crataegus monogyna* Jacq. [Rosaceae] 9g, Galli Gigerii Endothelium Corneum 15g, *Pinellia ternata* (Thunb.) Makino [Araceae] 9g, *Citrus × aurantium* L. 5g, *Bergenia purpurascens* (Hook.f. & Thomson) Engl. [Saxifragaceae]15g, *Strobilanthes cusia* (Nees) Kuntze [Acanthaceae] 15g, *Smilax glabra* Roxb. [Smilacaceae] 15g, *Hypericum japonicum* Thunb. [Hypericaceae] 15g, *Bursa bursa-pastoris rhomboidea* Shull. 15g, *Prunella vulgaris* L. [Lamiaceae] 9g, Crassostrea 15g, *Arisaema amurense* Maxim. [Araceae] 9g, *Curcuma aromatica* Salisb. [Zingiberaceae]9g, Gekko gecko 5g, *Ageratum conyzoides* L. [Asteraceae] 6g, *Astragalus mongholicus* Bunge [Fabaceae] 15g, *Angelica sinensis* (Oliv.) Diels [Apiaceae] 9g.	Y—Quality controlled by Yuebei people’s Hospital	N
JiangXQ 2022	Chaihu Shugan Huayu Decoction	Baoji People’s Hospital	*Salvia miltiorrhiza* Bunge [Lamiaceae] 20g, *Paeonia lactiflora* Pall. [Paeoniaceae] 20g, Trionyx sinensis Wiegmann shell 20g, Crassostrea 20g, *Bupleurum chinense* DC. [Apiaceae] 15g, *Scrophularia ningpoensis* Hemsl. [Scrophulariaceae] 15g, *Fritillaria thunbergii* Miq. [Liliaceae] 15g, *Conioselinum anthriscoides* ’Chuanxiong’ [Apiaceae] 15g, *Citrus × aurantium* L. [Rutaceae] 15 g, *Citrus × aurantium* L. [Rutaceae] 10g, *Cyperus rotundus* L. [Cyperaceae] 10g, *Santalum album* L. [Santalaceae] 6g, *Panax notoginseng* (Burkill) F.H.Chen [Araliaceae] 5g, *Glycyrrhiza glabra* L. [Fabaceae] 8g.	Y—Quality controlled by Baoji People’s Hospital	N
JinJ 2020	Ganji Decoction	Guangdong Integrated Traditional Chinese and Western Medicine Hospital	*Codonopsis pilosula* (Franch.) Nannf. [Campanulaceae]15g, *Astragalus mongholicus* Bunge [Fabaceae]15g, *Bupleurum chinense* DC. [Apiaceae]10g, *Paeonia lactiflora* Pall. [Paeoniaceae]15g, *Curcuma aromatica* Salisb. [Zingiberaceae]15g, *Atractylodes macrocephala* Koidz. [Asteraceae]15g, *Scrophularia ningpoensis* Hemsl. [Scrophulariaceae]10g, *Lycium barbarum* L. [Solanaceae]15g, Galli Gigerii Endothelium Corneum 15g, *Akebia trifoliata* (Thunb.) Koidz. [Lardizabalaceae]10g, Trionyx sinensis Wiegmann shell 15g, *Curcuma longa* L. [Zingiberaceae]15g.	Y—Quality controlled by Guangdong Integrated Traditional Chinese and Western Medicine Hospital	N
LiDQ 2018	Wu Zhi Pills	Guangxi Fanglue Pharmaceutical Group Co. Ltd.	Schisantherin A	Y- Prepared according to The Pharmacopoeia of the People’s Republic of China	Y- TLC
LiuJP 2018	Qinghuo Tongluo Decoction	Wuhan Central Hospital	*Scleromitrion diffusum* (Willd.) R.J.Wang [Rubiaceae], *Scutellaria barbata* D.Don [Lamiaceae], *Paris polyphylla* Sm. [Melanthiaceae], *Reynoutria japonica* Houtt. [Polygonaceae], *Swertia chirayita* (Roxb.) H.Karst. [Gentianaceae], *Manis pentadactyla* Linnaeus, *Scolopendra subspinipes mutilans*,Scorpion, *Eupolyphaga sinensis* Walker, *Tripidium arundinaceum* (Retz.) Welker, Voronts. & E.A.Kellogg [Poaceae].	Y—Quality controlled by Wuhan Central Hospital	N
MaJR 2022	Huazhi Rougan Granule	Shandong New Time Pharmaceutical Co. Ltd.	*Swertia chirayita* (Roxb.) H.Karst. [Gentianaceae], *Senna tora* (L.) Roxb. [Fabaceae], *Rheum palmatum* L. [Polygonaceae], *Crotalaria albida* B.Heyne ex Roth [Fabaceae], *Crataegus monogyna* Jacq. [Rosaceae], *Atractylodes macrocephala* Koidz. [Asteraceae], *Citrus × aurantium* L. [Rutaceae], *Glycyrrhiza glabra* L. [Fabaceae], *Lycium barbarum* L. [Solanaceae], *Astragalus mongholicus* Bunge [Fabaceae], *Glycyrrhiza glabra* L. [Fabaceae], *Paeonia lactiflora* Pall. [Paeoniaceae].	Y- Prepared according to The Pharmacopoeia of the People’s Republic of China	Y- TLC, HPLC
MaYK 2018	Jianpi Jiedu Decoction	Shanghai Xuhui District Central Hospital	*Pseudostellaria heterophylla (Miq*.*) Pax [Caryophyllaceae] 12g*, *Panax japonicus (T*.*Nees) C*.*A*.*Mey*. *[Araliaceae]12g*, *Atractylodes macrocephala Koidz*. *[Asteraceae]12g*, *Smilax glabra Roxb*. *[Smilacaceae]15g*, *Crataegus monogyna Jacq*. *[Rosaceae] 9g*, *Galli Gigerii Endothelium Corneum 15g*, *Pinellia ternata (Thunb*.*) Makino [Araceae] 9g*, *Citrus × aurantium L*. *5g*, *Bergenia purpurascens (Hook*.*f*. *& Thomson) Engl*. *[Saxifragaceae]15g*, *Strobilanthes cusia (Nees) Kuntze [Acanthaceae] 15g*, *Smilax glabra Roxb*. *[Smilacaceae] 15g*, *Hypericum japonicum Thunb*. *[Hypericaceae] 15g*, *Bursa bursa-pastoris (L*.*) Medik*. *[Brassicaceae] 15g*, *Prunella vulgaris L*. *[Lamiaceae]9g*, *Crassostrea 15g*, *Arisaema amurense Maxim*. *[Araceae] 9g*, *Curcuma aromatica Salisb*. *[Zingiberaceae]9g*, *Gekko gecko 5g*, *Ageratum conyzoides L*. *[Asteraceae] 6g*, *Astragalus mongholicus Bunge [Fabaceae] 15g*, *Angelica sinensis (Oliv*.*) Diels [Apiaceae] 9g*.	Y—Quality controlled by Shanghai Xuhui District Central Hospital	N
QiaoCX 2015	Fuzheng Guben Decoction	He’nan Institute of Traditional Chinese Medicine Research Hospital	*Panax ginseng C*.*A*.*Mey*., *Ganoderma lucidum*, *Bufonis Corium*	Y—Quality controlled by He’nan Institute of Traditional Chinese Medicine Research Hospital	N
SunJ 2022	Elemene Injectable Emulsion	Dalian Huali Jingang Pharmaceutical Co. Ltd.	*β-elemene*	Y- Prepared according to The Pharmacopoeia of the People’s Republic of China	Y- GC, HPLC
SunY 2019	Jiawei Yiguan Decoction	Huizhou Hospital of Traditional Chinese Medicine	*Rehmannia glutinosa (Gaertn*.*) DC*. *[Orobanchaceae]15g*, *Lycium barbarum L*. *[Solanaceae]15g*, *Glehnia littoralis (A*.*Gray) F*.*Schmidt ex Miq*. *[Apiaceae]15g*, *Ophiopogon japonicus (Thunb*.*) Ker Gawl*. *[Asparagaceae]15g*, *Angelica sinensis (Oliv*.*) Diels [Apiaceae]10g*, *Melia azedarach L*. *[Meliaceae]10g*, *Eupolyphaga sinensis Walker 5g*, *Prunus persica (L*.*) Batsch [Rosaceae]10g*, *Solanum nigrum L*. *[Solanaceae]5g*.	Y—Quality controlled by Huizhou Hospital of Traditional Chinese Medicine	N
TangYF 2018	Huai Er Granule	Qidong Gaitianli Medicines Co. Ltd.	*Pseudostellaria heterophylla (Miq*.*) Pax [Caryophyllaceae]*	Y- Prepared according to The Pharmacopoeia of the People’s Republic of China	Y- TLC
TuXL 2021	Jianpi Yanggan Jiedu Decoction	Ningbo Hospital of Traditional Chinese Medicine	*Astragalus mongholicus Bunge [Fabaceae] 30g*, *Codonopsis pilosula (Franch*.*) Nannf*. *[Campanulaceae] 20g*, *Atractylodes macrocephala Koidz*. *[Asteraceae] 20g*, *Coix lacryma-jobi L*. *[Poaceae] 30g*, *Smilax glabra Roxb*. *[Smilacaceae] 15g*, *Lycium barbarum L*. *[Solanaceae] 15g*, *Ligustrum lucidum W*.*T*.*Aiton [Oleaceae] 15g*, *Scutellaria baicalensis Georgi [Lamiaceae] 12g*, *Forsythia suspensa (Thunb*.*) Vahl [Oleaceae] 15*, *Lonicera japonica Thunb*. *[Caprifoliaceae] 15g*, *Fritillaria thunbergii Miq*. *[Liliaceae] 12g*, *Trionyx sinensis Wiegmann shell 20g*, *Isodon rubescens (Hemsl*.*) H*.*Hara [Lamiaceae] 30g*.	Y—Quality controlled by Ningbo Hospital of Traditional Chinese Medicine	N
WangGT 2016	Songxiang Bolus	The affiliated hospital of Shanxi university of Chinese medicine	*Abrus melanospermus subsp*. *melanospermus [Fabaceae]*	Y—Quality controlled by the affiliated hospital of Shanxi university of Chinese medicine	N
WuYW 2018	Jianpi Rougan Decoction	Guangdong Integrated Traditional Chinese and Western Medicine Hospital	*Codonopsis pilosula (Franch*.*) Nannf*. *[Campanulaceae] 15g*, *Galli Gigerii Endothelium Corneum 15g*, *Lycium barbarum L*. *[Solanaceae] 15g*, *Astragalus mongholicus Bunge [Fabaceae] 15g*, *Akebia trifoliata (Thunb*.*) Koidz*. *[Lardizabalaceae] 10g*, *Paeonia lactiflora Pall*. *[Paeoniaceae] 15g*, *Scrophularia ningpoensis Hemsl*. *[Scrophulariaceae] 10g*, *Curcuma aromatica Salisb*. *[Zingiberaceae] 15g*, *Atractylodes macrocephala Koidz*. *[Asteraceae] 15g*, *Bupleurum chinense DC*. *[Apiaceae] 10g*, *Curcuma longa L*. *[Zingiberaceae] 15g*, *Trionyx sinensis Wiegmann shell 15g*, *Pinellia ternata (Thunb*.*) Makino [Araceae] 10g*.	Y—Quality controlled by Guangdong Integrated Traditional Chinese and Western Medicine Hospital	N
YangCJ 2021	Jiawei Xiao Chaihu Granule	Sichuan Neo-Green Pharmaceutical Technology Development Co. Ltd.	*Bupleurum Chinense DC*. *[Apiaceae] 15g*, *Scutellaria baicalensis Georgi [Lamiaceae] 10g*, *Pinellia ternata (Thunb*.*) Makino [Araceae] 15g*, *Panax ginseng C*.*A*.*Mey*. *20g*, *Paeonia lactiflora Pall*. *[Paeoniaceae] 15g*, *Citrus × aurantium L*. *[Rutaceae] 15g*, *Citrus × aurantium L*. *[Rutaceae] 15g*, *Citrus × aurantium L*. *15g*, *Smilax glabra Roxb*. *[Smilacaceae] 15g*, *Atractylodes macrocephala Koidz*. *[Asteraceae] 15g*, *Zingiber officinale Roscoe [Zingiberaceae] 10g*, *Glycyrrhiza glabra L*. *[Fabaceae] 5g*.	Y- Prepared according to The Pharmacopoeia of the People’s Republic of China	Y- HPLC
YuJF 2021	Fuling Sini Decoction	Liuzhou Hospital of Traditional Chinese Medicine	*Smilax glabra Roxb*. *[Smilacaceae] 40g*, *Glycyrrhiza glabra L*. *[Fabaceae] 10g*, *Zingiber officinale Roscoe [Zingiberaceae] 10g*, *Raphanus raphanistrum subsp*. *sativus (L*.*) Domin [Brassicaceae] 15g*, *Panax ginseng C*.*A*.*Mey*. *10g*.	Y—Quality controlled by Liuzhou Hospital of Traditional Chinese Medicine	N
ZhangQH 2019	Huai Er Granule	Qidong Gaitianli Medicines Co. Ltd.	*Pseudostellaria heterophylla (Miq*.*) Pax [Caryophyllaceae]*	Y- Prepared according to The Pharmacopoeia of the People’s Republic of China	Y- TLC
ZhangZ 2019	Yiqi Huayu Jiedu Decoction	Hunan Academy of Traditional Chinese Medicine Affiliated Hospital	*Panax ginseng C*.*A*.*Mey*. *15g*, *Atractylodes macrocephala Koidz*. *[Asteraceae] 15g*, *Smilax glabra Roxb*. *[Smilacaceae] 15g*, *Curcuma aromatica Salisb*. *[Zingiberaceae]15g*, *Scutellaria barbata D*.*Don [Lamiaceae]15g*, *Astragalus mongholicus Bunge [Fabaceae] 30g*, *Eupolyphaga sinensis Walker*, *Paris polyphylla var*. *yunnanensis (Franch*.*) Hand*. *-Mazz*. *[Melanthiaceae]10g*, *Gekko gecko 10g*, *Trionyx sinensis Wiegmann shell 6g*, *Scorpion 5g*.	Y—Quality controlled by Hunan Academy of Traditional Chinese Medicine Affiliated Hospital	N
ZhanLH 2022	Chai Shao Granule	The first affiliated hospital of Guangxi University of Chinese Medicine	*Bupleurum Chinense DC*. *[Apiaceae]*, *Pinellia ternata (Thunb*.*) Makino [Araceae]*, *Scutellaria baicalensis Georgi [Lamiaceae]*, *Zingiber officinale Roscoe [Zingiberaceae]*, *Glycyrrhiza glabra L*. *[Fabaceae]*, *Ziziphus jujuba Mill*. *[Rhamnaceae]*, *Codonopsis pilosula (Franch*.*) Nannf*. *[Campanulaceae]*, *Paeonia lactiflora Pall*. *[Paeoniaceae]*, *Angelica sinensis (Oliv*.*) Diels [Apiaceae]*, *Alisma plantago-aquatica subsp*. *Orientale (Sam*.*) Sam*. *[Alismataceae]*, *Smilax glabra Roxb*. *[Smilacaceae]*, *Conioselinum anthriscoides ’Chuanxiong’ [Apiaceae]*, *Atractylodes macrocephala Koidz*. *[Asteraceae]*.	Y—Quality controlled by the first affiliated hospital of Guangxi University of Chinese Medicine	N
ZhouFJ 2020	Jiajian Sanjia Powder	Handan Hospital of Traditional Chinese Medicine	*Trionyx sinensis Wiegmann shell 10g*, *Astragalus mongholicus Bunge [Fabaceae] 10g*, *Angelica sinensis (Oliv*.*) Diels [Apiaceae] 10g*, *Manis pentadactyla Linnaeus 10g*, *Eupolyphaga sinensis Walker 10g*, *Beauveria bassiana (Bals*.*) Vuillant 10g*, *Prunus persica (L*.*) Batsch [Rosaceae] 10g*, *Bupleurum chinense DC*. *[Apiaceae] 10g*.	Y—Quality controlled by Handan Hospital of Traditional Chinese Medicine	N
ZhuX 2023	Danzhi Xiaoyao Granule	Cangzhou Hospital of Integrated Chinese and Western Medicine	*Paeonia × suffruticosa Andrews [Paeoniaceae] 9g*, *Gardenia jasminoides J*.*Ellis [Rubiaceae] 9g*, *Angelica sinensis (Oliv*.*) Diels [Apiaceae] 12g*, *Paeonia lactiflora Pall*. *[Paeoniaceae] 12g*, *Bupleurum Chinense DC*. *[Apiaceae] 12g*, *Smilax glabra Roxb*. *[Smilacaceae] 15g*, *Atractylodes macrocephala Koidz*. *[Asteraceae] 15g*, *Glycyrrhiza glabra L*. *[Fabaceae] 6g*, *Zingiber officinale Roscoe [Zingiberaceae] 6g*, *Mentha spicata L*. *[Lamiaceae] 6g*, *Trionyx sinensis Wiegmann shell 12g*, *Fritillaria thunbergii Miq*. *[Liliaceae] 12g*, *Curcuma aromatica Salisb*. *[Zingiberaceae] 9g*.	Y—Quality controlled by Cangzhou Hospital of Integrated Chinese and Western Medicine	N

### Investigation quality evaluation

**Fig 3:**
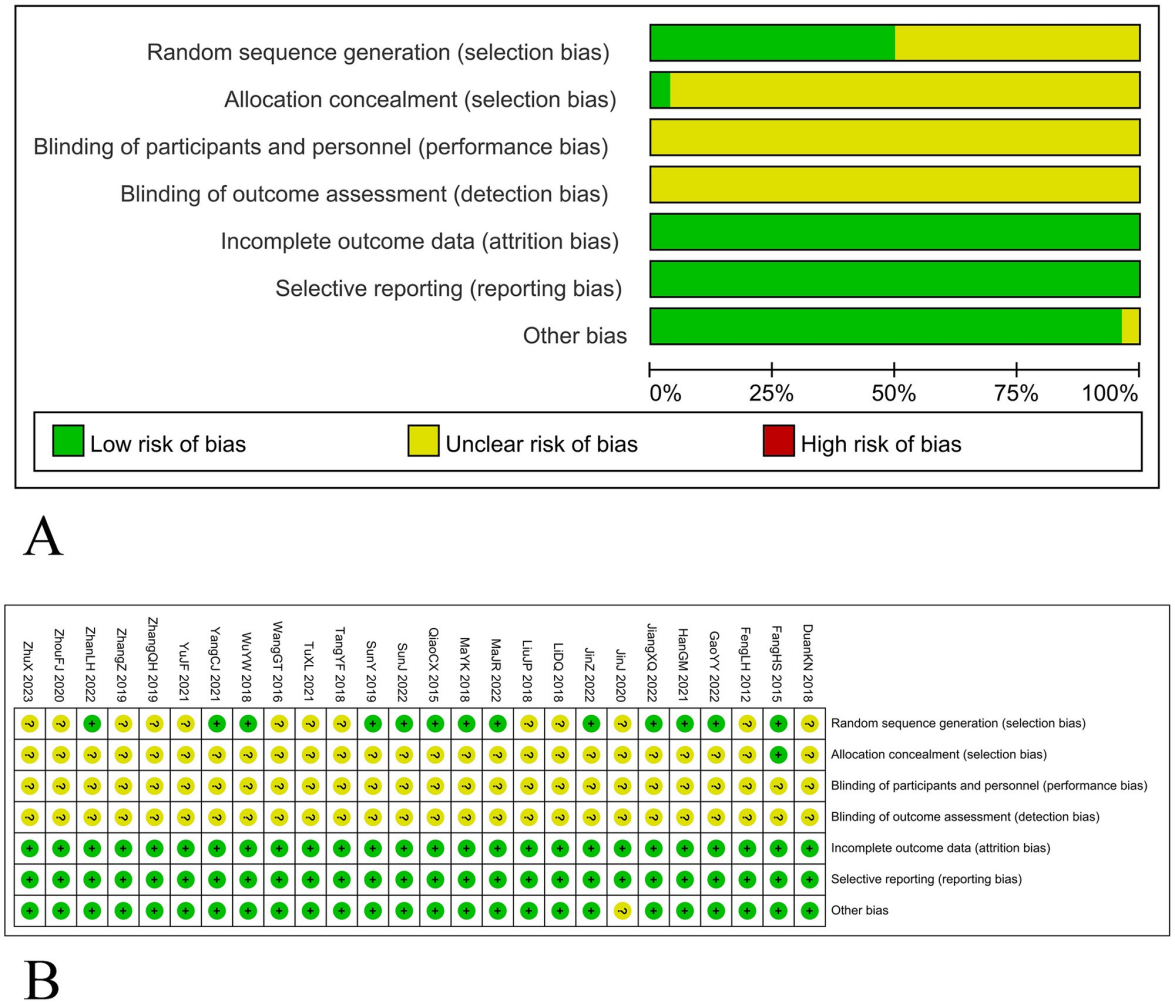
Assessment of risk of bias in incorporated investigations. (A) Graphical representation for the type of risk and their distribution. (B) Table summarizing the level of bias for all studies.

To determine dataset quality within 26 enrolled studies, their risk of bias was assessed (Fig 3, S4 File). The “random sequence generation” domain was evaluated using the random number table method and revealed 13 studies [44, 46–48, 50, 53–57, 61, 62, 64] with low risk of bias while the remaining studies indicated unclear risk due to the lack of specific methods. For “allocation concealment” assessed with the envelope method, only one study [44] had a low risk of bias, while the remaining indicated unclear risk because they did not report the blinding methods. Furthermore, all studies had a low risk of bias for “incomplete outcome data” since all data were fairly complete. Moreover, for “selective reporting”, all studies had a low risk of bias as they all employed standard analysis methods. Under the “other bias” category, only one study [49] showed an unclear risk of bias because patients’ age was not reported at baseline.

### Primary outcome

#### Objective response rate / Disease control rate

**Fig 4:**
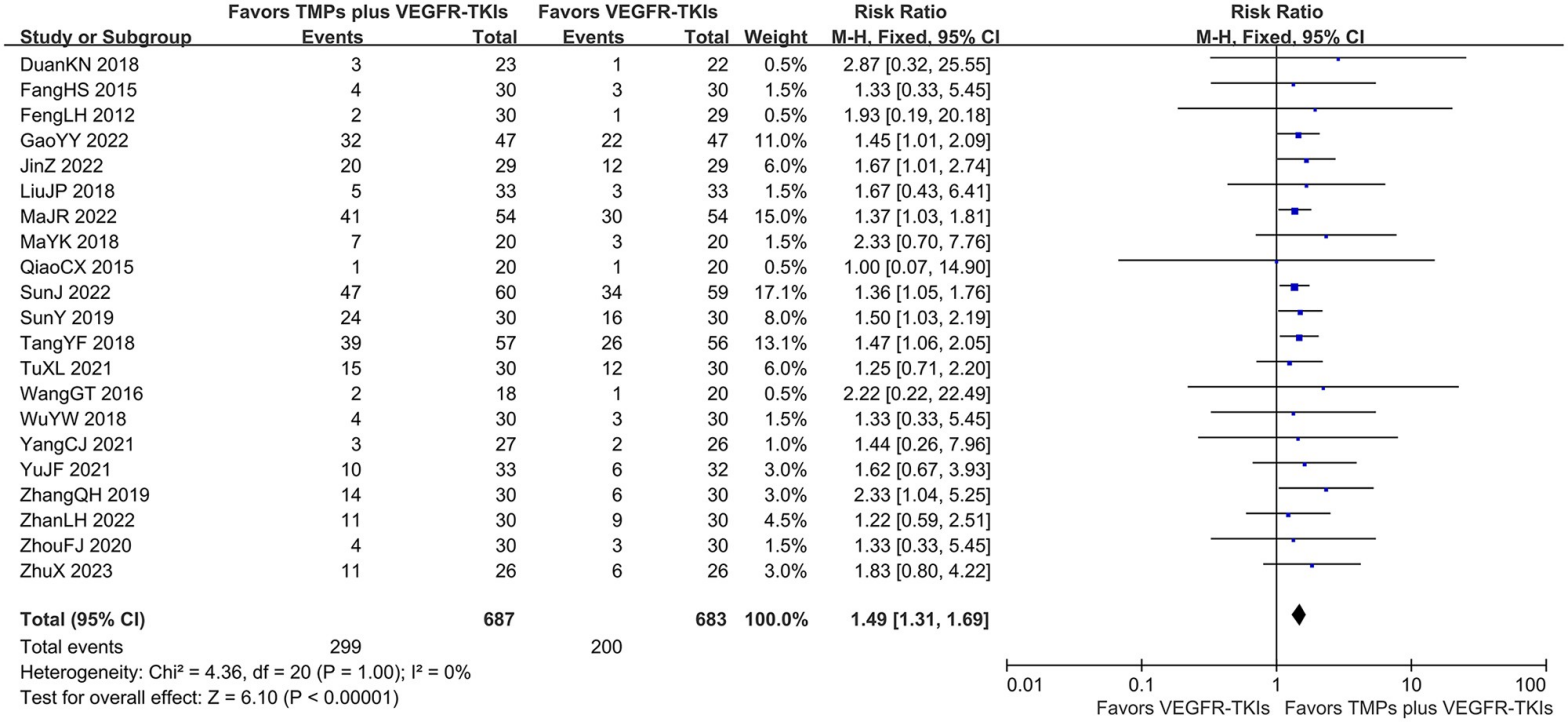
Forest-plot/pooled RRs comparing the influence of TMPs + VEGFR-TKIs and VEGFR-TKIs on ORR. Risk ratio represents the ratio of the studied outcome between the co-treatment group and the mono treatment group.

**Fig 5:**
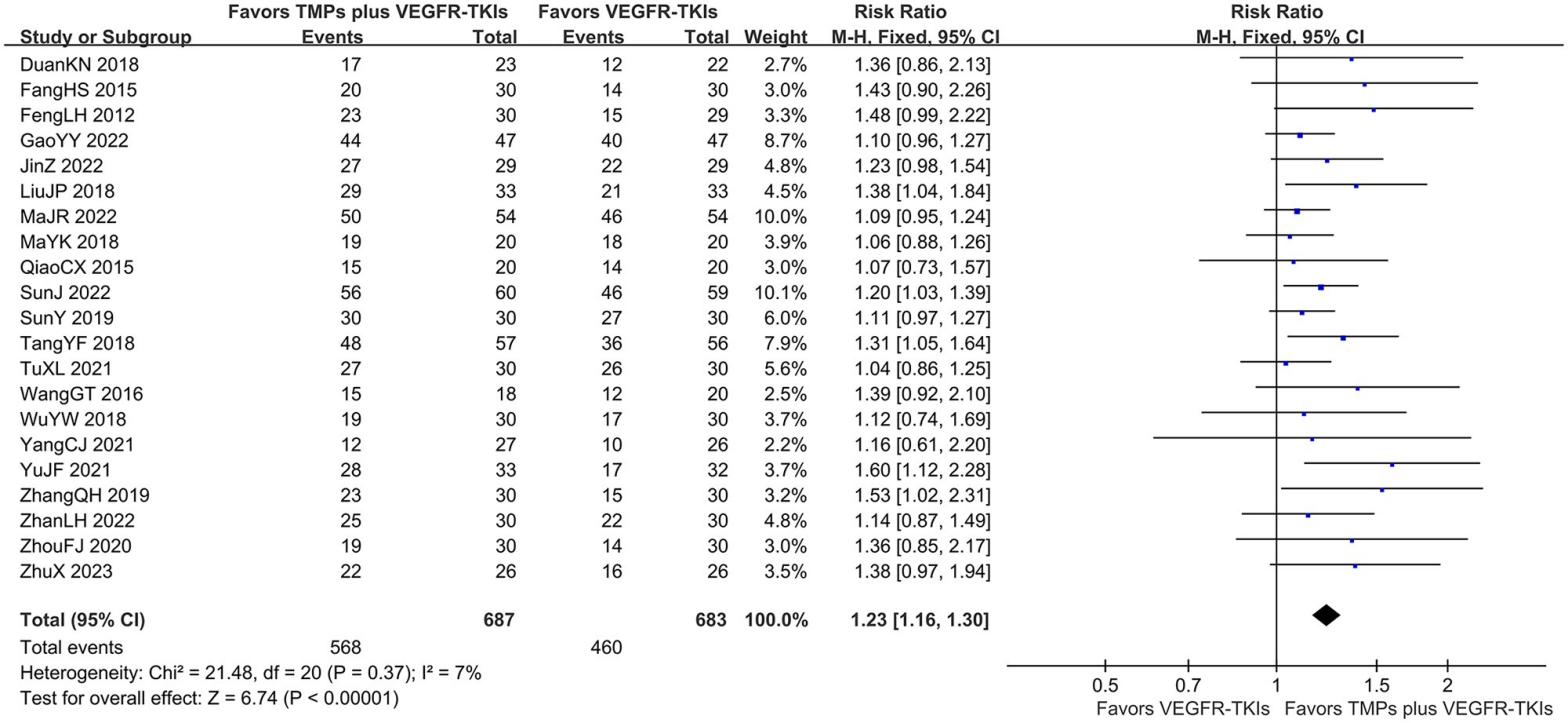
Forest-plot/pooled RRs comparing the influence of TMPs + VEGFR-TKIs and VEGFR-TKIs on DCR.

Of the 26 selected studies, only 21 reported ORR/DCR for a total of 1370 patients. Furthermore, the meta-analysis revealed that TMPs+ VEGFR-TKIs co-treatment significantly increased the ORR by 14.3% (FEM; RR = 1.49; 95% CI: 1.31–1.69; *I*^*2*^ *=* 0%; *p* < 0.00001; Fig 4), and DCR by 15.5% (FEM, RR = 1.23; 95% CI: 1.16–1.30; *I*^*2*^ *=* 7%; *p* < 0.00001; Fig 5) compared to VEGRR-TKI mono-treatment.

#### Sub-cohort assessment for tumor response

Sub-cohort assessments for ORR and DCR were performed for the target drug regimen, treatment course, KPS score, and TMP form (Tables 3 and 4). The target drug regimen was categorized into sorafenib, apatinib, lenvatinib, and regorafenib. The sub-cohort analyses revealed that combining TMPs with lenvatinib can achieve better ORR (S1 Fig in S5 File). Furthermore, the TMP was given as a decoction, granule, pill, powder, bolus, and injection. The sub-cohort analysis revealed that the decoction form of TMPs can improve ORR better (S2 Fig in S5 File). Categorizing the treatment course [duration in months (M)] into < 2 and ≥ 2 M did not significantly affect the outcome (S3 Fig in S5 File). Furthermore, a subdivision of the KPS score to ≥ 60 and unclear did not significantly change the outcome (S4 Fig in S5 File). For DCR, the sorafenib sub-cohort indicated a better effect (S5 Fig in S5 File). Moreover, in DCR, the source of heterogeneity was the target drug regimen (sub-cohort difference analysis *p*_*h*_ *=* 0.02; *I*_*h*_^*2*^ *=* 68.9). In addition, the DCR of the remaining sub-cohorts had higher heterogeneity than the total pool (S6–S8 Figs in S5 File). Therefore, the sub-cohorts indicated no convincing results except for the targeted drug treatment schedule.

**Table 3:** Subgroup analysis of the ORR.

Subgroup	Number of trials	RR,95%CI	Z	P	Heterogeneity	
					*I* ^*2*^	*P*_*h*_
Sorafenib	12	1.52 [1.25, 1.85]	4.14	< 0.0001	0%	1.00
Lenvatinib	3	1.53 [1.09, 2.16]	2.44	0.01	0%	0.68
Apatinib	5	1.47 [1.15, 1.89]	3.06	0.002	0%	0.93
Regorafenib	1	1.37 [1.03, 1.81]	2.17	0.03	Not applicable	Not applicable
1M≤and<2M	5	1.47 [1.04, 2.09]	2.18	0.03	0%	1.00
≥2M	16	1.49 [1.30, 1.70]	6.10	< 0.00001	0%	1.00
Decoction	11	1.52 [1.24, 1.87]	3.69	< 0.0001	0%	1.00
Granule	6	1.50 [1.22, 1.83]	3.93	< 0.0001	0%	0.84
Powder	1	1.33 [0.33, 5.45]	0.40	0.69	Not applicable	Not applicable
Injection	1	1.36 [1.05, 1.76]	2.35	0.02	Not applicable	Not applicable
Pill	1	1.93 [0.19, 20.18]	0.55	0.58	Not applicable	Not applicable
Bolus	1	2.22 [0.22, 22.49]	0.68	0.50	Not applicable	Not applicable
≥60	11	1.50 [1.22, 1.84]	3.82	0.0001	0%	0.99
Unclear	9	1.46 [1.23, 1.73]	4.34	< 0.0001	0%	0.99

**Table 4:** Subgroup analysis of the DCR.

Subgroup	Number of trials	RR,95%CI	Z	P	Heterogeneity	
					*I* ^*2*^	*P*_*h*_
Sorafenib	12	1.34 [1.21, 1.47]	5.89	< 0.0001	0%	0.91
Lenvatinib	3	1.19 [1.03, 1.37]	2.39	0.02	29%	0.25
Apatinib	5	1.10 [1.00, 1.22]	2.04	0.04	0%	0.99
Regorafenib	1	1.09 [0.95, 1.24]	1.22	0.22	Not applicable	Not applicable
1M≤and<2M	5	1.29 [1.11, 1.51]	3.26	0.001	44%	0.13
≥2M	16	1.21 [1.14, 1.29]	5.91	< 0.00001	0%	0.47
Decoction	11	1.20 [1.11, 1.30]	4.40	< 0.0001	16%	0.30
Granule	6	1.23 [1.10, 1.38]	3.71	0.0002	9%	0.36
Powder	1	1.36 [0.85, 2.17]	1.27	0.20	Not applicable	Not applicable
Injection	1	1.20 [1.03, 1.39]	2.33	0.02	Not applicable	Not applicable
Pill	1	1.48 [0.99, 2.22]	0.06	1.91	Not applicable	Not applicable
Bolus	1	1.39 [0.92, 2.10]	1.56	0.12	Not applicable	Not applicable
≥60	11	1.27 [1.14, 1.41]	4.48	< 0.00001	12%	0.33
Unclear	9	1.20 [1.11, 1.29]	4.78	< 0.00001	20%	0.27

### Secondary outcomes

#### One-year overall survival

**Fig 6:**
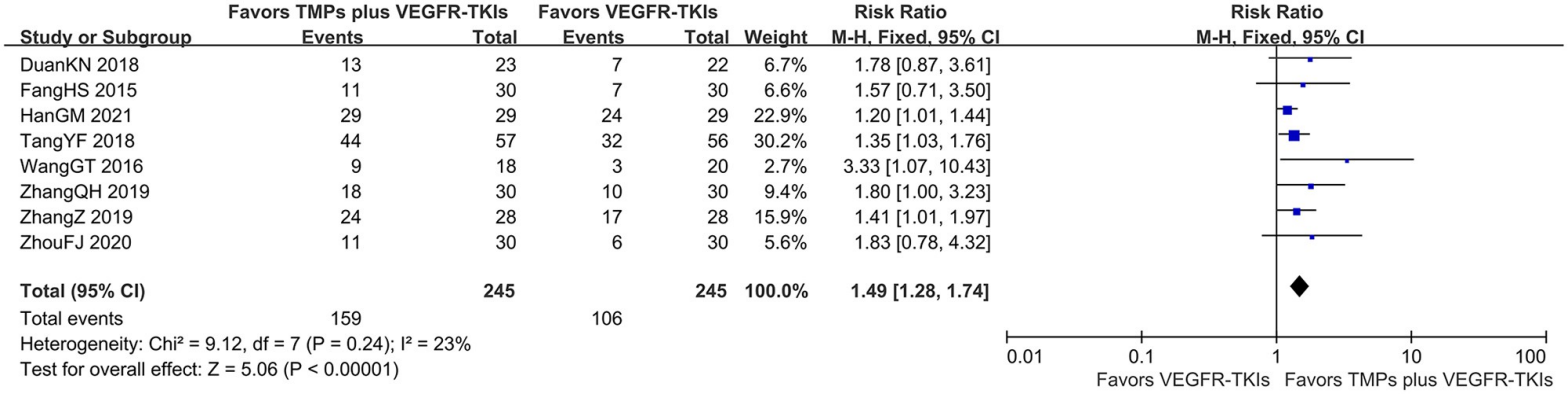
Forest-plot/pooled RRs comparing the influence of TMPs + VEGFR-TKIs and VEGFR-TKIs on one-year OS.

A total of 8 studies reported one-year OS was reported for 490 patients. The meta-analysis revealed that, similar to the effect on ORR, the addition of TMPs significantly increased one-year OS by 21.2% (FEM; RR = 1.49; 95% CI: 1.28–1.74; *I*^*2*^ *=* 23%; *p* < 0.00001) compare to VEGFR-TKI treatment alone (Fig 6).

#### Quality of life

The dichotomous KPS data were reported by 3 studies [43, 52, 61] for 171 patients. The meta-analysis presented no significant difference between TMPs + VEGFR-TKIs co-treatment and TKIs mono-treatment (FEM; RR = 1.12; 95% CI: 0.84–1.49; *I*^*2*^ *=* 0%; *p* = 0.44; S9 Fig in S5 File). Continuous KPS data were reported in 8 studies [47, 48, 58, 59, 61, 62, 64, 68] for 531 patients, but, the significant heterogeneity in these data prevented a meta-analysis. However, these eight studies indicated higher KPS scores in the co-treatment cohort compared to controls (S1 Table in S5 File).

#### Alpha fetoprotein

Continuous AFP data were documented in 13 studies comprising 899 patients (S2 Table in S5 File). Nine studies [44, 46, 48, 50, 52, 58, 60, 64, 67] reported data in mean ± SD, while four [53, 61, 62, 68] reported the median and interquartile range. Unfortunately, the data were too heterogeneous to allow a meta-analysis. However, the consensus is that TMP addition can decrease AFP levels in patients compared with VEGFR-TKIs alone, as reported in ten studies. The remaining three investigations [61, 62, 64] indicated no major variations between the co-and mono-treatment. Only one study [54] identified AFP through dichotomous data, which also supports that combination treatment decreased AFP levels more than VEGFR-TKIs alone.

#### Adverse drug reactions

The incidence of different ADRs is indicated in S3 Table within S5 File. Hypertension [44–46, 49, 52–54, 56, 58, 61, 62, 64, 65], hand-foot skin reactions [44, 46, 49, 52–54, 56, 61, 62, 64–67], and gastrointestinal reactions [44, 45, 50–53, 56, 58, 62, 64–67] were the most commonly reported ADR, reported in 14 studies. Followed by liver dysfunction, reported in 13 studies with 815 patients [5 dichotomous data [45, 49, 56, 61, 62], 8 continuous data [44, 48, 51–53, 60, 64, 67]]. This was followed by myelosuppression [10 studies [44–46, 49, 54, 61, 62, 64, 66, 67]], fatigue [7 studies [44, 46, 52, 56, 64, 66, 67]], proteinuria [7 studies [46, 54, 61, 62, 64]] and rash [6 studies [44, 52, 58, 62, 65, 67]].

**Fig 7:**
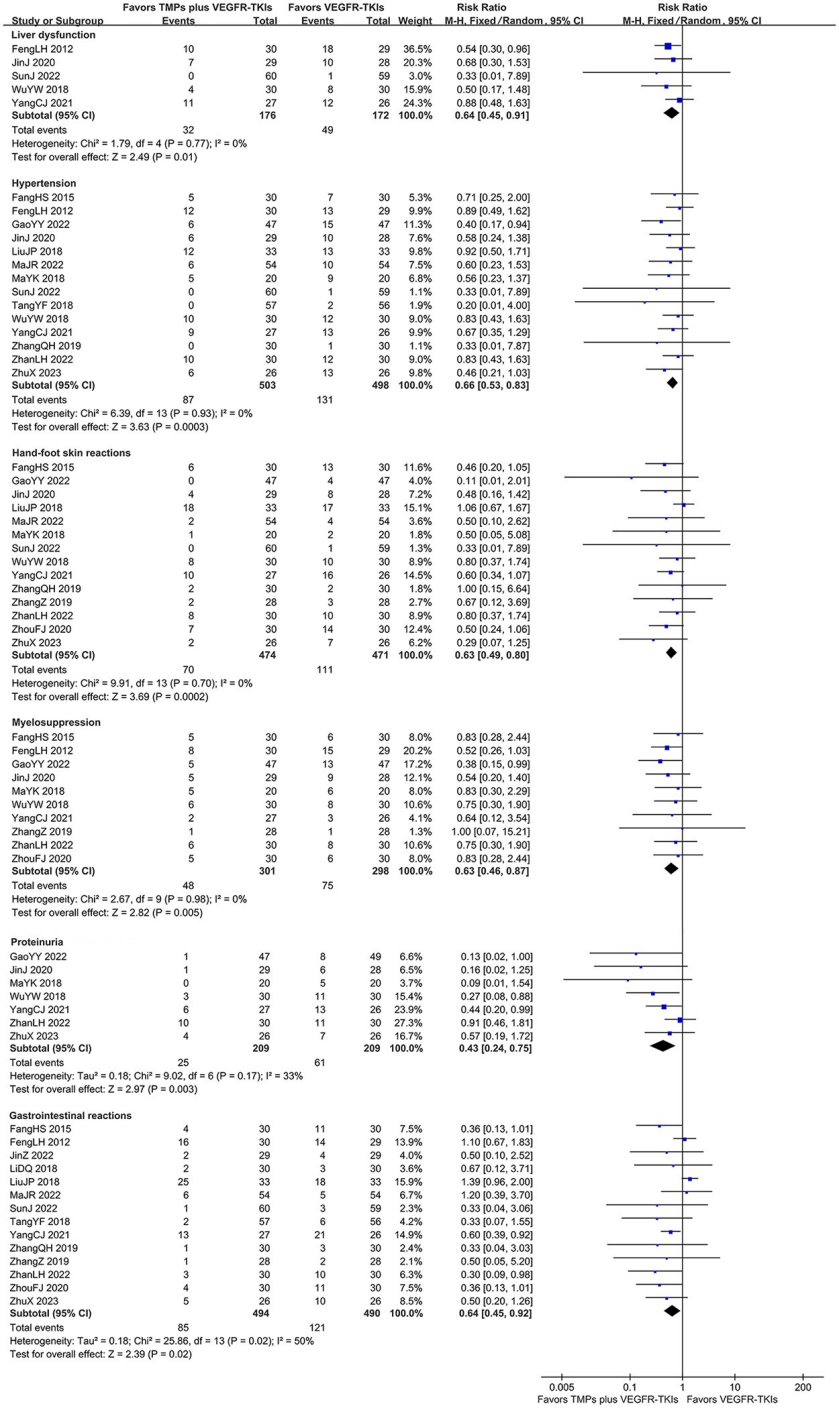
Forest-plot/pooled RRs comparing the influence of TMPs + VEGFR-TKIs and VEGFR-TKIs on ADRs.

Meta-analysis revealed that compared to TKI mono-treatment, co-treatment reduce six manifestations from the eight evaluated ADRs, including liver dysfunction (dichotomous data: FEM, RR = 0.64, 95% CI: 0.45–0.91, 348 participants, *I*^*2*^ *=* 0%, *p* = 0.01), hypertension (FEM, RR = 0.66, 95% CI: 0.53–0.83, 1001 participants, *I*^*2*^ = 0%, *p* = 0.0003), hand-foot skin reactions (FEM, RR = 0.63, 95% CI: 0.49–0.80, 945 participants, *I*^*2*^ = 0%, *p* = 0.0002), gastrointestinal reactions (REM, RR = 0.64, 95% CI: 0.45–0.92, 984 participants, *I*^*2*^ = 50%, *p* = 0.02), myelosuppression (FEM, RR = 0.63, 95% CI: 0.46–0.87, 599 participants, *I*^*2*^ = 0%, *p* = 0.005), and proteinuria (REM, RR = 0.43, 95% CI: 0.24–0.75, 418 participants, *I*^*2*^ = 33%, *p* = 0.003). The meta-analysis could not be performed for a continuous dataset for liver dysfunction (8 studies with 467 patients) because of the great heterogeneity. However, all eight studies showed lower levels of alanine aminotransferase in the co-treatment cohort compared to the control cohort post-therapy. In addition, the co-treatment cohort did not exhibit an increased incidence of rash and fatigue (S10 Fig in S5 File). Meta-analysis outcomes for ADRs are depicted in S4 Table within S5 File and Fig 7.

#### Influence of independent traditional medicine-based compounds on oral TMP cohort

Oral TMPs included 105 different traditional medicine-derived compounds with a mean quantity of nine constituents for each TMP therapy. The peak frequency of traditional medicines use was as follows: *Atractylodes macrocephala* Koidz. [Asteraceae] (n = 12), *Astragalus mongholicus* Bunge [Fabaceae] (n = 9), *Trionyx sinensis* Wiegmann shell (n = 9), *Bupleurum chinense* DC. [Apiaceae] (n = 8), and *Panax ginseng* C.A.Mey. (n = 7). The sub-cohort analyses included 36 traditional medicines used in at least two studies. Of these, 10 traditional medicines indicated significant RRs for increasing ORR with low heterogeneity (*I*^*2*^ < 30%). These 10 were further assessed for combinations of pairs, triplets, or more with TMPs. Tables 5 and 6 present the sub-cohort analysis of ORR and DCR for these traditional medicines and their combinations.

**Table 5:** Effects of specific TMPs on ORR for PLC: Single medicine and combinations.

Level	Traditional medicine	N studies	N participants	RR (95% CI)	*I* ^*2*^
1	Panax ginseng C.A.Mey.	6	316	1.65 [1.12, 2.44]	0
1	Trametes robiniophila Murr.	2	173	1.63 [1.19, 2.24]	10
1	*Curcuma aromatica* Salisb. [Zingiberaceae]	4	246	1.59 [1.15, 2.20]	0
1	*Solanum nigrum* L. [Solanaceae]	2	105	1.58 [1.07, 2.34]	0
1	*Rehmannia glutinosa* (Gaertn.) DC. [Orobanchaceae]	2	105	1.58 [1.07, 2.34]	0
1	*Angelica sinensis* (Oliv.) Diels [Apiaceae]	5	390	1.56 [1.20, 2.02]	0
1	Citrus × aurantium L. [Rutaceae]	3	187	1.55 [1.09, 2.20]	0
1	*Prunus persica* (L.) Batsch [Rosaceae]	4	238	1.53 [1.13, 2.07]	0
1	*Paeonia lactiflora* Pall. [Paeoniaceae]	5	276	1.51 [1.02, 2.23]	0
1	Eupolyphaga sinensis Walker	5	290	1.50 [1.08, 2.08]	0
2	*Curcuma aromatica* Salisb. [Zingiberaceae] + *Angelica sinensis* (Oliv.) Diels [Apiaceae]	2	92	2.00 [1.01, 3.97]	0
2	*Panax ginseng* C.A.Mey. + *Angelica sinensis* (Oliv.) Diels [Apiaceae]	3	158	1.72 [1.10, 2.70]	0
2	Eupolyphaga sinensis Walker + Solanum nigrum L. [Solanaceae]	2	105	1.58 [1.07, 2.34]	0
2	*Eupolyphaga sinensis* Walker + *Rehmannia glutinosa* (Gaertn.) DC. [Orobanchaceae]	2	105	1.58 [1.07, 2.34]	0
2	*Solanum nigrum* L. [Solanaceae] + *Rehmannia glutinosa* (Gaertn.) DC. [Orobanchaceae]	2	105	1.58 [1.07, 2.34]	0
2	*Curcuma aromatica* Salisb. [Zingiberaceae] + *Citrus × aurantium* L. [Rutaceae]	2	134	1.56 [1.09, 2.22]	0
2	*Citrus × aurantium* L. [Rutaceae] + *Pinellia ternata* (Thunb.) Makino [Araceae]	3	187	1.55 [1.09, 2.20]	0
2	*Curcuma aromatica* Salisb. [Zingiberaceae] + *Pinellia ternata* (Thunb.) Makino [Araceae]	3	194	1.54 [1.08, 2.18]	0
2	*Angelica sinensis* (Oliv.) Diels [Apiaceae] + *Prunus persica* (L.) Batsch [Rosaceae]	4	238	1.53 [1.13, 2.07]	0
3	*Eupolyphaga sinensis* Walker + *Solanum nigrum* L. [Solanaceae] + *Rehmannia glutinosa* (Gaertn.) DC. [Orobanchaceae]	2	105	1.58 [1.07, 2.34]	0
3	*Curcuma aromatica* Salisb. [Zingiberaceae] + *Pinellia ternata* (Thunb.) Makino [Araceae] + *Citrus × aurantium* L. [Rutaceae]	2	134	1.56 [1.09, 2.22]	0

**Table 6:** Effects of specific TMPs on DCR for PLC: Single herb and combinations.

Level	Traditional medicine	N studies	N participants	RR (95% CI)	I^2^
1	Scutellaria barbata D.Don [Lamiaceae]	3	171	1.39 [1.11,1.73]	0
1	Trametes robiniophila Murr.	2	173	1.38 [1.13,1.68]	0
1	Scolopendra subspinipes mutilans	2	111	1.37 [1.07,1.75]	0
1	Manis pentadactyla Linnaeus	2	126	1.37 [1.06,1.77]	0
1	Glycyrrhiza glabra L. [Fabaceae]	6	398	1.25 [1.10, 1.41]	29
1	*Prunus persica* (L.) Batsch [Rosaceae]	4	238	1.25 [1.07,1.44]	11
2	Scutellaria barbata D.Don [Lamiaceae] + Scolopendra subspinipes mutilans	2	111	1.37 [1.07,1.75]	0

*Sub-cohort analysis level 1*. *Single traditional medicines*. For ORR, the RRs of eight traditional medicines were higher than the total pool. *Panax ginseng* C.A.Mey. indicated the highest RR, followed by *Trametes robiniophila* Murr. (huaier), *Curcuma aromatica* Salisb. [Zingiberaceae], *Solanum nigrum* L. [Solanaceae], *Rehmannia glutinosa* (Gaertn.) DC. [Orobanchaceae], *Angelica sinensis* (Oliv.) Diels [Apiaceae], *Citrus × aurantium* L. [Rutaceae], *Prunus persica* (L.) Batsch [Rosaceae], *Paeonia lactiflora* Pall. [Paeoniaceae], and *Eupolyphaga sinensis* Walker.

For DCR, six traditional medicines had higher RRs than the total pool, with *Scutellaria barbata* D.Don [Lamiaceae] indicating the highest RR, followed by *Trametes robiniophila* Murr. (huaier), *Scolopendra subspinipes mutilans*, *Manis pentadactyla* Linnaeus, *Glycyrrhiza glabra* L. [Fabaceae], and *Prunus persica* (L.) Batsch [Rosaceae].

*Sub-cohort analysis level 2*: *Pairs of traditional medicines*. For ORR, nine pairs of traditional medicines had higher RRs than the total pool. The highest RR was for *Curcuma aromatica* Salisb. [Zingiberaceae] + *Angelica sinensis* (Oliv.) Diels [Apiaceae], followed by *Panax ginseng* C.A.Mey. + *Angelica sinensis* (Oliv.) Diels [Apiaceae], *Eupolyphaga sinensis* Walker + *Solanum nigrum* L. [Solanaceae], *Eupolyphaga sinensis* Walker + *Rehmannia glutinosa* (Gaertn.) DC. [Orobanchaceae], *Solanum nigrum* L. [Solanaceae] + *Rehmannia glutinosa* (Gaertn.) DC. [Orobanchaceae], *Curcuma aromatica* Salisb. [Zingiberaceae] + *Citrus × aurantium* L. [Rutaceae], *Citrus × aurantium* L. [Rutaceae] + *Pinellia ternata* (Thunb.) Makino [Araceae], *Angelica sinensis* (Oliv.) Diels [Apiaceae] + *Prunus persica* (L.) Batsch [Rosaceae], and *Curcuma aromatica* Salisb. [Zingiberaceae] + *Pinellia ternata* (Thunb.) Makino [Araceae]. Whereas, for DCR, only one pair of traditional medicines, *Scutellaria barbata* D.Don [Lamiaceae] + *Scolopendra subspinipes mutilans*, met the inclusion criteria.

*Sub-cohort analysis level 3*: *Triplets of traditional medicines*. It was observed that RRs for two triplets of traditional medicines were higher than the total pool. The highest RR was for *Eupolyphaga sinensis* Walker + *Solanum nigrum* L. [Solanaceae] + *Rehmannia glutinosa* (Gaertn.) DC. [Orobanchaceae], followed by *Curcuma aromatica* Salisb. [Zingiberaceae] + *Pinellia ternata* (Thunb.) Makino [Araceae] + *Citrus × aurantium* L. [Rutaceae]. No triplet combinations were found for DCR.

### Publication bias

**Fig 8:**
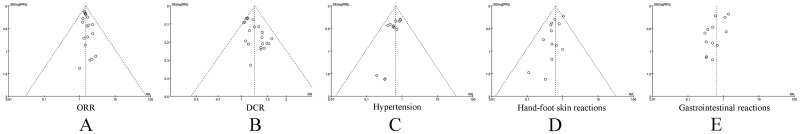
Assessment of publication bias. Funnel plots comparing TMPs + VEGFR-TKIs with VEGFR-TKIs mono-treatment for (A) ORR (21 trials), (B) DCR (21 trials), (C) hypertension (14 trials), (D) hand-foot skin reactions (14 trials), and (E) gastrointestinal reactions (14 trials).

Funnel plots comparing TMPs + VEGFR-TKIs co-treatment and VEGFR-TKIs mono-treatment for ORR, DCR, hypertension, hand-foot skin reactions, and gastrointestinal reactions showed symmetric distribution on both sides (Fig 8), indicating no significant publication bias. These data were validated by Begg’s test, which indicated insignificant publication bias for ORR (*p = 0*.*3323*), hand-foot skin reactions (*p = 0*.*3506*), and gastrointestinal reactions (*p = 0*.*9562*). However, potential publication bias was indicated for DCR (*p = 0*.*0852*), and hypertension (*p = 0*.*0546*). Following the results of the funnel plot and Begg’s test, we further conducted Egger’s tests for hypertension (*p = 0*.*1405*, *intercept = 0*.*0526*) and hand-foot skin reactions (*p = 0*.*0952*, *intercept = 0*.*0180*) to enhance the rigor of our assessment. A significance level of *p < 0*.*1* is recommended for detecting publication bias in both Begg’s and Egger’s tests [69]. After a comprehensive evaluation, we concluded that there is potential publication bias in the results for DCR, hypertension, and hand-foot skin reactions.

### Sensitivity analyses

Since both random and fixed effect models were employed in this meta-analysis, determining whether switching between them would significantly alter the outcome was important. Sensitivity analyses showed that the acquired results were stable when switching between these modeling methods.

### Quality of evidence

The quality of evidence was assessed using the GRADE criteria. Data on ORR, one-year OS, and myelosuppression were rated moderate, while that on DCR, hypertension, hand-foot skin reactions, liver dysfunction, gastrointestinal reactions, and proteinuria were rated low, whereas data on the quality-of-life was rated very low (Table 7).

**Table 7:** Summary of finding table of TMPs plus VEGFR-TKIs compared to VEGFR-TKIs alone for PLC.

**TMPs plus VEGFR-TKIs compared to VEGFR-TKIs alone for PLC**					
**Patient or population:** Middle-advanced Primary Liver Cancer **Setting:** Outpatient department/Inpatient department **Intervention:** TMPs plus VEGFR-TKIs **Comparison**: VEGFR-TKIs					
Outcome № of participants (studies)	Relative effect (95% CI)	Anticipated absolute effects (95% CI)			Certainty
		VEGFR-TKIs alone	TMPs plus VEGFR-TKIs	Difference	
ORR № of participants: 1370 (21 RCTs)	RR 1.49 (1.31 to 1.69)	29.3%	43.6% (38.4 to 49.5)	14.3% more (9.1 more to 20.2 more)	⨁⨁⨁◯ Moderate^a^
DCR № of participants: 1370 (21 RCTs)	RR 1.23 (1.16 to 1.30)	67.3%	82.8% (78.1 to 87.6)	15.5% more (10.8 more to 20.2 more)	⨁⨁◯◯ Low^a^^,^^b^
One-year OS № of participants: 490 (8 RCTs)	RR 1.49 (1.28 to 1.74)	43.3%	64.5% (55.4 to 75.3)	21.2% more (12.1 more to 32 more)	⨁⨁⨁◯ Moderate^a^
KPS (dichotomous data) № of participants: 171 (3 RCTs)	RR 1.12 (0.84 to 1.49)	41.2%	46.1% (34.6 to 61.4)	4.9% more (6.6 fewer to 20.2 more)	⨁◯◯◯ Very low^a^^,^^c^^,^^d^
Liver dysfunction № of participants: 348 (5 RCTs)	RR 0.64 (0.45 to 0.91)	28.5%	18.2% (12.8 to 25.9)	10.3% fewer (15.7 fewer to 2.6 fewer)	⨁⨁◯◯ Low^a^^,^^e^
Proteinuria № of participants: 418 (7 RCTs)	RR 0.43 (0.24 to 0.75)	29.2%	12.6% (7 to 21.9)	16.6% fewer (22.2 fewer to 7.3 fewer)	⨁⨁◯◯ Low^a^^,^^f^
Hypertension № of participants: 1001 (14 RCTs)	RR 0.66 (0.53 to 0.83)	26.3%	17.4% (13.9 to 21.8)	8.9% fewer (12.4 fewer to 4.5 fewer)	⨁⨁◯◯ Low^a^^,^^b^
Hand-foot skin reactions № of participants: 945 (14 RCTs)	RR 0.63 (0.49 to 0.80)	23.6%	14.8% (11.5 to 18.9)	8.7% fewer (12 fewer to 4.7 fewer)	⨁⨁◯◯ Low^a^^,^^b^
Myelosuppression № of participants: 599 (10 RCTs)	RR 0.63 (0.46 to 0.87)	25.2%	15.9% (11.6 to 21.9)	9.3% fewer (13.6 fewer to 3.3 fewer)	⨁⨁⨁◯ Moderate^a^
Gastrointestinal reactions № of participants: 984 (14 RCTs)	RR 0.64 (0.45 to 0.92)	24.7%	15.8% (11.1 to 22.7)	8.9% fewer (13.6 fewer to 2 fewer)	⨁⨁◯◯ Low^a^^,^^f^
*The risk in the intervention group (and its 95% confidence interval) is based on the assumed risk in the comparison group and the **relative effect** of the intervention (and its 95% CI). **CI:** confidence interval; **RR:** risk ratio					
GRADE Working Group grades of evidence **High certainty:** we are very confident that the true effect lies close to that of the estimate of the effect. **Moderate certainty:** we are moderately confident in the effect estimate: the true effect is likely to be close to the estimate of the effect, but there is a possibility that it is substantially different. **Low certainty:** our confidence in the effect estimate is limited: the true effect may be substantially different from the estimate of the effect. **Very low certainty:** we have very little confidence in the effect estimate: the true effect is likely to be substantially different from the estimate of effect.					

Explanations

^a^. There were serious limitations of methodological quality among trials according to the risk of bias assessment.

^b^. There was potential publication bias.

^c^. Too small simple size.

^d^. There was no difference between the experience group and control group according to the *p-value*.

^e^. There was no significant difference between the experience group and control group according to the *p-value*.

^f^. There was significant statistical heterogeneity among trials according to I^2^

## Discussion

### Predominant review outcomes

In this review, the data included 26 studies indicated was mostly uncertain, had low risk of bias, and was of moderate quality. The meta-analysis revealed that co-therapy of TMPs + VEGFR-TKIs considerably increased the ORR and DCR compared to VEGFR-TKIs alone. This might be because TMP can inhibit HCC cell proliferation and migration [29], suppress HCC cell cycle [70], and reduce angiogenesis [71]. Pharmacological research has indicated that nobiletin, an extract from *Citrus × aurantium*, can suppress HCC cell proliferation and apoptosis by downregulating Bcl-2 and COX-2, as well as upregulating Bax and caspase-3 [26]. Furthermore, catalpol, an *R*. *glutinosa* extract, can synergistically increase the antitumor effects of regorafenib in HepG2 and HUH-7 HCC cell lines by inhibiting VEGF/VEGFR2, PI3K/Akt/mTOR, and NF-κB signaling pathways [72].

It has been revealed that in terms of OS, lenvatinib is comparable to sorafenib clinically, but has improved ORR [9]. This investigation revealed that TMPs + lenvatinib had better ORR than TMP + other VEGFR-TKIs. Regarding DCR, this investigation revealed that TMPs + sorafenib co-treatment was better than TMP + other VEGFR-TKIs, implying that TMPs may synergistically potentiate the anti-tumor effects of sorafenib/ lenvatinib against middle-advanced PLC. However, this result may be biased because there were few available studies on lenvatinib and regorafenib for metastasis. Further investigations are required to determine the effect of co-treatment of TMPs with different VEGFR-TKI regimens against various tumors.

In addition to improving ORR and DCR, the meta-analysis also revealed that TMPs + VEGFR-TKIs had a better effect on one-year OS than VEGFR-TKIs mono-treatment. Furthermore, all the studies that reported one-year OS included TMPs + sorafenib co-treatment. However, this result only supports TMPs + sorafenib co-treatment for improved one-year survival rate and cannot be extended to all other VEGFR-TKIs. This synergistic effect between TMPs and sorafenib may be due to the anti-tumor effect of TMPs [29], or its ability to reverse resistance to targeted drugs [70].

Although co-treatment of TMPs + VEGFR-TKIs significantly improved the primary outcome and one-year OS, the quality of life was not substantially altered based on dichotomous data, which might be because of the low quality of the evidence. The continuous data showed more encouraging trends with the co-treatment increasing the quality of life. This may be closely related to the lower incidence of ADRs within the TMPs + VEGFR-TKIs cohort.

Increased AFP level is a risk factor for PLC [73], and it is essentially associated with diagnosis, prognosis, and PLC monitoring [74]. Therefore, for this outcome, due to dataset heterogeneity, only qualitative analysis was performed. Here, ten trials showed that TMPs + VEGFR-TKIs reduced AFP, while three indicated no advantage for the co-therapy. In a retrospective cohort study, sorafenib combined with Fufang Banmao capsules, a type of TMP including *C*. *aromatica* and *S*. *barbata*, was found to decrease AFP levels in HCC patients [75]. Furthermore, icaritin, an extract from *Epimedium sagittatum* (Siebold & Zucc.) Maxim. [Berberidaceae], was observed to inhibit HCC metastasis by downregulating *AFP* gene expression [76]. This indicates that TMPs have the potential to reduce AFP and need further exploration.

The meta-analysis of moderate quality evidence suggested that, compared to control treatments, co-treatment with TMPs and VEGFR-TKIs may reduce the incidence of myelosuppression as an adverse drug reaction (ADR). Moreover, low quality evidence suggested that the co-treatment group had a lower incidence of liver dysfunction, hand-foot skin reactions, hypertension, gastrointestinal reactions, and proteinuria compared to the control group. The reduced ADRs might be related to the multi-component, -target, and -channel functions of TMPs. It has been revealed that some TMPs [77] and traditional medicines such as *Astragalus* [78] can treat proteinuria, some TMPs can control hypertension [79], and ginsenoside Rg3 can reduce myelosuppression [80]. Moreover, this study also found that TMPs + VEGFR-TKIs co-treatment does not increase the incidence of some common ADRs, such as rash and fatigue, compared with the control treatment. Therefore, the results of this investigation support that TMPs + VEGFR-TKIs co-therapy may be safe for middle-advanced PLC treatment.

### Implications for clinical practice

This review showed that TMPs combined that VEGFR-TKIs have better effectiveness and safety profile for treating patients with middle-advanced PLC than VEGFR-TKIs alone. This improved efficacy might be because of some traditional medicine’s ability to exert anti-tumor effects through multiple targets and pathways. Moreover, some traditional medicines can reverse multi-drug resistance and synergistically increase the anti-tumor effects of VEGFR-TKIs. Here, it was identified that the main TMP’s mechanisms included heat clearance, toxin removal, tonifying, and blood activation, liver soothing, depression relief, qi tonifying, and spleen fortifying. The most frequently used TMPs and traditional medicine in this review were Huaier granule and *A*. *macrocephala*, respectively. Based on this meta-analysis, the following traditional medicines are recommended for middle-advanced PLC treatment when combined with VEGFR-TKIs: *P*. *ginseng*, *T*. *robiniophila* Murr., *S*. *nigrum*, *R*. *glutinosa*, *A*. *sinensis*, *Citrus × aurantium*, *C*. *aromatica*, *P*. *persica*, *E*. *sinensis* walker, *S*. *barbata*, *S*. *s*. *mutilans*, *M*. *pentadactyla* Linnaeus, and *G*. *glabra* as they have a significant effect on ORR/DCR. Additionally, the following combinations of traditional medicines are recommended for use in conjunction with VEGFR-TKIs: *E*. *sinensis* Walker + *S*. *nigrum* + *R*. *glutinosa*, *C*. *aromatica* + *P*. *ternate* + *Citrus × aurantium*, and *S*. *barbata* + *S*. *s*. *mutilans*. The extracts and compounds of these 12 traditional medicines have potential *in vivo* and *in vitro* therapeutic effects on PLC. Their pharmacological actions and mechanisms are summarized in S5 Table within S5 File.

### Advantages and limitations of the review

In recent years, several systematic reviews of TMPs and systematic treatments of malignant tumors have been published, which suggest the potential anti-tumor effects of TMPs. Although previous review topics are similar, this review is different in several ways. 1) Methodologically, this research assessed evidence quality using GRADE criteria, a recognized standard procedure. 2) The sources of heterogeneity with sub-cohort analysis were also assessed. 3) The PRISMA guidelines were followed for meta-analysis studies [81]. Wang *et al*. reviewed the effect of TMPs + TACE co-treatment compared to TACE mono-treatment in PLC patients and revealed that the co-treatment could improve the effective rate and reduce serum VEGF levels [82]. However, this study differs from the current acquired data due to the treatment evaluation. Xun *et al*. reviewed 12 studies to evaluate the effect of TMPs + sorafenib in PLC patients and included all types of VEGFR-TKIs [83]. Although the treatment strategies are similar, this study specifically focused on the middle and advanced stages of PLC rather than the whole PLC. The results of this and Xun’s study were consistent for tumor response, quality-of-life, and a part of ADRs. However, in this study, gastrointestinal reactions, rash, and fatigue, the co-treatment cohort displayed no advantage over the control cohort. These differences might be because here, each type of ADR was evaluated separately. This work may be the first to probe the effectiveness and safety profiles for TMPs when in combination with VEGFR-TKIs as middle-advanced PLC therapy.

However, there are several limitations in this study. 1) Only English and Chinese language databases were surveyed, therefore, omissions may exist. All the included studies were sourced from China because of the main location for Chinese medicine research, which may have regional and ethnic influences. Further research is thus required to extend the current findings to other populations and regions. 2) Only 13 studies [44, 46–48, 50, 53–57, 61, 62, 64] reported random sequence generation, and none followed the blinding method, which may have introduced potential selection and detection biases. Therefore, the GRADE rating for the evidence quality of these data might be low or very low. However, the sensitivity analysis showed that the outcomes were reliable. The data will be constantly updated with the publication of new high-quality studies. 3) Only 8 studies [43, 44, 47, 58, 60, 65–67] reported the long-term survival in PLC patients, thus, warrants further research. 4) Although all studies reported quality control, only 8 studies [45, 50, 51, 53, 56, 58, 62, 65] included chemical analysis; the active ingredients of TMPs in other studies were unclear and required clarification from the drug manufacturers or research studies, which may cause bias. In addition, sensitivity and sub-cohort analyses were carried out to mitigate the effect of heterogeneity and the resulting bias for specific traditional medicine within TMPs. 5) The majority of the studies reviewed within this investigation did not adhere stringently to reporting standards for CONSORT Extension for Chinese Herbal Medicine Formulas [84]. 6) Using funnel plots, Begg’s test, and Egger’s test, we identified potential publication bias in DCR, hypertension, and hand-foot skin reactions. This may be attributable to publication bias favoring positive results, wherein studies with significant findings are more likely to be published. Additionally, some of the included studies had small sample sizes, which, if they happened to demonstrate larger effects, may have been more readily published, even though these effects may not be broadly representative. This can lead to potential publication bias. Furthermore, the literature search was restricted to studies published in English and Chinese, which may have contributed to bias as well. We recommend that future research efforts include pre-registered studies and encourage both researchers and journals to publish studies with non-significant findings to mitigate the impact of publication bias.

### Areas for future research

Several areas in this field warrant research in the future. First, it will be worth exploring the mechanism of traditional medicines for the synergistic potentiation of VEGFR-TKIs. Second, the effect of traditional medicine in combinations such as *C*. *aromatica* + *P*. *ternata* + *Citrus × aurantium* should be evaluated. Furthermore, the mechanism of action of traditional medicines can be assessed by isolating and purifying the monomers to identify their active ingredients, such as RG-3 extracted from *P*. *ginseng* C.A.Mey. [Araliaceae]. Third, since only eight trials reported one-year OS, more studies with long-term follow-up will significantly improve our understanding of the prolonged benefit of TMPs and their combinatorial uses. Fourth, ADR incidence was commonly reported; however, for therapeutic decision making more information should be recorded. Therefore, it is recommended to use standardized evaluating tools including NCI-CTCAE (National Cancer Institute-Common Toxicity Criteria for Adverse Events) for enhanced identification of ADR extent. Fifth, for clinical application and comprehensive understanding of this therapy, well-designed and high-quality clinical investigations are required. Thus, researchers should follow established good practices including the CONSORT Extension for Chinese Herbal Medicine Formulas guideline [84] for validating the effectiveness and safety profile of combinatory therapies of TMPs and VEGFR-TKIs for PLC. Sixth, because of the unclear TMP composition, the investigation of clinical outcomes is restricted. Future studies must preferably include quality control and chemical analysis of TMPs.

## Conclusions

This investigation indicated that TMPs + VEGFR-TKIs co-treatment has enhanced efficacy and reduced risk of ADRs, for middle-advance PLC patients. Furthermore, only the “TMPs + sorafenib” combination improved the one-year survival rate in long-term prognosis. In addition, TMPs + lenvatinib co-treatment has better ORR than the combination of TMPs with other VEGFR-TKIs. When it comes to DCR, TMPs + sorafenib co-treatment was better. Moreover, it was indicated that TMPs can reduce AFP and need further exploration. Based on the meta-analysis, it was recommended that for middle-advanced PLC treatment, the following traditional medicines should be combined with VEGFR-TKIs: *P*. *ginseng*, *T*. *robiniophila* Murr., *S*. *nigrum*, *R*. *glutinosa*, *A*. *sinensis*, *Citrus × aurantium*, *C*. *aromatica*, *P*. *persica*, *E*. *sinensis* walker, *S*. *barbata*, *S*. *s*. *mutilans*, *M*. *pentadactyla* Linnaeus, and *G*. *glabra*, as they have a significant effect on ORR/DCR. Additionally, following combinations of traditional medicines with VEGFR-TKIs were also recommended: *E*. *sinensis* Walker + *S*. *nigrum* + *R*. *glutinosa*; *C*. *aromatica* + *P*. *ternate* + *Citrus × aurantium*; *S*. *barbata* + *S*. *s*. *mutilans*. However, further verification is required with well-designed and high-quality clinical trials adhering to CONSORT Extension for Chinese Herbal Medicine Formulas guidelines.

## Supporting information

S1 File PRISMA checklist. The PRISMA checklist is available in S1 File.
(DOCX)

S2 File Detailed search strategy. The detailed search strategy is available in S2 File.
(DOCX)

S3 File Literature list. (XLSX)

S4 File Risk of bias assessment. (XLSX)

S5 File Attached figures and table. The S1–S10 Figs and S1-S5 Tables are available in S5 File.
(DOCX)

## Abbreviations

ADRs: adverse drug reactions
AFP: alpha fetoprotein
ALT: alanine aminotransferase
CFDA: China Food and Drug Administration
CI: confidence intervals
CR: complete response
CRC: colorectal cancer cells
CSCO: Chinese Society of Clinical Oncology
DCR: disease control rate
FDA: Food and Drug Administration
FEM: fixed-effects model
GRADE: Grading of Recommendations Assessment Development and Evaluation criteria
HCC: Hepatocellular Carcinoma
IPC: intraperitoneal chemotherapy
KPS: Karnofsky Performance Status
MA: malignant ascites
MD: mean difference
MDR: multidrug resistance
MT: matrine
MVD: micro vessel density
NCI-CTCAE: the national Cancer Institute-Common Toxicity Criteria for Adverse Events
ORR: objective response rate
OS: overall survival
PC: pancreatic cancer
PD: progressive disease
PLC: Primary liver cancer
PR: partial response
QoL: quality of life
RCTs: randomized controlled trials
REM: random-effects model
RR: risk ratio
SD: stable disease
SM: statistical method
SMD: standardized mean difference
TACE: transcatheter arterial chemoembolization
TKIs: targeted tyrosine kinase inhibitors
TMPs: Traditional medicine preparations
VEGFR: vascular endothelial growth factor receptor

## References

[1] Sung H, Ferlay J, Siegel RL, Laversanne M, Soerjomataram I, Jemal A, et al. Global Cancer Statistics 2020: GLOBOCAN Estimates of Incidence and Mortality Worldwide for 36 Cancers in 185 Countries. CA Cancer J Clin. 2021;71(3):209–49. doi: 10.3322/caac.21660 33538338

[2] Dakowicz D, Zajkowska M, Mroczko B. Relationship between VEGF Family Members, Their Receptors and Cell Death in the Neoplastic Transformation of Colorectal Cancer. Int J Mol Sci. 2022;23(6). doi: 10.3390/ijms23063375 35328794 PMC8952321

[3] Ferrara N, Gerber HP, LeCouter J. The biology of VEGF and its receptors. Nat Med. 2003;9(6):669–76. doi: 10.1038/nm0603-669 12778165

[4] Liu G, Chen T, Ding Z, Wang Y, Wei Y, Wei X. Inhibition of FGF-FGFR and VEGF-VEGFR signalling in cancer treatment. Cell Prolif. 2021;54(4):e13009. doi: 10.1111/cpr.13009 33655556 PMC8016646

[5] Kim KJ, Li B, Winer J, Armanini M, Gillett N, Phillips HS, et al. Inhibition of vascular endothelial growth factor-induced angiogenesis suppresses tumour growth in vivo. Nature. 1993;362(6423):841–4. doi: 10.1038/362841a0 7683111

[6] Ferrara N. Vascular endothelial growth factor: basic science and clinical progress. Endocr Rev. 2004;25(4):581–611. doi: 10.1210/er.2003-0027 15294883

[7] Bruix J, Qin S, Merle P, Granito A, Huang YH, Bodoky G, et al. Regorafenib for patients with hepatocellular carcinoma who progressed on sorafenib treatment (RESORCE): a randomised, double-blind, placebo-controlled, phase 3 trial. Lancet. 2017;389(10064):56–66. doi: 10.1016/S0140-6736(16)32453-9 27932229

[8] Cheng AL, Kang YK, Chen Z, Tsao CJ, Qin S, Kim JS, et al. Efficacy and safety of sorafenib in patients in the Asia-Pacific region with advanced hepatocellular carcinoma: a phase III randomised, double-blind, placebo-controlled trial. Lancet Oncol. 2009;10(1):25–34. doi: 10.1016/S1470-2045(08)70285-7 19095497

[9] Kudo M, Finn RS, Qin S, Han KH, Ikeda K, Piscaglia F, et al. Lenvatinib versus sorafenib in first-line treatment of patients with unresectable hepatocellular carcinoma: a randomised phase 3 non-inferiority trial. Lancet. 2018;391(10126):1163–73. doi: 10.1016/S0140-6736(18)30207-1 29433850

[10] Llovet JM, Ricci S, Mazzaferro V, Hilgard P, Gane E, Blanc JF, et al. Sorafenib in advanced hepatocellular carcinoma. N Engl J Med. 2008;359(4):378–90. doi: 10.1056/NEJMoa0708857 18650514

[11] Qin S, Bi F, Gu S, Bai Y, Chen Z, Wang Z, et al. Donafenib Versus Sorafenib in First-Line Treatment of Unresectable or Metastatic Hepatocellular Carcinoma: A Randomized, Open-Label, Parallel-Controlled Phase II-III Trial. J Clin Oncol. 2021;39(27):3002–11. doi: 10.1200/JCO.21.00163 34185551 PMC8445562

[12] Qin S, Li Q, Gu S, Chen X, Lin L, Wang Z, et al. Apatinib as second-line or later therapy in patients with advanced hepatocellular carcinoma (AHELP): a multicentre, double-blind, randomised, placebo-controlled, phase 3 trial. Lancet Gastroenterol Hepatol. 2021;6(7):559–68. doi: 10.1016/S2468-1253(21)00109-6 33971141

[13] Benson AB, D’Angelica MI, Abbott DE, Anaya DA, Anders, Are C, et al. Hepatobiliary Cancers, Version 2.2021, NCCN Clinical Practice Guidelines in Oncology. J Natl Compr Canc Netw. 2021;19(5):541–65. doi: 10.6004/jnccn.2021.0022 34030131

[14] Li Y, Gao ZH, Qu XJ. The adverse effects of sorafenib in patients with advanced cancers. Basic & clinical pharmacology & toxicology. 2015;116(3):216–21. doi: 10.1111/bcpt.12365 25495944

[15] Santoni M, Rizzo A, Kucharz J, Mollica V, Rosellini M, Marchetti A, et al. Complete remissions following immunotherapy or immuno-oncology combinations in cancer patients: the MOUSEION-03 meta-analysis. Cancer Immunol Immunother. 2023;72(6):1365–79. doi: 10.1007/s00262-022-03349-4 36633661 PMC10992808

[16] Santoni M, Rizzo A, Mollica V, Matrana MR, Rosellini M, Faloppi L, et al. The impact of gender on The efficacy of immune checkpoint inhibitors in cancer patients: The MOUSEION-01 study. Crit Rev Oncol Hematol. 2022;170:103596. doi: 10.1016/j.critrevonc.2022.103596 35031442

[17] Conforti F, Pala L, Bagnardi V, De Pas T, Martinetti M, Viale G, et al. Cancer immunotherapy efficacy and patients’ sex: a systematic review and meta-analysis. Lancet Oncol. 2018;19(6):737–46. doi: 10.1016/S1470-2045(18)30261-4 29778737

[18] Rizzo A, Ricci AD, Brandi G. Systemic adjuvant treatment in hepatocellular carcinoma: tempted to do something rather than nothing. Future Oncol. 2020;16(32):2587–9. doi: 10.2217/fon-2020-0669 32772560

[19] Abou-Alfa GK, Lau G, Kudo M, Chan SL, Kelley RK, Furuse J, et al. Tremelimumab plus Durvalumab in Unresectable Hepatocellular Carcinoma. NEJM Evid. 2022;1(8):EVIDoa2100070. doi: 10.1056/EVIDoa2100070 38319892

[20] Cheng AL, Qin S, Ikeda M, Galle PR, Ducreux M, Kim TY, et al. Updated efficacy and safety data from IMbrave150: Atezolizumab plus bevacizumab vs. sorafenib for unresectable hepatocellular carcinoma. J Hepatol. 2022;76(4):862–73. doi: 10.1016/j.jhep.2021.11.030 34902530

[21] Jin W, Han H, Zhou S, Wang Y, Dong T, Zhao C. Therapeutic efficacy of brucea javanica oil emulsion (BJOE) combined with transcatheter hepatic arterial chemoembolization (TACE) in patients with primary liver cancer. Int J Clin Exp Med. 2015;8(10):18954–62. 26770520 PMC4694420

[22] Liu Y, Li Y, Wang X, Huang Y, Zhang Q, Shi K, et al. Fufang Banmao Capsule, a Traditional Chinese Medicinal Formulation, Enhances the Survival of Patients with Hepatocellular Carcinoma and Vp3-4 Portal Vein Tumor Thrombosis Undergoing Supportive Treatment. J Altern Complement Med. 2020;26(10):956–65. doi: 10.1089/acm.2019.0334 32614605

[23] Zhang RR, Shao MY, Fu Y, Zhao RX, Wang JW, Li M, et al. [Systematic evaluation of Huaier Granules adjuvant treatment of primary liver cancer]. Zhongguo Zhong Yao Za Zhi. 2021;46(2):478–87. doi: 10.19540/j.cnki.cjcmm.20200716.502 33645137

[24] Jia S, Fu Y, Tao H. Trans-arterial chemoembolization combined with Jinlong capsule for advanced hepatocellular carcinoma: a PRISMA-compliant meta-analysis in a Chinese population. Pharmaceutical biology. 2020;58(1):771–84. doi: 10.1080/13880209.2020.1799040 32767901 PMC7470052

[25] Gao JL. [Prospective randomized controlled study on advanced primary hepatic cancer treated by ganfule prescription]. Zhongguo Zhong Yao Za Zhi. 2014;39(12):2367–9. 25244777

[26] Ma X, Jin S, Zhang Y, Wan L, Zhao Y, Zhou L. Inhibitory effects of nobiletin on hepatocellular carcinoma in vitro and in vivo. Phytother Res. 2014;28(4):560–7. doi: 10.1002/ptr.5024 23818450

[27] Feng S, Zhou H, Wu D, Zheng D, Qu B, Liu R, et al. Nobiletin and its derivatives overcome multidrug resistance (MDR) in cancer: total synthesis and discovery of potent MDR reversal agents. Acta Pharm Sin B. 2020;10(2):327–43. doi: 10.1016/j.apsb.2019.07.007 32082977 PMC7016283

[28] Shi MD, Liao YC, Shih YW, Tsai LY. Nobiletin attenuates metastasis via both ERK and PI3K/Akt pathways in HGF-treated liver cancer HepG2 cells. Phytomedicine: international journal of phytotherapy and phytopharmacology. 2013;20(8–9):743–52. doi: 10.1016/j.phymed.2013.02.004 23537747

[29] Shan L, Li Y, Jiang H, Tao Y, Qian Z, Li L, et al. Huaier Restrains Proliferative and Migratory Potential of Hepatocellular Carcinoma Cells Partially Through Decreased Yes-Associated Protein 1. J Cancer. 2017;8(19):4087–97. doi: 10.7150/jca.21018 29187885 PMC5706012

[30] Shan K, Wang Y, Hua H, Qin S, Yang A, Shao J. Ginsenoside Rg3 Combined with Oxaliplatin Inhibits the Proliferation and Promotes Apoptosis of Hepatocellular Carcinoma Cells via Downregulating PCNA and Cyclin D1. Biol Pharm Bull. 2019;42(6):900–5. doi: 10.1248/bpb.b18-00852 30930425

[31] Jiang JW, Chen XM, Chen XH, Zheng SS. Ginsenoside Rg3 inhibit hepatocellular carcinoma growth via intrinsic apoptotic pathway. World J Gastroenterol. 2011;17(31):3605–13. doi: 10.3748/wjg.v17.i31.3605 21987607 PMC3180017

[32] Ren Z, Chen X, Hong L, Zhao X, Cui G, Li A, et al. Nanoparticle Conjugation of Ginsenoside Rg3 Inhibits Hepatocellular Carcinoma Development and Metastasis. Small. 2020;16(2):e1905233. doi: 10.1002/smll.201905233 31814271

[33] Tao Y, Shan L, Xu X, Jiang H, Chen R, Qian Z, et al. Huaier Augmented the Chemotherapeutic Sensitivity of Oxaliplatin via Downregulation of YAP in Hepatocellular Carcinoma. J Cancer. 2018;9(21):3962–70. doi: 10.7150/jca.25909 30410600 PMC6218774

[34] Yang Y, Sun M, Yao W, Wang F, Li X, Wang W, et al. Compound kushen injection relieves tumor-associated macrophage-mediated immunosuppression through TNFR1 and sensitizes hepatocellular carcinoma to sorafenib. J Immunother Cancer. 2020;8(1). doi: 10.1136/jitc-2019-000317 32179631 PMC7073790

[35] Jing W, Shuo L, Yingru X, Min M, Runpeng Z, Jun X, et al. Artesunate promotes sensitivity to sorafenib in hepatocellular carcinoma. Biochemical and biophysical research communications. 2019;519(1):41–5. doi: 10.1016/j.bbrc.2019.08.115 31481232

[36] Kim YS, Lee YM, Oh TI, Shin DH, Kim GH, Kan SY, et al. Emodin Sensitizes Hepatocellular Carcinoma Cells to the Anti-Cancer Effect of Sorafenib through Suppression of Cholesterol Metabolism. Int J Mol Sci. 2018;19(10). 10.3390/ijms19103127 PMC6213641 30321984

[37] Zhao L, Wang Y, Liu Q. Catalpol inhibits cell proliferation, invasion and migration through regulating miR-22-3p/MTA3 signalling in hepatocellular carcinoma. Exp Mol Pathol. 2019;109:51–60. doi: 10.1016/j.yexmp.2019.104265 31145886

[38] Park JO, Lee SI, Song SY, Kim K, Kim WS, Jung CW, et al. Measuring response in solid tumors: comparison of RECIST and WHO response criteria. Jpn J Clin Oncol. 2003;33(10):533–7. doi: 10.1093/jjco/hyg093 14623923

[39] Miller AB, Hoogstraten B, Staquet M, Winkler A. Reporting results of cancer treatment. Cancer. 1981;47(1):207–14. doi: 10.1002/1097-0142(19810101)47:1&lt;207::aid-cncr2820470134&gt;3.0.co;2-6 7459811

[40] JPT H, J T, J C, M C, T L, MJ P, et al. Cochrane Handbook for Systematic Reviews of Interventions version 6.3 (updated February 2022) Cochrane, 20222022 [www.training.cochrane.org/handbook.

[41] Guyatt GH, Oxman AD, Vist GE, Kunz R, Falck-Ytter Y, Alonso-Coello P, et al. GRADE: an emerging consensus on rating quality of evidence and strength of recommendations. BMJ. 2008;336(7650):924–6. doi: 10.1136/bmj.39489.470347.AD 18436948 PMC2335261

[42] Chen M, May BH, Zhou IW, Xue CC, Zhang AL. Meta-Analysis of Oxaliplatin-Based Chemotherapy Combined With Traditional Medicines for Colorectal Cancer: Contributions of Specific Plants to Tumor Response. Integr Cancer Ther. 2016;15(1):40–59. doi: 10.1177/1534735415596424 26254190 PMC5736077

[43] Duan KN. Analysis of sorafenib combined with traditional Chinese medicine decoction in the treatment of advanced primary liver cancer. World Latest Medicine Information. 2018;18(A2):211–2.

[44] Fang HS. ShenTao RuanGan Fang combined sorafenib treatment of advanced HCC clinical study and the research of mechanism [phD Thesis]: Guangzhou University of Chinese Medicine; 2015.

[45] Feng LH, Chen YD, Zheng ZG, Gao YQ. Clinical observation of patients with primary liver cancer treated by sorafenib combined cinobufagin tablets. China Oncol. 2012;22(11):856–9.

[46] Gao YY, Shen JH, Jin XX, Meng XR, He SL. Clinical efficacy of Peiyuan Kangai Decoction with apatinib mesylate tablets for advanced primary liver cancer of spleen-deficiency blood stasis and how to impact on peripheral immune cells and tumor marker. Hebei Journal of Traditional Chinese Medicine. 2022;44(1):96–9.

[47] Han GM. Clinical observation on the treatment ofadvanced liver cancer with modified Chaihu Biejia decoction combined with sorafenib. Chinese Medicine Modern Distance Education of China. 2021;19(2):148–50.

[48] Jiang XQ, Wang LN. Efficacy of Chaihu Shugan Huayu decoction combined with lenvatinib in the treatment of primary liver cancer (Qi stagnation and blood obstruction syndrome). Chin J Integr Tradit West Med Liver Dis. 2022;32(5):462–4.

[49] Jin J, Lin H, Wu YW, Du JH. Toxicity, Prognostic Factors and Survival Analysis of Ganji Decoction Combined with Apatinib in the Treatment of Advanced Hepatocellular Carcinoma. J Liaoning Univ Tradit Chin Med. 2020;22(11):18–22.

[50] Jin Z, Xie YH, He C, Wu Q. Clinical observation of Huisheng oral liquid combined with lunvatinib in treating advanced liver cancer. China’s Naturopathy. 2022;30(24):120–3.

[51] Li DQ, Chen PX. Effect of Wuzhi tablets on preventing liver injury in patients with primary liver cancer caused by sorafenib. Guangdong Med J. 2018;39(20).

[52] Liu JP, Cao JG, Yuan CJ, ZHOU XJ, LU W, Liu L. Evaluation ofTherapeutic Effect ofQinghuo Tongluo Prescription Combined with Sorafenib on Liver Cancer Based on“Image Thinking”. J Hubei Univ Tradit Chin Med. 2018;20(05):22–5.

[53] Ma JR, Zhang K. Efficacy of Huazhirougan Granules on Regofinil Targeted Therapy of Middle and Advanced Liver Cancer Patients with Damp Heat Accumulation Type. J Clin Res. 2022;39(4).

[54] Ma YK, Fang SM, Chen L, Yang JZ, Yang XY, Shen TH, et al. Clinical efficacy of Jianpi Jiedu decoction combined with apatinib mesylate on advanced primary liver cancer. J Hebei Tradit Chin Med. 2018;40(11):1682–6.

[55] Qiao CX, Zhang XF, Cheng XF, Cai XP, Liu Q. Fuzheng Guben therapy improves quality of life in patients with hepatocellular carcinoma on targeted therapy. World Chin J Digestol. 2015(33):5383–7.

[56] Sun J, Zhang F. Effect of elemene injection combined with molecular targeting drugs on immunity and survival status of patients with liver cancer metastasis. Chin J Integr Tradit West Med Liver Dis. 2022;32(01):16–9.

[57] Sun Y. Effect of Jiawei Yiyi decoction combined with apatinib on the efficacy and quality of life of patients with advanced primary liver cancer. J Yunnan Tradit Chin Med. 2019;40(4).

[58] Tang YF, Zhu XJ, Huang LY, Zhang X, Zheng C, Gao YQ. Clinical study on Huaier Granules combined with sorafenib in treatment of advanced liver cancer. Drugs Clin. 2018;33(7):1732–5.

[59] Tu XL, Shi GJ, Zhang TS, Wang YS, Hu MY, Wu W, et al. Clinical observation of Jianpi Yanggan Jiedu decoction combined with lenvatinib in the treatment of advanced primary liver cancer. Chin J Tradit Med Sci Technol. 2021;28(5):781–2.

[60] Wang GT, Yang XW, Wang Q, Wang X. Curative effect of rosin combine with sorafenib in treatment of patients with moderate and advanced primary liver cancer. J Clin Hepatol. 2016;19(5).

[61] Wu YW. The study of clinical observation of Jianpi Rougan combined apatinib in treatment of advanced hepatocellular carcinoma [Master’s thesis]: Guangzhou University of Chinese Medicine; 2018.

[62] Yang CJ. Clinical Observation of Jiawei Xiaochaihu Decoction combined with sorafenib in the treatment of middle and advanced primary liver cancer [Master’s thesis]: Chengdu University of Chinese Medicine; 2021.

[63] Yu JF, Li ZP, Zhou XL, Li C, Liu Y, Zhang Y, et al. Effect of Fuling Sini decoction combined with sorafenib on advanced primary liver cancer. Acta Chin Med Pharmacol. 2021;49(6):76–80.

[64] Zhan LH. Clinical study of Chai Shao Tang combined with apatinib mesylate tablets in the treatment of TACE for primary liver cancer (Liver depression and spleen deficiency type) [Master’s thesis]: Guangxi University of Chinese Medicine; 2022.

[65] Zhang QH, Liang YH. Clinical Efficacy of Huaier Granule Combined with Sorafenib on Patients with Postoperative Recurrence of Primary Liver Cancer. The Practical Journal of Cancer. 2019;34(09):1560–2.

[66] Zhang Z, Gao WH, Wang YQ, Li KX, Zeng PH. Study on curative effect of Yiqi Huayu Jiedu decoction combined with sorafenib in the treatment of primary hepatocellular caicinoma. Shaanxi J Tradit Chin Med. 2019;40(3):322–4.

[67] Zhou FJ, Chang JY, Meng J, Zhang X, Wei XX, Yang LC. Clinical observation on the treatment of advanced hepatocellular carcinoma with Sanjia powder combined with sorafenib based on the theory of "master and guest". World Latest Medicine Information. 2020;20(30):121–2.

[68] Zhu X. An observation of the impact on Danzhi Xiaoyao Powder and Remvastinib in the therapy of advanced primary hepatocellular carcinomas of the liver depression and spleen deficiency types [Master’s thesis]2023.

[69] Hayashino Y, Noguchi Y, Fukui T. Systematic evaluation and comparison of statistical tests for publication bias. J Epidemiol. 2005;15(6):235–43.. doi: 10.2188/jea.15.235 16276033 PMC7904376

[70] Niu Y, Shan L, Gao H, Zhang C, Qian Z, Wang Z, et al. Huaier Suppresses the Hepatocellular Carcinoma Cell Cycle by Regulating Minichromosome Maintenance Proteins. Onco Targets Ther. 2020;13:12015–25. doi: 10.2147/OTT.S279723 33244243 PMC7685376

[71] Yang MY, Hung CH, Chang CH, Tseng TH, Wang CJ. Solanum nigrum Suppress Angiogenesis-Mediated Tumor Growth Through Inhibition of the AKT/mTOR Pathway. Am J Chin Med. 2016;44(6):1273–88. doi: 10.1142/S0192415X16500713 27627922

[72] El-Hanboshy SM, Helmy MW, Abd-Alhaseeb MM. Catalpol synergistically potentiates the anti-tumour effects of regorafenib against hepatocellular carcinoma via dual inhibition of PI3K/Akt/mTOR/NF-kappaB and VEGF/VEGFR2 signaling pathways. Molecular biology reports. 2021;48(11):7233–42. 34596810 10.1007/s11033-021-06715-0

[73] Forner A, Reig M, Bruix J. Hepatocellular carcinoma. Lancet. 2018;391(10127):1301–14. doi: 10.1016/S0140-6736(18)30010-2 29307467

[74] Trevisani F, Garuti F, Neri A. Alpha-fetoprotein for Diagnosis, Prognosis, and Transplant Selection. Semin Liver Dis. 2019;39(2):163–77. doi: 10.1055/s-0039-1677768 30849784

[75] Lai X, Wang A. Effects of Fufang Banmao Capsule Associated with Sorafenib on Liver Function, Immune Status, Quality of Life Improvement, and Survival in Patients with Advanced Hepatocellular Carcinoma: A Retrospective Cohort Study. Comput Intell Neurosci. 2022;2022:6336107. 36052044 10.1155/2022/6336107 PMC9427212

[76] Li H, Liu Y, Jiang W, Xue J, Cheng Y, Wang J, et al. Icaritin promotes apoptosis and inhibits proliferation by down-regulating AFP gene expression in hepatocellular carcinoma. BMC Cancer. 2021;21(1):318. doi: 10.1186/s12885-021-08043-9 33765973 PMC7992931

[77] Chen Y, Deng Y, Ni Z, Chen N, Chen X, Shi W, et al. Efficacy and safety of traditional chinese medicine (Shenqi particle) for patients with idiopathic membranous nephropathy: a multicenter randomized controlled clinical trial. Am J Kidney Dis. 2013;62(6):1068–76. doi: 10.1053/j.ajkd.2013.05.005 23810688

[78] Guo MF, Dai YJ, Gao JR, Chen PJ. Uncovering the Mechanism of Astragalus membranaceus in the Treatment of Diabetic Nephropathy Based on Network Pharmacology. J Diabetes Res. 2020;2020:5947304. doi: 10.1155/2020/5947304 32215271 PMC7079250

[79] Li J. Traditional Chinese Medicine in Treating Hypertension. Circ Cardiovasc Qual Outcomes. 2022;15(3):e008723. doi: 10.1161/CIRCOUTCOMES.121.008723 35105174

[80] Pan L, Zhang T, Cao H, Sun H, Liu G. Ginsenoside Rg3 for Chemotherapy-Induced Myelosuppression: A Meta-Analysis and Systematic Review. Front Pharmacol. 2020;11:649. doi: 10.3389/fphar.2020.00649 32477128 PMC7235324

[81] McInnes MDF, Moher D, Thombs BD, McGrath TA, Bossuyt PM, and the P-DTAG, et al. Preferred Reporting Items for a Systematic Review and Meta-analysis of Diagnostic Test Accuracy Studies: The PRISMA-DTA Statement. JAMA. 2018;319(4):388–96. doi: 10.1001/jama.2017.19163 29362800

[82] Wang C. Meta-analysis of the correlation between effect of Traditional Chinese medicine on hepatocellular carcinoma and VEGF And the experimental study on inhibiting angiogenesis of hepatocellular carcinoma by "anti-tumor prescription" [Master’s thesis]: Wannan Medical College; 2021.

[83] Xun YC, Mo CM, Jiang RY, Tang CJ, Hong XH, Rong Z. Meta-analysis of Traditional Chinese Medicine Combined with Sorafenib in Treatment of Primary Liver Cancer. World Journal of Integrated Traditional and Western Medicine. 2020;15(09):1597–602.

[84] Cheng CW, Wu TX, Shang HC, Li YP, Altman DG, Moher D, et al. CONSORT Extension for Chinese Herbal Medicine Formulas 2017: Recommendations, Explanation, and Elaboration. Ann Intern Med. 2017;167(2):112–21. doi: 10.7326/M16-2977 28654980

