# Supplementary material for: Combinatorial treatment with traditional medicinal preparations and VEGFR-tyrosine kinase inhibitors for middle-advanced primary liver cancer: A systematic review and meta-analysis
Source: PLoS One. 2024 Nov 22;19(11):e0313443. doi: 10.1371/journal.pone.0313443 (PMC11584121; doi:10.1371/journal.pone.0313443)
Supplement: S2 File — The detailed search strategy is available in S2 File. (DOCX) [file pone.0313443.s002.docx]

**Supplementary material 2. Detailed search strategy**

**Supplementary Table A: Search Strategy Used in PubMed 2024/4/12**

**Items found 16**

| No. | Search items | Items found |
| --- | --- | --- |
| #1 | ("neoplasms"[MeSH Terms] OR "carcinoma"[MeSH Terms] OR "cancer*"[Title/Abstract] OR "carcin*"[Title/Abstract] OR "neoplas*"[Title/Abstract] OR "tumo*"[Title/Abstract]) AND ("Carcinoma, Hepatocellular"[MeSH Terms] OR "liver"[MeSH Terms] OR "liver"[Title/Abstract]) | 346601 |
| #2 | "complementary therapies"[MeSH Terms] OR "drugs, Chinese herbal"[MeSH Terms] OR "herbal medicine"[MeSH Terms] OR "medicine, traditional"[MeSH Terms] OR "medicine, east asian traditional"[MeSH Terms] OR "plant extracts"[MeSH Terms] OR "plants, medicinal"[MeSH Terms] OR "phytotherapy"[MeSH Terms] OR "alternative medicine"[Title/Abstract] OR "complementary therap*"[Title/Abstract] OR "Chinese herba*"[Title/Abstract] OR "Chinese medicine"[Title/Abstract] OR "herb*"[Title/Abstract] OR "herbalism"[Title/Abstract] OR "herbal medicine"[Title/Abstract] OR "herbal drugs"[Title/Abstract] OR "plant extract*"[Title/Abstract] OR "medicinal plant*"[Title/Abstract] OR "phytotherapy*"[Title/Abstract] OR "phytopharmaceutic*"[Title/Abstract] OR "traditional medicine"[Title/Abstract] OR "oriental medicine"[Title/Abstract] OR "zhong yi xue"[Title/Abstract] | 606071 |
| #3 | ("randomized controlled trial"[Publication Type] OR "controlled clinical trial"[Publication Type] OR "randomized"[Title/Abstract] OR "placebo"[Title/Abstract] OR "drug therapy"[MeSH Terms] OR "randomly"[Title/Abstract] OR "trial"[Title/Abstract] OR "groups"[Title/Abstract]) NOT ("animals"[MeSH Terms] NOT "humans"[MeSH Terms]) | 4318120 |
| #4 | "Sorafenib"[MeSH Terms] OR "Receptors, Vascular Endothelial Growth Factor"[MeSH Terms] OR "lenvatinib"[Title/Abstract] OR "apatinib"[Title/Abstract] OR "regorafenib"[Title/Abstract] OR "target therapy"[Title/Abstract] | 33053 |
| #5 | #1 and #2 and #3 and #4 | 17 |

**Supplementary Table B. Search Strategy Used in EMBASE 2024/4/12**

**Items found 29**

| #45. | #11 AND #36 AND #37 AND #44 | 29 |
| --- | --- | --- |
| #44. | #38 OR #39 OR #40 OR #41 OR #42 OR #43 | 64734 |
| #43. | 'target therapy':ti,ab,kw | 6085 |
| #42. | 'regorafenib':ti,ab,kw | 3899 |
| #41. | 'apatinib':ti,ab,kw | 2031 |
| #40. | 'lenvatinib':ti,ab,kw | 4336 |
| #39. | 'receptors, vascular endothelial growth factor'/exp | 13582 |
| #38. | 'sorafenib'/exp | 39677 |
| #37. | random* | 2330204 |
| #36. | #12 OR #13 OR #14 OR #15 OR #16 OR #17 OR #18 OR #19 OR #20 OR #21 OR #22 OR #23 OR #24 OR #25 OR #26 OR #27 OR #28 OR #29 OR #30 OR #31 OR #32 OR #33 OR #34 OR #35 | 803854 |
| #35. | 'zhong yi xue':ti,ab,kw | 2 |
| #34. | 'oriental medicine':ti,ab,kw | 1749 |
| #33. | 'traditional medicine':ti,ab,kw | 25206 |
| #32. | 'phytopharmaceutic*':ti,ab,kw | 1397 |
| #31. | 'phytotherapy*':ti,ab,kw | 4838 |
| #30. | 'medicinal plant*':ti,ab,kw | 48375 |
| #29. | 'plant extract*':ti,ab,kw | 24622 |
| #28. | 'herbal drugs':ti,ab,kw | 3630 |
| #27. | 'herbal medicine':ti,ab,kw | 23930 |
| #26. | herbalism:ti,ab,kw | 291 |
| #25. | herb*:ti,ab,kw | 186014 |
| #24. | 'chinese medicine*':ti,ab,kw | 60332 |
| #23. | 'chinese herba*':ti,ab,kw | 13339 |
| #22. | 'complementary therap*':ti,ab,kw | 8110 |
| #21. | 'alternative medicine':ti,ab,kw | 17281 |
| #20. | 'phytotherapy'/exp | 19272 |
| #19. | 'medicinal plant'/exp | 323378 |
| #18. | 'plant extract'/exp | 290308 |
| #17. | 'chinese medicine'/exp | 79411 |
| #16. | 'oriental medicine'/exp | 3154 |
| #15. | 'traditional medicine'/exp | 146949 |
| #14. | 'herbal medicine'/exp | 30315 |
| #13. | 'herbaceous agent'/exp | 62192 |
| #12. | 'alternative medicine'/exp | 79794 |
| #11. | #7 AND #10 | 345073 |
| #10. | #8 OR #9 | 922575 |
| #9. | 'liver'/exp | 741807 |
| #8. | 'carcinoma, hepatocellular'/exp | 219269 |
| #7. | #1 OR #2 OR #3 OR #4 OR #5 OR #6 | 7344627 |
| #6. | tumo*:ti,ab,kw | 2932907 |
| #5. | neoplas*:ti,ab,kw | 569988 |
| #4. | carcin*:ti,ab,kw | 1311473 |
| #3. | cancer*:ti,ab,kw | 3406538 |
| #2. | 'carcinoma'/exp | 1594057 |
| #1. | 'neoplasm'/exp | 6263617 |

**Supplementary Table C. Search Strategy Used in** **Cochrane 2024/4/12**

**Items found 3**

| **No.** | **Search items** | **Items found** |
| --- | --- | --- |
| **Search Terms to Pancreatic Cancer:** | | |
| #1 | MeSH descriptor: [Neoplasms] explode all trees | 124365 |
| #2 | MeSH descriptor: [Carcinoma] explode all trees | 20188 |
| #3 | MeSH descriptor: [Carcinoma, Hepatocellular] explode all trees | 2715 |
| #4 | (cancer*):ti,ab,kw | 208641 |
| #5 | (carcin*):ti,ab,kw | 54832 |
| #6 | (neoplas*):ti,ab,kw | 123643 |
| #7 | (tumo*):ti,ab,kw | 94582 |
| #8 | #1 OR #2 OR #3 OR #4 OR #5 OR #6 OR #7 | 282263 |
| #9 | MeSH descriptor: [Liver] explode all trees | 4633 |
| #10 | #3 OR #9 | 7205 |
| #11 | #8 AND #10 | 3365 |
| **Search Terms to Interventions:** | | |
| #12 | MeSH descriptor: [Complementary Therapies] explode all trees | 28993 |
| #13 | MeSH descriptor: [Drugs, Chinese Herbal] explode all trees | 4603 |
| #14 | MeSH descriptor: [Herbal Medicine] explode all trees | 100 |
| #15 | MeSH descriptor: [Medicine, Traditional] explode all trees | 2240 |
| #16 | MeSH descriptor: [Medicine, East Asian Traditional] explode all trees | 1926 |
| #17 | MeSH descriptor: [Plant Extracts] explode all trees | 11003 |
| #18 | MeSH descriptor: [Plants, Medicinal] explode all trees | 1149 |
| #19 | MeSH descriptor: [Phytotherapy] explode all trees | 5033 |
| #20 | (alternative medicine):ti,ab,kw | 4866 |
| #21 | (complementary therap*):ti,ab,kw | 6137 |
| #22 | (chinese herba*):ti,ab,kw | 3653 |
| #23 | (chinese medicine):ti,ab,kw | 16356 |
| #24 | (herb*):ti,ab,kw | 14850 |
| #25 | (herbalism):ti,ab,kw | 9 |
| #26 | (herbal medicine):ti,ab,kw | 5803 |
| #27 | (herbal drugs):ti,ab,kw | 6017 |
| #28 | (plant extract*):ti,ab,kw | 8047 |
| #29 | (Medicinal Plant*):ti,ab,kw | 2560 |
| #30 | (Phytotherapy*):ti,ab,kw | 4914 |
| #31 | (phytopharmaceutic*):ti,ab,kw | 45 |
| #32 | (traditional medicine):ti,ab,kw | 14301 |
| #33 | (oriental medicine):ti,ab,kw | 113 |
| #34 | (zhong yi xue):ti,ab,kw | 2 |
| #35 | #12 OR #13 OR #14 OR #15 OR #16 OR #17 OR #18 OR #19 OR #20 OR #21 OR #22 OR #23 OR #24 OR #25 OR #26 OR #27 OR #28 OR #29 OR #30 OR #31 OR #32 OR #33 OR #34 | 67373 |
| #36 | MeSH descriptor: [sorafenib] explode all trees | 713 |
| #37 | MeSH descriptor: ['receptors, vascular endothelial growth factor'] explode all trees | 705 |
| #38 | (lenvatinib):ti,ab,kw | 669 |
| #39 | (apatinib):ti,ab,kw | 508 |
| #40 | (regorafenib):ti,ab,kw | 690 |
| #41 | (target therapy):ti,ab,kw | 35649 |
| #42 | #36 OR #37 OR #38 OR #39 OR #40 OR #41 | 38634 |
| #43 | #11 AND #35 AND #42 | 3 |

**Supplementary Table D: Search Strategy Used in CBM 2024/4/12**

**Items found 68**

(("肿瘤" [不加权:扩展]) OR ("癌" [不加权:扩展]) OR ("肿瘤"[摘要:智能]) OR ("癌"[摘要:智能])) AND (("肝" [不加权:扩展]) OR ("肝"[摘要:智能])) AND (("中草药"[不加权:扩展]) OR ("植物, 药用"[不加权:扩展]) OR ("医学, 中国传统"[不加权:扩展]) OR ("医学, 东亚传统"[不加权:扩展]) OR ("医学, 朝鲜传统"[不加权:扩展]) OR ("植物提取物"[不加权:扩展]) OR ("中医学"[不加权:扩展]) OR ("中药"[不加权:扩展]) OR ("补充替代医学"[摘要:智能]) OR ("中成药"[摘要:智能]) OR ("中草药"[摘要:智能]) OR ("汤药"[摘要:智能]) OR ("中医"[摘要:智能]) OR ("草药"[摘要:智能]) OR ("植物提取物"[摘要:智能]) OR ("传统医学"[摘要:智能]) OR ("中药"[摘要:智能])) AND ("随机" [摘要:智能]) AND (("索拉菲尼" [不加权:扩展]) OR ("仑伐替尼" [不加权:扩展]) OR ("瑞格菲尼" [不加权:扩展]) OR ("阿帕替尼" [不加权:扩展]) OR ("卡博替尼" [不加权:扩展]) OR ("靶向" [不加权:扩展]) OR ("VEGF" [不加权:扩展]))

**Supplementary E: Search Strategy Used in CNKI 2024/4/12**

**Items found 540**

(SU = ('肝癌' + '肝恶性肿瘤' + '原发性肝细胞癌' + '肝脏恶性肿瘤' + '肝肿瘤' + '肝细胞癌' + '肝内胆管癌') AND TKA=('肝癌' + '肝恶性肿瘤' + '原发性肝细胞癌' + '肝脏恶性肿瘤' + '肝肿瘤' + '肝细胞癌' + '肝内胆管癌')) AND (SU = '中草药' OR SU = '中成药' OR SU = '植物药' OR SU = '药用植物' OR SU = '传统医学' OR SU = '中医' OR SU = '植物提取物' OR SU = '中药' OR TKA = '补充替代医学' OR TKA = '中成药' OR TKA = '中草药' OR TKA = '汤药' OR TKA = '中医' OR TKA = '草药' OR TKA = '植物提取物' OR TKA = '传统医学' TKA = '中药' OR TKA = '药用植物' OR TI = '方' OR TI = '汤' OR TI = '丸' OR TI = '法') AND (SU=('多纳非尼' + '靶向' + '仑伐替尼' + '索拉菲尼' + '阿帕替尼' + '瑞格菲尼' + 'VEGF' + ‘索拉非尼’ + ‘卡博替尼’) OR TKA = ('多纳非尼' + '靶向' + '仑伐替尼' + '索拉菲尼' + '阿帕替尼' + '瑞格菲尼' + 'VEGF' + ‘索拉非尼’ + ‘卡博替尼’)) AND TKA=(‘随机’ + ‘RCT’ + '临床观察' + '疗效' + '体会' + '应用' + '评价' + '临床效果' + '临床研究' + '随机对照试验')

**Supplementary Table F: Search Strategy Used in WangFang 2024/4/12**

**Items found 264**

(主题:("肝癌" or "肝恶性肿瘤" or "原发性肝细胞癌" or "肝脏恶性肿瘤" or "肝肿瘤" or "肝细胞癌" or "肝内胆管癌") and 题名或关键词:("肝癌" or "肝恶性肿瘤" or "原发性肝细胞癌" or "肝脏恶性肿瘤" or "肝肿瘤" or "肝细胞癌" or "肝内胆管癌")) and (主题:("中草药" OR "中成药" OR "植物药" OR "药用植物" OR "传统医学" OR "中医" OR "植物提取物" OR "中药") or 题名或关键词:("补充替代医学" OR "中成药" OR "中草药" OR "汤药" OR "中医" OR "草药" OR "植物提取物" OR "传统医学" "中药" OR "药用植物") or 题名:("方" OR "汤" OR "丸" OR "法")) and 主题:("靶向" or "仑伐替尼" or "索拉菲尼" or "阿帕替尼" or "瑞格菲尼" or "VEGF" or "索拉非尼" or "卡博替尼") and 题名或关键词:("随机" or "RCT" or "临床观察" or "疗效" or "体会" or "应用" or "评价" or "临床效果" or "临床研究" or "随机对照试验")

**Supplementary G: Search Strategy Used in VIP 2024/4/12**

**Items found 1621**

(U = ("肝癌" or "肝恶性肿瘤" or "原发性肝细胞癌" or "肝脏恶性肿瘤" or "肝肿瘤" or "肝细胞癌" or "肝内胆管癌")) and (U = ("中草药" OR "中成药" OR "植物药" OR "药用植物" OR "传统医学" OR "中医" OR "植物提取物" OR "中药") or T = ("方" OR "汤" OR "丸" OR "法")) and U = ("靶向" or "仑伐替尼" or "索拉菲尼" or "阿帕替尼" or "瑞格菲尼" or "VEGF" or "索拉非尼" or "卡博替尼") and U = ("随机" or "RCT" or "临床观察" or "疗效" or "体会" or "应用" or "评价" or "临床效果" or "临床研究" or "随机对照试验")

**Supplementary Table H. Search Strategy Used in Clinicaltrials 2024/4/12**

**Items found 13**

Condition or disease: liver cancer |Other terms: "complementary therapies" OR "drugs, Chinese herbal" OR "herbal medicine" OR "medicine, traditional" OR "medicine, east asian traditional" OR "plant extracts" OR "plants, medicinal" OR "phytotherapy" OR "alternative medicine"

**Supplementary I: Search Strategy Used in Chinese Clinical Trial Registry 2024/4/12**

**Items found 2**

肝癌+靶向

26---2

肝癌+索拉非尼

16---0

肝癌+仑伐替尼

32--0

肝癌+阿帕替尼

56-0

肝癌+瑞格菲尼

0

肝癌+卡博替尼

0

肝癌+vegf

3-0

**Supplementary J: Search Strategy Used in TRIP medical database 2024/4/12**

**Items found 9**

**Population：**

("neoplasms" OR "carcinoma" OR "cancer*" OR "carcin*" OR "neoplas*" OR "tumo*") AND ("Carcinoma, Hepatocellular" OR "liver")

**Intervention**

"complementary therapies" OR "drugs, Chinese herbal" OR "herbal medicine" OR "medicine, traditional" OR "medicine, east asian traditional" OR "plant extracts" OR "plants, medicinal" OR "phytotherapy" OR "alternative medicine" OR "complementary therap*" OR "Chinese herba*" OR "Chinese medicine" OR "herb*" OR "herbalism" OR "herbal medicine" OR "herbal drugs" OR "plant extract*" OR "medicinal plant*" OR "phytotherapy*" OR "phytopharmaceutic*" OR "traditional medicine" OR "oriental medicine" OR "zhong yi xue"

**Comparison**

"Sorafenib" OR "Receptors, Vascular Endothelial Growth Factor" OR "lenvatinib" OR "apatinib" OR "regorafenib" OR "target therapy"

**Supplementary K: Search Strategy Used in Latin American and Caribbean Health Sciences Literature (LILACS) 2024/4/12**

**Items found 0**

**("neoplasms" OR "carcinoma" OR "cancer*" OR "carcin*" OR "neoplas*" OR "tumo*") AND ("Carcinoma, Hepatocellular" OR "liver") [Words] and "complementary therapies" OR "drugs, Chinese herbal" OR "herbal medicine" OR "medicine, traditional" OR "medicine, east asian traditional" OR "plant extracts" OR "plants, medicinal" OR "phytotherapy" OR "alternative medicine" OR "complementary therap*" OR "Chinese herba*" OR "Chinese medicine" OR "herb*" OR "herbalism" OR "herbal medicine" OR "herbal drugs" OR "plant extract*" OR "medicinal plant*" OR "phytotherapy*" OR "phytopharmaceutic*" OR "traditional medicine" OR "oriental medicine" OR "zhong yi xue" [Words] and "Sorafenib" OR "Receptors, Vascular Endothelial Growth Factor" OR "lenvatinib" OR "apatinib" OR "regorafenib" OR "target therapy" [Words]**

**Supplementary L: Search Strategy Used in Alt HealthWatch 2024/4/12**

**Items found 5**

| No. | Search items | Items found |
| --- | --- | --- |
| #1 | ("neoplasms" OR "carcinoma" OR "cancer*" OR "carcin*" OR "neoplas*" OR "tumo*") AND ("Carcinoma, Hepatocellular" OR "liver") | 1397 |
| #2 | "complementary therapies" OR "drugs, Chinese herbal" OR "herbal medicine" OR "medicine, traditional" OR "medicine, east asian traditional" OR "plant extracts" OR "plants, medicinal" OR "phytotherapy" OR "alternative medicine" OR "complementary therap*" OR "Chinese herba*" OR "Chinese medicine" OR "herb*" OR "herbalism" OR "herbal medicine" OR "herbal drugs" OR "plant extract*" OR "medicinal plant*" OR "phytotherapy*" OR "phytopharmaceutic*" OR "traditional medicine" OR "oriental medicine" OR "zhong yi xue" | 59970 |
| #3 | "Sorafenib" OR "Receptors, Vascular Endothelial Growth Factor" OR "lenvatinib" OR "apatinib" OR "regorafenib" OR "target therapy" | 46 |
| #4 | ("randomized controlled trial" OR "controlled clinical trial" OR "randomized" OR "placebo" OR "drug therapy" OR "randomly" OR "trial"[Title/Abstract] OR "groups") NOT ("animals" NOT "humans") | 16037 |
| #5 | #1 and #2 and #3 and #4 | 5 |
