# Supplementary material for: Combinatorial treatment with traditional medicinal preparations and VEGFR-tyrosine kinase inhibitors for middle-advanced primary liver cancer: A systematic review and meta-analysis
Source: PLoS One. 2024 Nov 22;19(11):e0313443. doi: 10.1371/journal.pone.0313443 (PMC11584121; doi:10.1371/journal.pone.0313443)
Supplement: S5 File — The S1–S10 Figs and S1-S5 Tables are available in S5 File. (DOCX) [file pone.0313443.s005.docx]

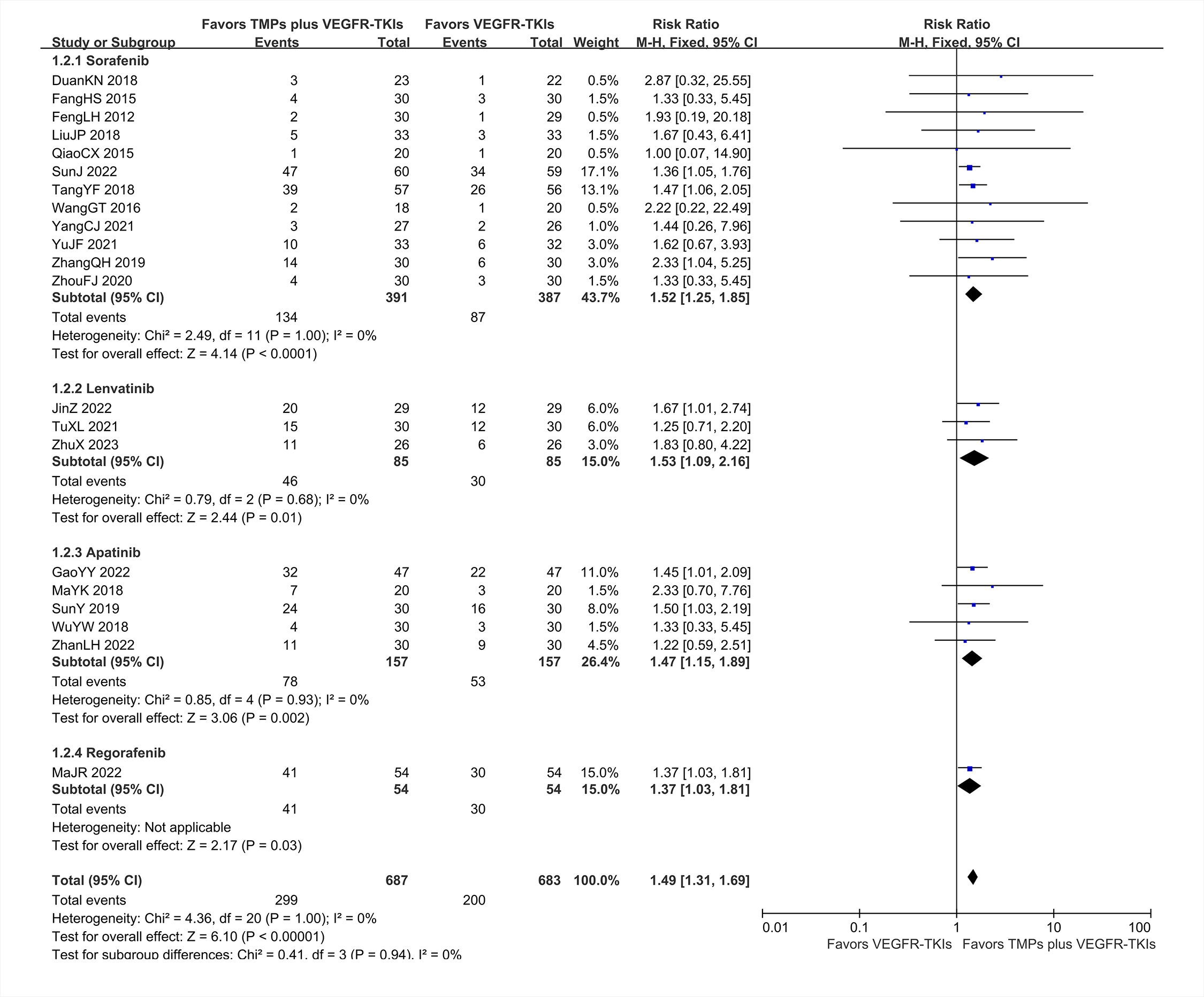


**S1 Fig.** Forest plot and pooled risk ratios for association of objective response rate (ORR) with TMPs and VEGFR-TKIs. Subgroup analysis of different targeted drugs regimen.


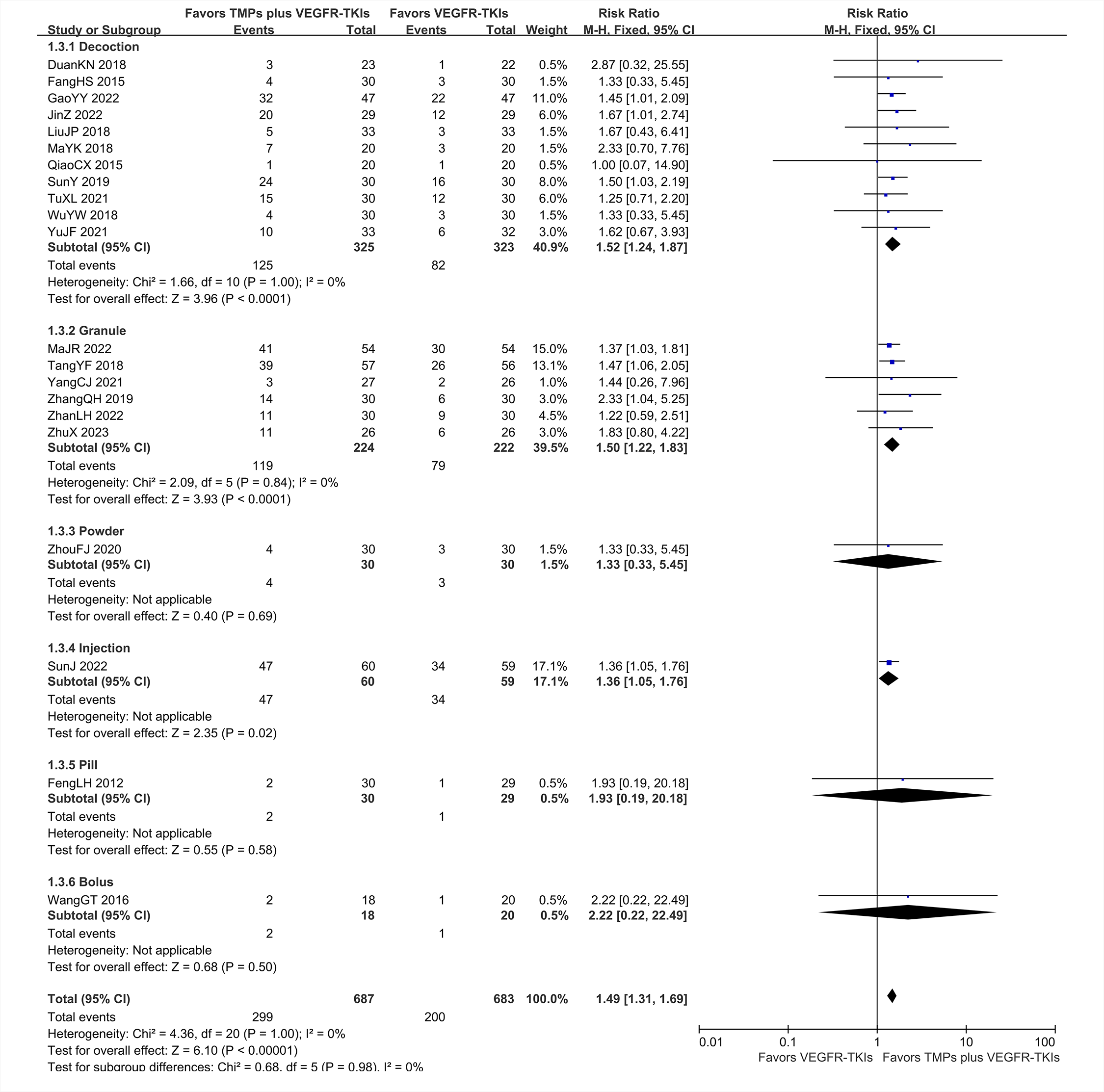


**S2 Fig.** Forest plot and pooled risk ratios for association of objective response rate (ORR) with TMPs and VEGFR-TKIs. Subgroup analysis of different form of TMPs.


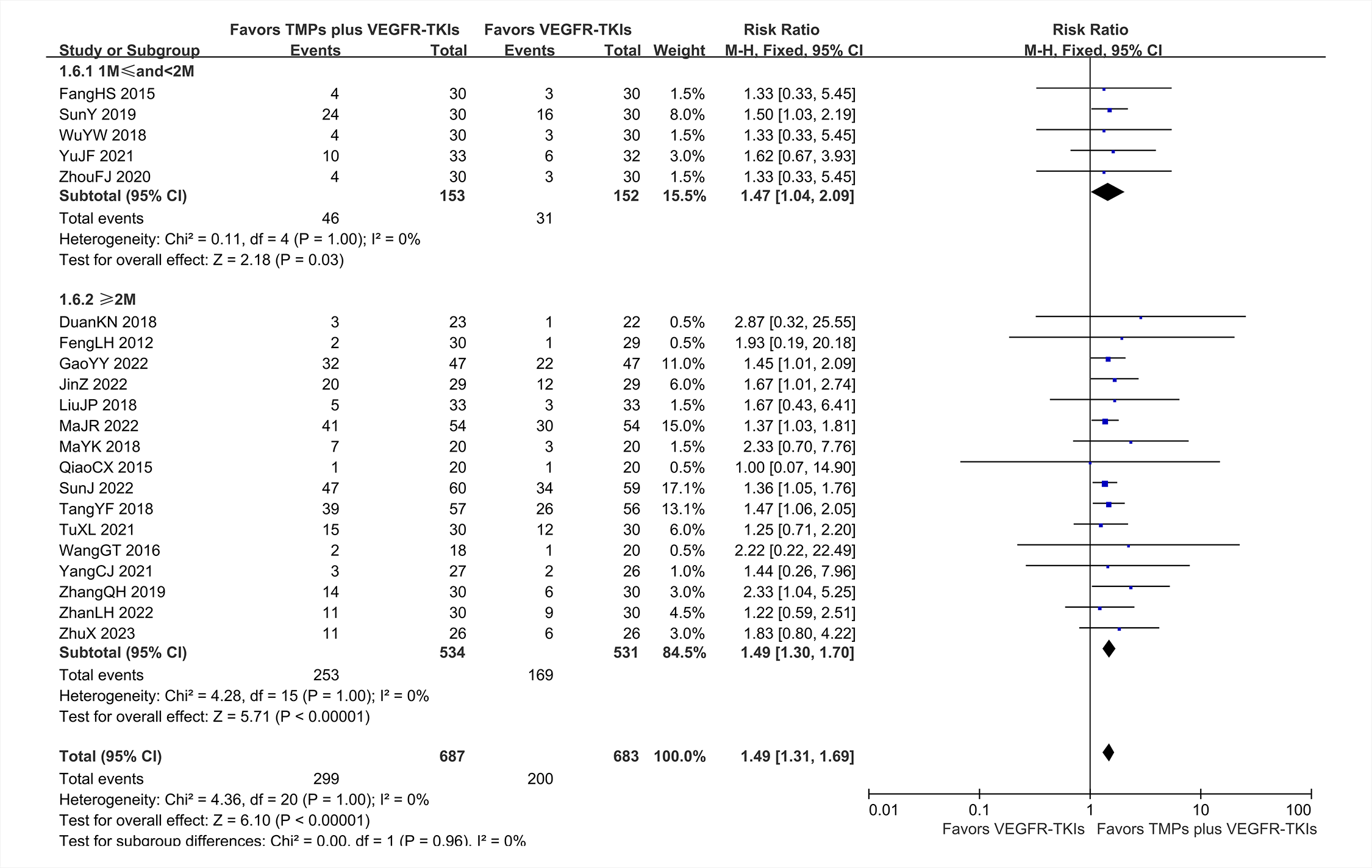


**S3 Fig.** Forest plot and pooled risk ratios for association of objective response rate (ORR) with TMPs and VEGFR-TKIs. Subgroup analysis of the course of treatment.


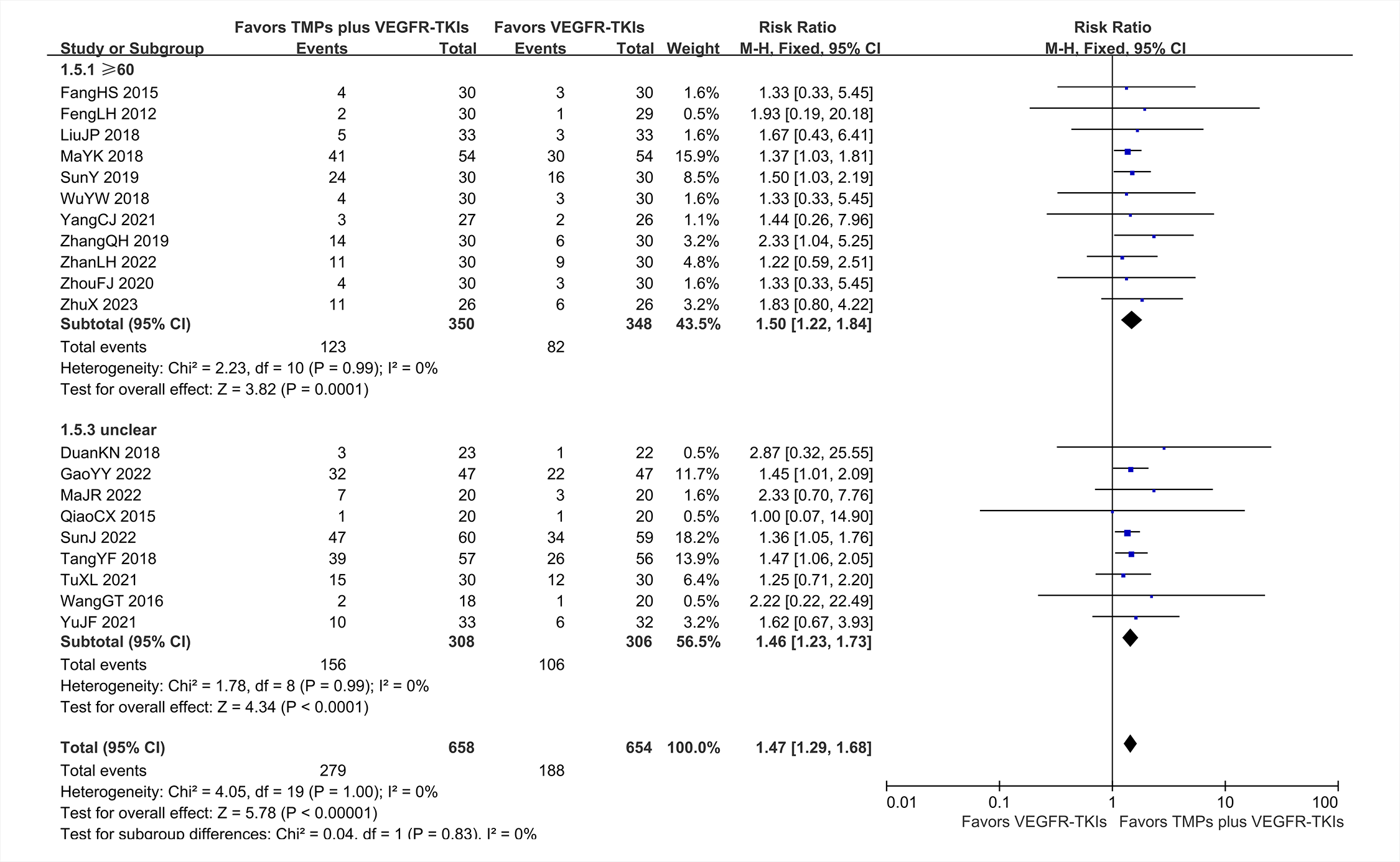


**S4 Fig.** Forest plot and pooled risk ratios for association of objective response rate (ORR) with TMPs and VEGFR-TKIs. Subgroup analysis classification by KPS score.


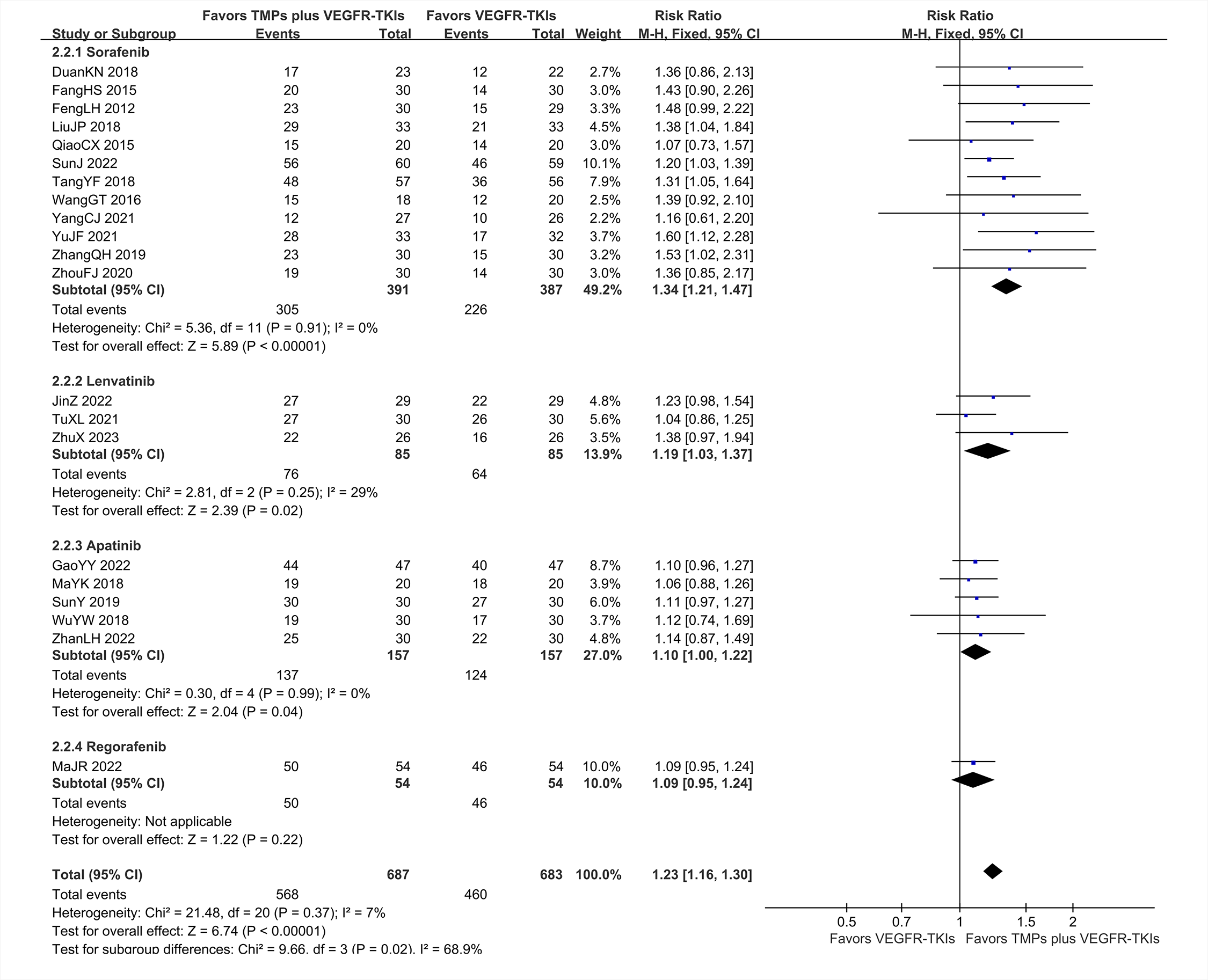


**S5 Fig.** Forest plot and pooled risk ratios for association of objective response rate (DCR) with TMPs and VEGFR-TKIs. Subgroup analysis of different targeted drugs regimen.


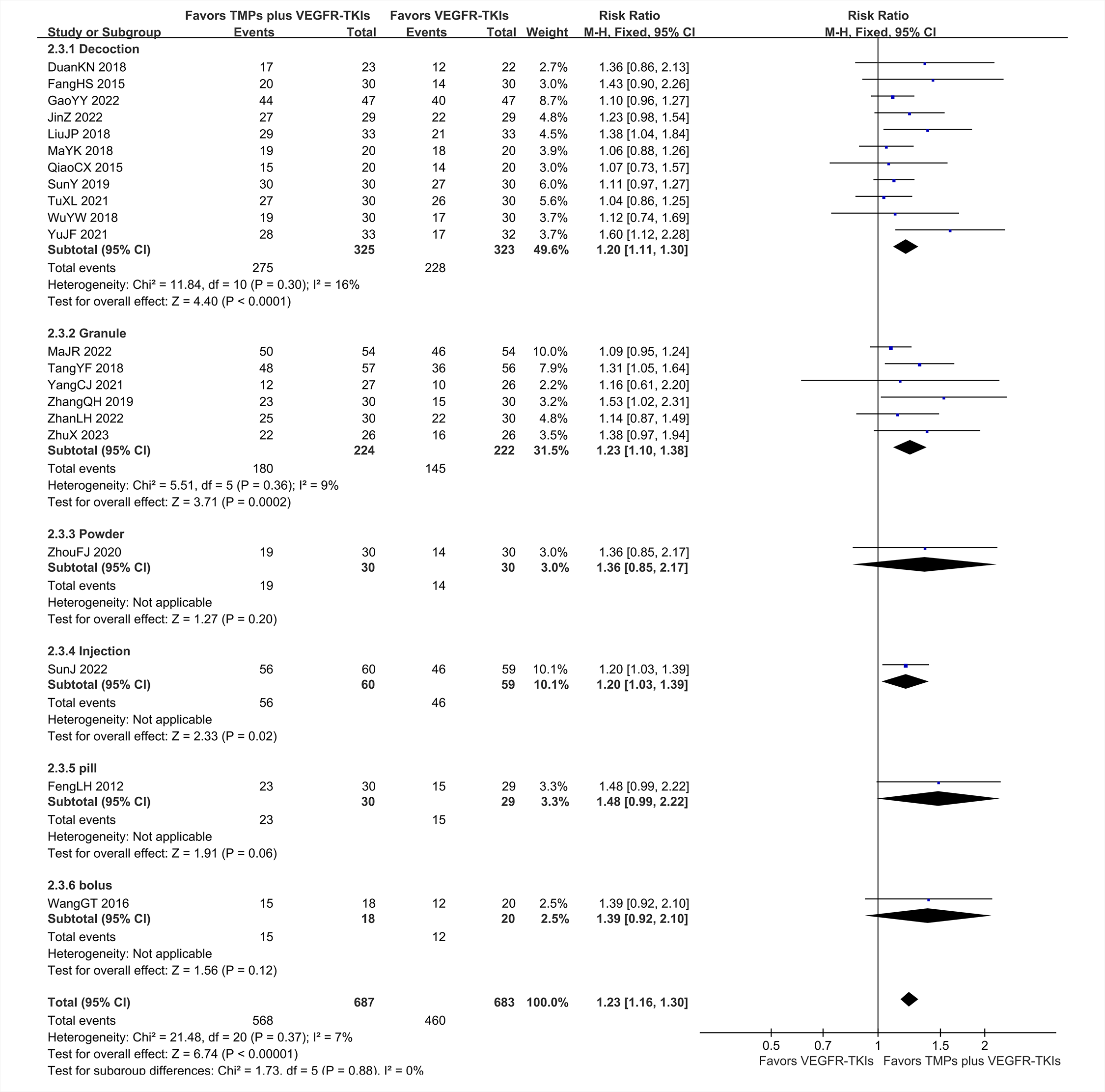


**S6 Fig.** Forest plot and pooled risk ratios for association of objective response rate (DCR) with TMPs and VEGFR-TKIs. Subgroup analysis of different form of TMPs.


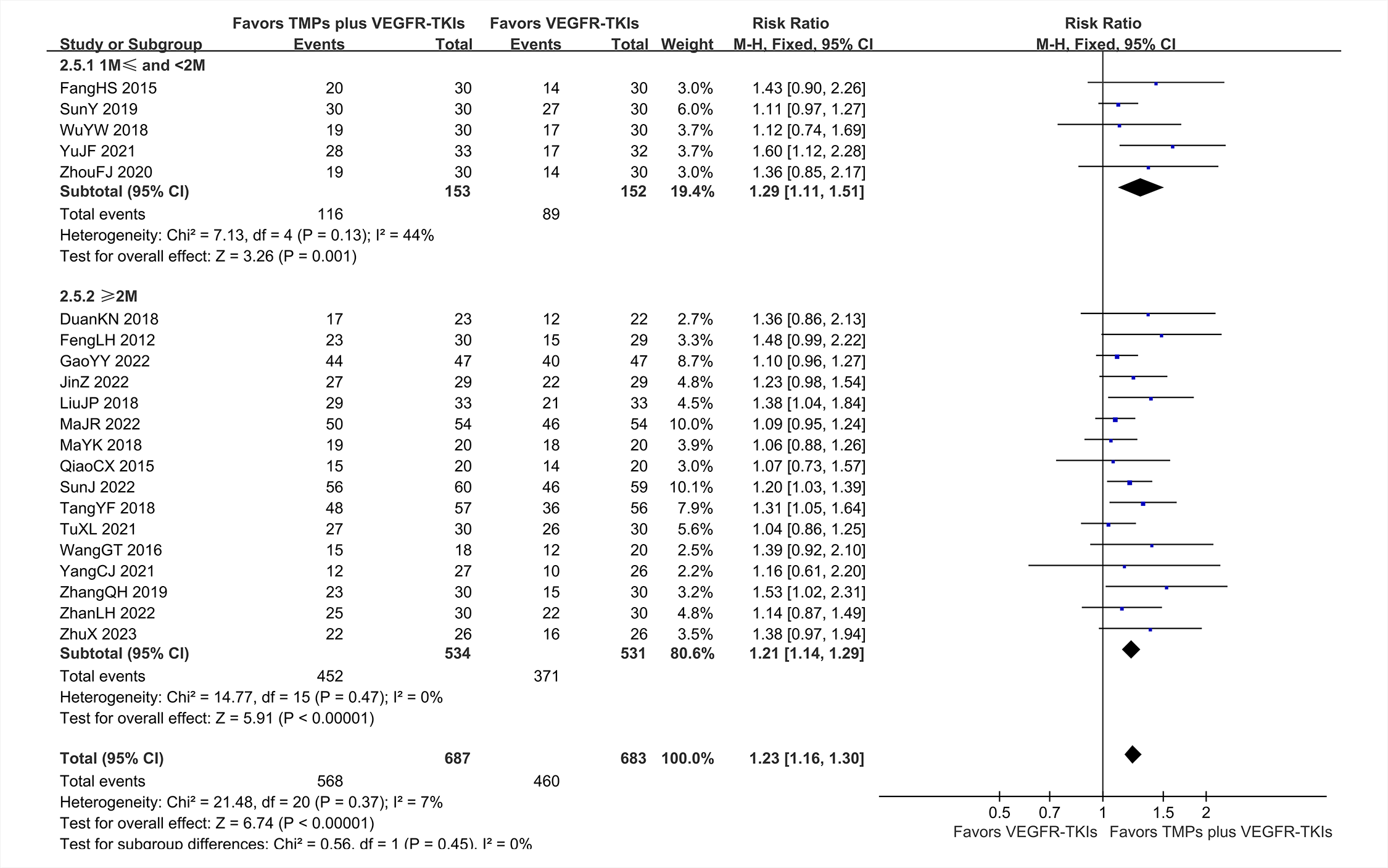


**S7 Fig.** Forest plot and pooled risk ratios for association of objective response rate (DCR) with TMPs and VEGFR-TKIs. Subgroup analysis of the course of treatment.


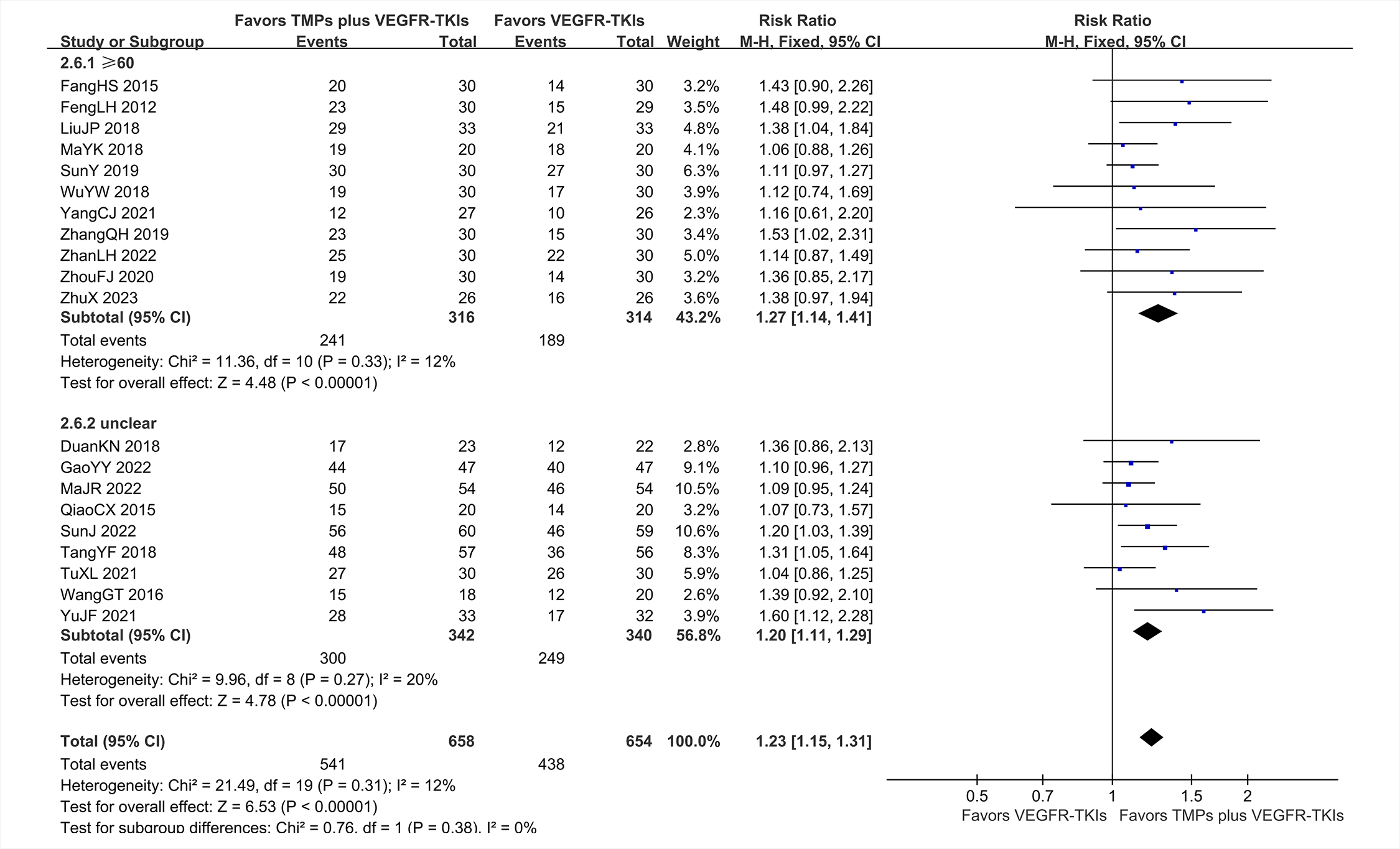


**S8 Fig.** Forest plot and pooled risk ratios for association of objective response rate (DCR) with TMPs and VEGFR-TKIs. Subgroup analysis classification by KPS score.


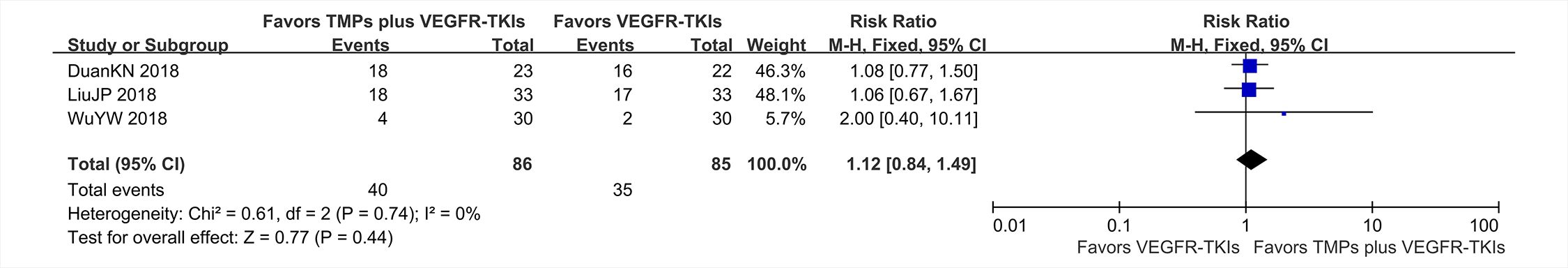


**S9 Fig.** Forest plot and pooled risk ratios for association of QoL (dichotomous data) with TMPs and VEGFR-TKIs.


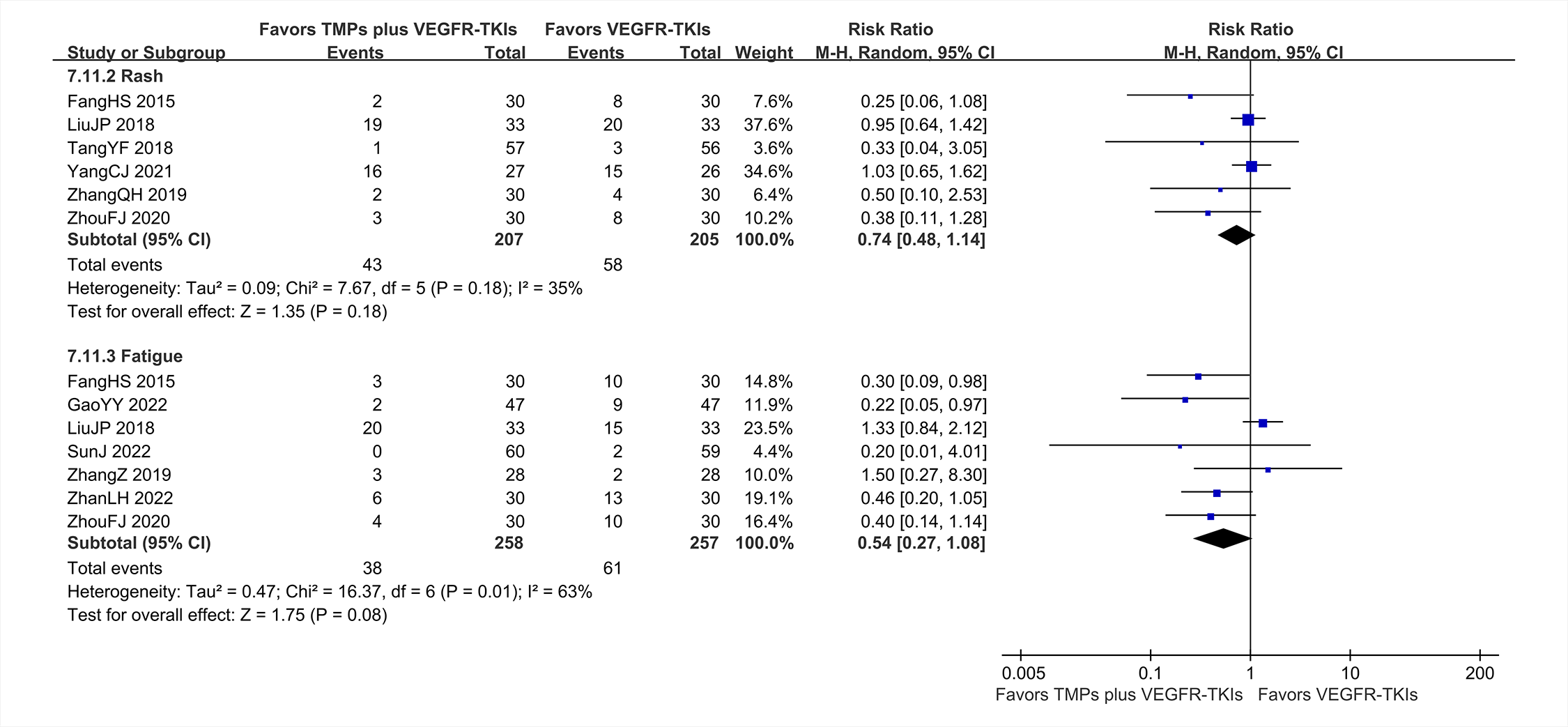


**S10 Fig.** Forest plot and pooled risk ratios for association of rash, and fatigue with TMPs and VEGFR-TKIs.

**S1 Table The results of QoL by continuous data in each included study.**

| Study ID | TMPs plus VEGFR-TKIs | | | | VEGFR-TKIs | | | |  |
| --- | --- | --- | --- | --- | --- | --- | --- | --- | --- |
|  | Mean | | SD | Total | Mean | | SD | Total |  |
| HanGM 2021 | 1.37 | | 7.2 | 29 | -2.31 | | 7.17 | 29 |  |
| JiangXQ 2022 | 25.23 | | 7.21 | 37 | 15 | | 6.47 | 38 |  |
| TangYF 2018 | 24.53 | | 7.79 | 57 | 12.23 | | 6.85 | 56 |  |
| TuXL 2021 | 15.29 | | 5.8 | 30 | 5.62 | | 6.95 | 30 |  |
| WuYW 2018 | 1.34 | | 7.97 | 30 | -7.66 | | 8.59 | 30 |  |
| YangCJ 2021 | 8.52 | | 9.98 | 27 | 0.77 | | 9.11 | 26 |  |
| ZhanLH 2022 | 16.1 | | 4.42 | 30 | -1.16 | | 2.94 | 30 |  |
|  | | M(Q1,Q3) | | | | M(Q1,Q3) | | | |
| ZhuX 2023 | | 80(67.5, 82.5) | | | | 70(60, 80) | | | |

**S2 Table The results of AFP by continuous data in each included study.**

| Study ID | TMPs plus VEGFR-TKIs | | | VEGFR-TKIs | | |
| --- | --- | --- | --- | --- | --- | --- |
|  | Mean | SD | Total | Mean | SD | Total |
| FangHS 2015 | -359.83 | 109.53 | 30 | -266.23 | 165.94 | 30 |
| GaoYY 2022 | -212.38 | 34.83 | 47 | -179.93 | 37.84 | 47 |
| JiangXQ 2022 | -145.06 | 41.49 | 37 | -101.11 | 41.56 | 38 |
| JinZ 2022 | -215.81 | 58.88 | 29 | -151.01 | 59.44 | 29 |
| LiuJP 2018 | -491.4 | 102.51 | 33 | -207.4 | 105.15 | 33 |
| TangYF 2018 | -161.2 | 28.67 | 57 | -90.34 | 29.56 | 56 |
| WangGT 2016 | -491.7 | 108.9 | 18 | -208.7 | 105.93 | 20 |
| ZhanLH 2022 | -326.71 | 41.36 | 30 | -313.96 | 40.06 | 30 |
| ZhouFJ 2020 | -359.83 | 90.26 | 30 | -267.25 | 114.87 | 30 |
|  | M(Q1,Q3) | | | M(Q1,Q3) | | |
| MaJR 2022 | 295.06(176.53, 548.50) | | | 338.42(261.64, 591.14) | | |
| WuYW 2018 | 29.58(13.00, 784.75) | | | 31.42(6.60, 1420) | | |
| YangCJ 2021 | 346(74, 505) | | | 181.5(55.25, 684.75) | | |
| ZhuX 2023 | 151.20(24.60, 531.00) | | | 449.65(56.87, 1331.03) | | |

**S3 Table The incidence of different ADRs.**

| **Outcomes** | **Overall Incidence** | **Experimental group** | | **Control group** | |
| --- | --- | --- | --- | --- | --- |
|  |  | **Events/Total** | **Incidence** | **Events/Total** | **Incidence** |
| Liver dysfunction  (Dichotomous data) | 23.28% | 32/176 | 18.18% | 49/172 | 28.49% |
| Proteinuria | 20.57% | 25/209 | 11.96% | 61/209 | 29.19% |
| Hypertension | 21.78% | 87/503 | 17.30% | 131/498 | 26.31% |
| Hand-foot skin reactions | 19.15% | 70/474 | 14.77% | 111/471 | 23.57% |
| Myelosuppression | 20.53% | 48/301 | 15.95% | 75/298 | 25.17% |
| Gastrointestinal reactions | 20.93% | 85/494 | 17.21% | 121/490 | 24.69% |
| Rash | 24.51% | 43/207 | 20.77% | 58/205 | 28.29% |
| Fatigue | 19.22% | 38/258 | 14.73% | 61/257 | 23.74% |

**S4 Table Meta-analysis results of adverse drug reactions**

| **Outcomes** | **Number of trials** | **Experimental group**  **(Events/Total)** | **Control group**  **(Events/Total)** | **SM** | **RR,95%CI** | **Z** | ***P*** | **Heterogeneity** | |
| --- | --- | --- | --- | --- | --- | --- | --- | --- | --- |
|  |  |  |  |  |  |  |  | ***I^2^*** | ***P_h_*** |
| Liver dysfunction  (Dichotomous data) | 5 | 32/176 | 49/172 | FEM | 0.64 [0.45, 0.91] | 2.49 | 0.01 | 0% | 0.77 |
| Proteinuria | 7 | 25/209 | 61/209 | REM | 0.43 [0.24, 0.75] | 2.97 | 0.003 | 33% | 0.17 |
| Hypertension | 14 | 87/503 | 131/498 | FEM | 0.66 [0.53, 0.83] | 3.63 | 0.0003 | 0% | 0.93 |
| Hand-foot skin reactions | 14 | 70/474 | 111/471 | FEM | 0.63 [0.49, 0.80] | 3.69 | 0.0002 | 0% | 0.70 |
| Myelosuppression | 10 | 48/301 | 75/298 | FEM | 0.63 [0.46, 0.87] | 2.82 | 0.005 | 0% | 0.98 |
| Gastrointestinal reactions | 14 | 85/494 | 121/490 | REM | 0.64 [0.45, 0.92] | 2.39 | 0.02 | 50% | 0.02 |
| Rash | 6 | 43/207 | 58/205 | REM | 0.74 [0.48, 1.14] | 1.35 | 0.18 | 35% | 0.18 |
| Fatigue | 7 | 38/258 | 61/257 | REM | 0.54 [0.27, 1.08] | 1.75 | 0.08 | 63% | 0.01 |

**S5 Table** The Pharmacological action and mechanisms of the 12 traditional medicines with significant effects.

| Traditional medicine name | Components/extractions | Pharmacological action and mechanisms |
| --- | --- | --- |
| *Panax ginseng* C.A.Mey. | Ginsenoside Rg3, ginsenoside Rh2, ginsenoside Rg5, ginsenoside Rk3 | Inhibits the growth of various liver cancer cell lines, including HepG2, MHCC-97L, SMMC-7721, BEL-7404, MHCC-97H, inhibits migration of HCC cells, ARHGAP9 protein↓(Sun et al., 2019); LPS-TLR4↓(Qu et al., 2021); hepatocyte receptor A2 signaling pathway of erythropoietin↓(Kim et al., 2021); induce cell cycle G1 arrest by CDK2↓, cyclin D1↓, PCNA↓, H3K18ac↑, and H4K16ac↑(Li et al., 2018; Shan et al., 2019); inhibit angiogenesis by VEGF↓(Teng et al., 2017), EGF-EGFR-ERK1/2-HIF-1α↓(Li et al., 2018) |
| Huaier granule | Trametes robiniophila Murr | Inhibits proliferation and migration of HCC cells, Yes-associated protein 1(YAP1)↓(Shan et al., 2017); Suppresses the HCC Cell Cycle, minichromosome maintenance(MCM)↓(Niu et al., 2020); cell cycle arrest in S phase↑,caspase 3 ↑,PARP↑, p-ERK↓, p-P38↓, p-JNK↓, β-catenin↓, cyclin D1↓(Zhang et al., 2015); anti-angiogenesis, anti-metastatic, CEACAM1↑, HBx↓(Zhong et al., 2018);HIF-1α/VEGF↓, AUF-1/AEG-1↓, PNCA↓(Li, Wu, et al., 2015) |
| *Citrus × aurantium* L. [Rutaceae] | Nobiletin | suppress the proliferation, induce apoptosis, Bcl-2↓, COX-2↓,Bax ↑, caspase-3↑(Ma, Jin, et al., 2014);attenuates HGF-induced HepG2 cells metastasis, ERK↓, PI3K/Akt↓(Shi et al., 2013);epithelial-to-mesenchymal transition (EMT)↓,STAT3↓, NF-κB↓, AKT↓, PI3K↓, Wnt↓(Ashrafizadeh et al., 2020);multidrug resistance↓(Feng, Zhou, et al., 2020);anti-tumor effect↑(Feng, Tian, et al., 2020; Ma et al., 2015) |
| *Angelica sinensis* (Oliv.) Diels [Apiaceae] | Acetone extract of Angelica sinensis, polysaccharides extract of Angelica sinensis | Inhibits invasion and metastasis of HCC (Zhang et al., 2016); G0/G1 phase↓ (Shang et al., 2003; Zhao, Feng, Jing, Liu, Liu, 2021); induce cell cycle arrest and apoptosis, Bcl-2 oncoprotein↓, cdk4 protein↓(Cheng et al., 2004) |
| *Rehmannia glutinosa* (Gaertn.) DC. [Orobanchaceae] | Catalpol | synergistically potentiate the anti-tumor effects of regorafenib in HCC, VEGF/VEGFR2↓, PI3K/Akt/mTOR↓, NF‑κB↓(El-Hanboshy et al., 2021); miR-22-3p/MTA3 axis ↑(Zhao et al., 2019) |
| *Solanum nigrum* L. [Solanaceae] | solamargine,Polyphenol-rich extracts from Solanum nigrum | reduce tumor growth and angiogenesis, VEGF↓, AKT↓, mTOR↓(Yang et al., 2016); caspase-3↑,cell cycle arrest in G2/M phase↑(Ding, Zhu, Li, Gao, 2012); protein kinase C (PKC)↓(Yang et al., 2010); cellular mitochondria-mediated apoptosis↑,NF-κB↓, iNOS↓(LeeLim, 2008); autophagocytosis↑,Bcl-2↓,Akt↓(Lin et al., 2007) |
| *Prunus persica* (L.) Batsch [Rosaceae] | Amygdalin | Inhibits the growth of HepG2 cells, M/G2 phase↓(Shen et al., 2017); induce apoptosis(Mamdouh et al., 2021) via caspase-3↑, Bcl-2↓(Hosny et al., 2021); promotes the activity of T cells, suppress the progression of HBV-related hepatocellular carcinoma via JAK2/STAT3↓(Wang et al., 2021) ; inhibits HepG2 cell proliferation via Beclin-1↑(Hosny et al., 2021) |

**Table S5** The Pharmacological action and mechanisms of the 11 traditional medicines with significant effects (continued).

| Traditional medicine name | Components/extractions | Pharmacological action and mechanisms |
| --- | --- | --- |
| *Scutellaria barbata* D.Don [Lamiaceae] | total flavonoids of S. barbata (TF-SB), S. barbata polysaccharide (SBP), methanol extracts from Scutellaria barbata, Neo-Clerodane Diterpenoids, Pheophorbide a （Pa） | antitumor and anti-angiogenic activities,Akt/protein kinase B↓(Zhao et al., 2012), MMP↓,TIMP↑(Dai, Wang, et al., 2013); P53↑, Bax/Bcl-2↑(Su et al., 2022); cytotoxic activities(Dai, Tao, Liu, Jiang, Shen, 2006; Wang, Ma, Chen, Li, Chen, 2019; Yang et al., 2018); multidrug resistance↓(Tang et al., 2007); hepatic injury↓(Dai, Wu, et al., 2013) |
| *Curcuma aromatica* Salisb. [Zingiberaceae] | Curumol, β-elemene | inhibit cell proliferation, increase HCC cells apoptosis, DJ-1↓, PTEN↓, PI3K↓, AKT↓(Zhang et al., 2022); zeste homolog 2 (EZH2)↓(Tian, Zheng, Li, Zhang, Zhang, 2021); programmed cell death-ligand 1(PD-L1)↓,crosstalk between hypoxia-inducible factor-1α (HIF-1α)↓, phosphorylated signal transducer and activator of transcription 3 (p-STAT3)↓, S-phase progression↓, tube formation↓, invasion↓, metastasis↓,cytotoxic T-cells↑(Zuo et al., 2020); Fas/FasL↑(Dai, Tang, et al., 2013) |
| *Eupolyphaga sinensis* Walker | polysaccharide from *Eupolyphaga sinensis* walker | lymphocyte proliferation↑(Xie et al., 2020); angiogenesis↓, KDR↓, AKT↓, ERK1/2↓(Dai, Qi, Liu, Zhang, 2014), PKC↓ MAPK↓(Zhang et al., 2014) |
| *Scolopendra subspinipes mutilans* | Extraction | inhibit the proliferation and growth of HCC cells, p-STAT3↓, MAPK↓(Teng et al., 2020); inhibit tumor angiogenesis(Liao et al., 2017); anti-tumor(Ren, Song, et al., 2020; Yan, Lu, Li, Wei, Ren, 2018) |
| *Manis pentadactyla* Linnaeus | Extraction | apoptosis↑, Bcl-2↓, Bak↑, Bax↑, Bad↑(Ma, Liu, Qi, Zhang, 2014) |

# References

Sun, M. Y., Song, Y. N., Zhang, M., Zhang, C. Y., Zhang, L. J., & Zhang, H. (2019). Ginsenoside Rg3 inhibits the migration and invasion of liver cancer cells by increasing the protein expression of ARHGAP9. *Oncol Lett*, 17(1), 965-973. https://doi.org/10.3892/ol.2018.9701.

Kim, H., Choi, P., Kim, T., Kim, Y., Song, B. G., Park, Y. T., et al. (2021). Ginsenosides Rk1 and Rg5 inhibit transforming growth factor-beta1-induced epithelial-mesenchymal transition and suppress migration, invasion, anoikis resistance, and development of stem-like features in lung cancer. *J Ginseng Res*, 45(1), 134-148. https://doi.org/10.1016/j.jgr.2020.02.005.

Qu, L., Ma, X., & Fan, D. (2021). Ginsenoside Rk3 Suppresses Hepatocellular Carcinoma Development through Targeting the Gut-Liver Axis. *J Agric Food Chem*, 69(35), 10121-10137. https://doi.org/10.1021/acs.jafc.1c03279.

Li, X., Tsauo, J., Geng, C., Zhao, H., Lei, X., & Li, X. (2018). Ginsenoside Rg3 Decreases NHE1 Expression via Inhibiting EGF-EGFR-ERK1/2-HIF-1 alpha Pathway in Hepatocellular Carcinoma: A Novel Antitumor Mechanism. *Am J Chin Med*, 46(8), 1915-1931. https://doi.org/10.1142/S0192415X18500969.

Teng, S., Wang, Y., Li, P., Liu, J., Wei, A., Wang, H., et al. (2017). Effects of R type and S type ginsenoside Rg3 on DNA methylation in human hepatocarcinoma cells. *Mol Med Rep*, 15(4), 2029-2038. https://doi.org/10.3892/mmr.2017.6255.

Zhang, C., Zhang, J., Li, X., Sun, N., Yu, R., Zhao, B., et al. 2015. Huaier Aqueous Extract Induces Hepatocellular Carcinoma Cells Arrest in S Phase via JNK Signaling Pathway. *Evid Based Complement Alternat Med, 2015*, 171356. https://doi.org/10.1155/2015/171356.

Zhong, L. H., Zhu, L. Y., Zhao, Y. Y., Wang, W., Lu, B. L., Wang, Y., et al. 2018. Apoptosis of hepatocarcinoma cells Hepg2 induced by Huaier extract through regulation of HBx and CEACAM1 gene expression. *J Biol Regul Homeost Agents, 32*(6), 1389-1398.

Li, C., Wu, X., Zhang, H., Yang, G., Hao, M., Sheng, S., et al. 2015. A Huaier polysaccharide restrains hepatocellular carcinoma growth and metastasis by suppression angiogenesis. *Int J Biol Macromol, 75*, 115-120. https://doi.org/10.1016/j.ijbiomac.2015.01.016.

Shi, M. D., Liao, Y. C., Shih, Y. W., & Tsai, L. Y. 2013. Nobiletin attenuates metastasis via both ERK and PI3K/Akt pathways in HGF-treated liver cancer HepG2 cells. *Phytomedicine, 20*(8-9), 743-752. https://doi.org/10.1016/j.phymed.2013.02.004.

Ashrafizadeh, M., Zarrabi, A., Saberifar, S., Hashemi, F., Hushmandi, K., Hashemi, F., et al. 2020. Nobiletin in Cancer Therapy: How This Plant Derived-Natural Compound Targets Various Oncogene and Onco-Suppressor Pathways. *Biomedicines, 8*(5). https://doi.org/10.3390/biomedicines8050110.

Feng, S. L., Tian, Y., Huo, S., Qu, B., Liu, R. M., Xu, P., et al. 2020. Nobiletin potentiates paclitaxel anticancer efficacy in A549/T xenograft model: Pharmacokinetic and pharmacological study. *Phytomedicine, 67*, 153141. https://doi.org/10.1016/j.phymed.2019.153141.

Feng, S., Zhou, H., Wu, D., Zheng, D., Qu, B., Liu, R., et al. 2020. Nobiletin and its derivatives overcome multidrug resistance (MDR) in cancer: total synthesis and discovery of potent MDR reversal agents. *Acta Pharm Sin B, 10*(2), 327-343. https://doi.org/10.1016/j.apsb.2019.07.007.

Ma, W., Feng, S., Yao, X., Yuan, Z., Liu, L., & Xie, Y. 2015. Nobiletin enhances the efficacy of chemotherapeutic agents in ABCB1 overexpression cancer cells. *Sci Rep, 5*, 18789. https://doi.org/10.1038/srep18789.

Zhang, Y., Zhou, T., Wang, H., Cui, Z., Cheng, F., & Wang, K. P. 2016. Structural characterization and in vitro antitumor activity of an acidic polysaccharide from Angelica sinensis (Oliv.) Diels. *Carbohydr Polym, 147*, 401-408. https://doi.org/10.1016/j.carbpol.2016.04.002.

Shang, P., Qian, A. R., Yang, T. H., Jia, M., Mei, Q. B., Cho, C. H., et al. 2003. Experimental study of anti-tumor effects of polysaccharides from Angelica sinensis. *World J Gastroenterol, 9*(9), 1963-1967. <https://doi.org/10.3748/wjg.v9.i9.1963>.

Zhao, Y., Feng, Y., Jing, X., Liu, Y., & Liu, A. 2021. Structural Characterization of an Alkali-Soluble Polysaccharide from Angelica sinensis and Its Antitumor Activity in Vivo. *Chem Biodivers, 18*(6), e2100089. https://doi.org/10.1002/cbdv.202100089.

Cheng, Y. L., Chang, W. L., Lee, S. C., Liu, Y. G., Chen, C. J., Lin, S. Z., et al. 2004. Acetone extract of Angelica sinensis inhibits proliferation of human cancer cells via inducing cell cycle arrest and apoptosis. *Life Sci, 75*(13), 1579-1594. https://doi.org/10.1016/j.lfs.2004.03.009.

El-Hanboshy, S. M., Helmy, M. W., & Abd-Alhaseeb, M. M. 2021. Catalpol synergistically potentiates the anti-tumour effects of regorafenib against hepatocellular carcinoma via dual inhibition of PI3K/Akt/mTOR/NF-kappaB and VEGF/VEGFR2 signaling pathways. *Mol Biol Rep, 48*(11), 7233-7242. https://doi.org/10.1007/s11033-021-06715-0.

Zhao, L., Wang, Y., & Liu, Q. 2019. Catalpol inhibits cell proliferation, invasion and migration through regulating miR-22-3p/MTA3 signalling in hepatocellular carcinoma. *Exp Mol Pathol, 109*, 51-60. https://doi.org/10.1016/j.yexmp.2019.104265.

Yang, M. Y., Hung, C. H., Chang, C. H., Tseng, T. H., & Wang, C. J. 2016. Solanum nigrum Suppress Angiogenesis-Mediated Tumor Growth Through Inhibition of the AKT/mTOR Pathway. *Am J Chin Med, 44*(6), 1273-1288. https://doi.org/10.1142/S0192415X16500713.

Ding, X., Zhu, F. S., Li, M., & Gao, S. G. 2012. Induction of apoptosis in human hepatoma SMMC-7721 cells by solamargine from Solanum nigrum L. *J Ethnopharmacol, 139*(2), 599-604. https://doi.org/10.1016/j.jep.2011.11.058.

Yang, M. Y., Hsu, L. S., Peng, C. H., Shi, Y. S., Wu, C. H., & Wang, C. J. 2010. Polyphenol-rich extracts from Solanum nigrum attenuated PKC alpha-mediated migration and invasion of hepatocellular carcinoma cells. *J Agric Food Chem, 58*(9), 5806-5814. https://doi.org/10.1021/jf100718b.

Lee, S. J., & Lim, K. T. 2008. Cell death signal by glycine- and proline-rich plant glycoprotein is transferred from cytochrome c and nuclear factor kappa B to caspase 3 in Hep3B cells. *J Nutr Biochem, 19*(3), 166-174. https://doi.org/10.1016/j.jnutbio.2007.02.006.

Lin, H. M., Tseng, H. C., Wang, C. J., Chyau, C. C., Liao, K. K., Peng, P. L., et al. 2007. Induction of autophagy and apoptosis by the extract of Solanum nigrum Linn in HepG2 cells. *J Agric Food Chem, 55*(9), 3620-3628. https://doi.org/10.1021/jf062406m.

Shen, H., Wang, H., Wang, L., Wang, L., Zhu, M., Ming, Y., et al. (2017). Ethanol Extract of Root of Prunus persica Inhibited the Growth of Liver Cancer Cell HepG2 by Inducing Cell Cycle Arrest and Migration Suppression. *Evid Based Complement Alternat Med*, 2017, 8231936. <https://doi.org/10.1155/2017/8231936>.

Mamdouh, A. M., Khodeer, D. M., Tantawy, M. A., & Moustafa, Y. M. (2021). In-vitro and in-vivo investigation of amygdalin, metformin, and combination of both against doxorubicin on hepatocellular carcinoma. *Life Sci*, 285, 119961. https://doi.org/10.1016/j.lfs.2021.119961.

Hosny, S., Sahyon, H., Youssef, M., & Negm, A. (2021). Prunus Armeniaca L. Seed Extract and Its Amygdalin Containing Fraction Induced Mitochondrial-Mediated Apoptosis and Autophagy in Liver Carcinogenesis. *Anticancer Agents Med Chem*, 21(5), 621-629. https://doi.org/10.2174/1871520620666200608124003.

Wang, R., Zhang, D., Sun, K., Peng, J., Zhu, W., Yin, S., et al. (2021). Amygdalin promotes the activity of T cells to suppress the progression of HBV-related hepatocellular carcinoma via the JAK2/STAT3 signaling pathway. *BMC Infect Dis*, 21(1), 56. https://doi.org/10.1186/s12879-020-05713-0.

Zhao, Z., Holle, L., Song, W., Wei, Y., Wagner, T. E., & Yu, X. 2012. Antitumor and anti-angiogenic activities of Scutellaria barbata extracts in vitro are partially mediated by inhibition of Akt/protein kinase B. *Mol Med Rep, 5*(3), 788-792. https://doi.org/10.3892/mmr.2011.694.

Dai, Z. J., Wang, B. F., Lu, W. F., Wang, Z. D., Ma, X. B., Min, W. L., et al. 2013. Total flavonoids of Scutellaria barbata inhibit invasion of hepatocarcinoma via MMP/TIMP in vitro. *Molecules, 18*(1), 934-950. https://doi.org/10.3390/molecules18010934.

Su, W., Wu, L., Liang, Q., Lin, X., Xu, X., Yu, S., et al. 2022. Extraction Optimization, Structural Characterization, and Anti-Hepatoma Activity of Acidic Polysaccharides From Scutellaria barbata D. Don. *Front Pharmacol, 13*, 827782. https://doi.org/10.3389/fphar.2022.827782.

Dai, S. J., Tao, J. Y., Liu, K., Jiang, Y. T., & Shen, L. 2006. neo-Clerodane diterpenoids from Scutellaria barbata with cytotoxic activities. *Phytochemistry, 67*(13), 1326-1330. https://doi.org/10.1016/j.phytochem.2006.04.024.

Wang, M., Ma, C., Chen, Y., Li, X., & Chen, J. 2019. Cytotoxic Neo-Clerodane Diterpenoids from Scutellaria barbata D.Don. *Chem Biodivers, 16*(2), e1800499. <https://doi.org/10.1002/cbdv.201800499>.

Yang, G. C., Hu, J. H., Li, B. L., Liu, H., Wang, J. Y., & Sun, L. X. 2018. Six New neo-Clerodane Diterpenoids from Aerial Parts of Scutellaria barbata and Their Cytotoxic Activities. *Planta Med, 84*(17), 1292-1299. https://doi.org/10.1055/a-0638-8255.

Tang, P. M., Chan, J. Y., Zhang, D. M., Au, S. W., Fong, W. P., Kong, S. K., et al. 2007. Pheophorbide a, an active component in Scutellaria barbata, reverses P-glycoprotein-mediated multidrug resistance on a human hepatoma cell line R-HepG2. *Cancer Biol Ther, 6*(4), 504-509. https://doi.org/10.4161/cbt.6.4.3814.

Dai, Z. J., Wu, W. Y., Kang, H. F., Ma, X. B., Zhang, S. Q., Min, W. L., et al. 2013. Protective effects of Scutellaria barbata against rat liver tumorigenesis. *Asian Pac J Cancer Prev, 14*(1), 261-265. https://doi.org/10.7314/apjcp.2013.14.1.261.

Zhang, R., Zhong, L., Sun, K., Liu, J., Wang, Q., Mao, D., et al. 2022. A Study on Curcumol Influencing Proliferation and Apoptosis of Hepatocellular Carcinoma Cells through DJ-1/PTEN/PI3K/AKT Pathway. *Biomed Res Int, 2022*, 9912776. https://doi.org/10.1155/2022/9912776.

Tian, N. N., Zheng, Y. B., Li, Z. P., Zhang, F. W., & Zhang, J. F. 2021. Histone methylatic modification mediates the tumor-suppressive activity of curcumol in hepatocellular carcinoma via an Hotair/EZH2 regulatory axis. *J Ethnopharmacol, 280*, 114413. https://doi.org/10.1016/j.jep.2021.114413.

Zuo, H. X., Jin, Y., Wang, Z., Li, M. Y., Zhang, Z. H., Wang, J. Y., et al. 2020. Curcumol inhibits the expression of programmed cell death-ligand 1 through crosstalk between hypoxia-inducible factor-1alpha and STAT3 (T705) signaling pathways in hepatic cancer. *J Ethnopharmacol, 257*, 112835. https://doi.org/10.1016/j.jep.2020.112835.

Dai, Z. J., Tang, W., Lu, W. F., Gao, J., Kang, H. F., Ma, X. B., et al. 2013. Antiproliferative and apoptotic effects of beta-elemene on human hepatoma HepG2 cells. *Cancer Cell Int, 13*(1), 27. https://doi.org/10.1186/1475-2867-13-27.

Xie, X., Shen, W., Zhou, Y., Ma, L., Xu, D., Ding, J., et al. 2020. Characterization of a polysaccharide from Eupolyphaga sinensis walker and its effective antitumor activity via lymphocyte activation. *Int J Biol Macromol, 162*, 31-42. https://doi.org/10.1016/j.ijbiomac.2020.06.120.

Dai, B., Qi, J., Liu, R., & Zhang, Y. 2014. Eupolyphaga sinensis Walker demonstrates angiogenic activity and inhibits A549 cell growth by targeting the KDR signaling pathway. *Mol Med Rep, 10*(3), 1590-1596. https://doi.org/10.3892/mmr.2014.2387.

Zhang, Y., Zhan, Y., Zhang, D., Dai, B., Ma, W., Qi, J., et al. 2014. Eupolyphaga sinensis walker displays inhibition on hepatocellular carcinoma through regulating cell growth and metastasis signaling. *Sci Rep, 4*, 5518. https://doi.org/10.1038/srep05518.

Teng, Y. J., Liu, Z., Liao, L., Chen, Y., Huang, X. D., & Tian, X. F. 2020. STAT3 Inhibition by Centipede Scolopendra Extract in Liver Cancer HepG2 Cells and Orthotopic Mouse Models of Hepatocellular Carcinoma. *Digital Chinese Medicine, 3*(02), 67-79.

Liao, L., Liu, X. B., Zhou, Q., Wang, Z. Q., Tian, S., Chen, Y., et al. 2017. Effect of Scolopendra subspinipes extracts on STAT3 signaling pathway of human hepatocellular carcinoma HepG2 cells. *Chin Tradit Herb Drugs, 48*(05), 930-934. https://doi.org/10.7501/j.issn.0253-2670.2017.05.015.

Ren, Y., Song, H., Wu, Y., Ma, X., Yu, X., Liu, J., et al. 2020. Structural characterization and anticancer potency of centipede oligopeptides in human chondrosarcoma cancer: inducing apoptosis. *RSC Adv, 10*(50), 29780-29788. https://doi.org/10.1039/d0ra04811a.

Yan, W., Lu, J., Li, G., Wei, H., & Ren, W. H. 2018. Amidated Scolopin-2 inhibits proliferation and induces apoptosis of Hela cells in vitro and in vivo. *Biotechnol Appl Biochem, 65*(5), 672-679. https://doi.org/10.1002/bab.1661.

Ma, W., Liu, R., Qi, J., & Zhang, Y. 2014. Extracts of centipede Scolopendra subspinipes mutilans induce cell cycle arrest and apoptosis in A375 human melanoma cells. *Oncol Lett, 8*(1), 414-420. https://doi.org/10.3892/ol.2014.2139.

Shan, L., Li, Y., Jiang, H., Tao, Y., Qian, Z., Li, L., et al. 2017. Huaier Restrains Proliferative and Migratory Potential of Hepatocellular Carcinoma Cells Partially Through Decreased Yes-Associated Protein 1. *J Cancer, 8*(19), 4087-4097. https://doi.org/10.7150/jca.21018.

Niu, Y., Shan, L., Gao, H., Zhang, C., Qian, Z., Wang, Z., et al. 2020. Huaier Suppresses the Hepatocellular Carcinoma Cell Cycle by Regulating Minichromosome Maintenance Proteins. *Onco Targets Ther, 13*, 12015-12025. https://doi.org/10.2147/OTT.S279723.

Ma, X., Jin, S., Zhang, Y., Wan, L., Zhao, Y., & Zhou, L. 2014. Inhibitory effects of nobiletin on hepatocellular carcinoma in vitro and in vivo. *Phytother Res, 28*(4), 560-567. https://doi.org/10.1002/ptr.5024.
