# Supplementary material for: Combinatorial treatment with traditional medicinal preparations and VEGFR-tyrosine kinase inhibitors for middle-advanced primary liver cancer: A systematic review and meta-analysis
Source: PLoS One. 2024 Nov 22;19(11):e0313443. doi: 10.1371/journal.pone.0313443 (PMC11584121; doi:10.1371/journal.pone.0313443)
Supplement: Attachment — Submitted filename: Response to Reviewers.docx [file pone.0313443.s006.docx]

Dear Editors and Reviewers:

Thank you for your letter and for the reviewers’ comments concerning our manuscript entitled “Combinatorial treatment with traditional medicinal preparations and VEGFR-Tyrosine Kinase Inhibitors for middle-advanced primary liver cancer: A systematic review and meta-analysis” [Manuscript ID: (PONE-D-23-16652)]. Those comments are all valuable and very helpful for revising and improving our paper, as well as the important guiding significance to our researches. We have studied comments carefully and have made correction. The main corrections in the manuscript and the responds to the reviewer’s comments are as below.

Please let us know if there is further issue need to be revised.

Kind regards,

Hong-Gang Zheng on behalf of the author team

**Response to Editors：**

**1. Please ensure that your manuscript meets PLOS ONE's style requirements, including those for file naming. The PLOS ONE style templates can be found at**

https://journals.plos.org/plosone/s/file?id=wjVg/PLOSOne_formatting_sample_main_body.pdf and https://journals.plos.org/plosone/s/file?id=ba62/PLOSOne_formatting_sample_title_authors_affiliations.pdf.

**Response:** We appreciate your pointing out our shortcomings. Our manuscript has been revised in PLOS ONE's style.

**2. Please remove any discussion of unvalidated diagnoses or methods, specifically in the penultimate paragraph of the introduction section.**

**Response:** Thank you for pointing out this mistake. We have been removed discussion of unvalidated diagnoses or methods, specifically in the penultimate paragraph of the introduction section.

**3. Thank you for stating in your Funding Statement:“This study was supported by the National Natural Science Foundation of China (Grant No. 82174463), Scientific and Technological Innovation Project of China Academy of Chinese Medical Sciences (No. CI2021A01804), Innovation Team and Talents Cultivation Program of National Administration of Traditional Chinese Medicine (No. ZYYCXTD-C-202205).” Please provide an amended statement that declares *all* the funding or sources of support (whether external or internal to your organization) received during this study, as detailed online in our guide for authors at http://journals.plos.org/plosone/s/submit-now. Please also include the statement “There was no additional external funding received for this study.” in your updated Funding Statement. Please include your amended Funding Statement within your cover letter. We will change the online submission form on your behalf.**

**Response:** Thank you for your comments. We have stated the Funding Statement in cover letter. Please see the below revisions.

*“Funding Statement：*

*This study was supported by the National Natural Science Foundation of China (Grant No. 82174463), the Scientific and Technological Innovation Project of China Academy of Chinese Medical Sciences (No. CI2021A01804), Innovation Team and Talents Cultivation Program of the National Administration of Traditional Chinese Medicine (No. ZYYCXTD-C-202205).*

*There was no additional external funding received for this study.”*

**Response to Reviewers：**

**Reviewer #1:**

**1-We believe this article is suitable for publication in the journal although major revisions are needed. The main strengths of this paper are that it addresses an interesting and very timely question and provides a clear answer, with some limitations. Certainly, the authors should better highlight the limitations of the current paper.**

Response: We appreciate your pointing out our shortcomings. We have further refined and emphasized the limitations of the current paper, specifically outlined in lines 483-501. Please see the below revisions.

“*However, there are several limitations in this study. 1) Only English and Chinese language databases were surveyed, therefore, omissions may exist. All the included studies were sourced from China because of the main location for Chinese medicine research, which may have regional and ethnic influences. Further research is thus required to extend the current findings to other populations and regions. 2) Only 13 studies reported random sequence generation, and none followed the blinding method, which may have introduced potential selection and detection biases. Therefore, the GRADE rating for the evidence quality of these datasets might be low or very low. However, the sensitivity analysis showed that the outcomes were reliable. The data will be constantly updated with the publication of new high-quality studies. 3) Only 8 studies reported the long-term survival in PLC patients, thus, warrants further research. 4) Although all studies reported quality control, only 8 studies included chemical analysis; the active ingredients of TMPs in other studies were unclear and required clarification from the drug manufacturers or research studies, which may cause bias. In addition, sensitivity and sub-cohort analyses were carried out to mitigate the effect of heterogeneity and the resulting bias for specific traditional medicine within TMPs. 5) The majority of the studies reviewed within this investigation did not adhere stringently to reporting standards for CONSORT Extension for Chinese Herbal Medicine Formulas.*”

**2- A linguistic revision is needed.**

Response: Thank you for pointing out this mistake. We have checked and revised the manuscript carefully with the help of experts.

**3- The background of the changing scenario of medical treatment in HCC should be better discussed, and some recent papers regarding this topic should be included (PMID: 32772560; PMID: 36633661; PMID: 34798793; PMID: 35031442).**

**Response:** Thank you for your comments. This point you mentioned is very important. We have incorporated recent advancements in the treatment of HCC, particularly focusing on immunotherapy and combination immunotherapy with targeted therapies, in the “Introduction” section. In addition to citing the four articles suggested by you, we have also included recent relevant literature. Please refer to lines 64-77 for specifics. Please see the below revisions.

*“In recent years, in addition to targeted therapies, significant breakthroughs in immunotherapy have also been made. A meta-analysis revealed that in patients undergoing immunotherapy (either monotherapy or in combination with other anticancer agents), the pooled odds ratio was 1.67 [95% confidence interval (CI): 1.52 - 1.84]. Compared to control treatments, immune checkpoint inhibitors have indicated substantially increased rates of achieving complete response (CR) (15). Moreover, gender was observed to influence the efficacy of immune checkpoint inhibitors in cancer patients, with males generally experiencing greater overall benefit after this therapy (16, 17). It has been indicated that patients with advanced hepatocellular carcinoma (HCC) can benefit from immunotherapy (18). Furthermore, for treating HCC, many studies have revealed the efficacy of immunotherapy, such as Tremelimumab + Durvalumab and Durvalumab alone (19), as well as the combination of immunotherapy with targeted therapy, such as Atezolizumab + Bevacizumab (20). The National Comprehensive Cancer Network (2024) guidelines have also approved these treatment strategies.”*

**References**

15.	Santoni M, Rizzo A, Kucharz J, Mollica V, Rosellini M, Marchetti A, et al. Complete remissions following immunotherapy or immuno-oncology combinations in cancer patients: the MOUSEION-03 meta-analysis. Cancer Immunol Immunother. 2023;72(6):1365-79.

16.	Santoni M, Rizzo A, Mollica V, Matrana MR, Rosellini M, Faloppi L, et al. The impact of gender on The efficacy of immune checkpoint inhibitors in cancer patients: The MOUSEION-01 study. Crit Rev Oncol Hematol. 2022;170:103596.

17.	Conforti F, Pala L, Bagnardi V, De Pas T, Martinetti M, Viale G, et al. Cancer immunotherapy efficacy and patients' sex: a systematic review and meta-analysis. Lancet Oncol. 2018;19(6):737-46.

18.	Rizzo A, Ricci AD, Brandi G. Systemic adjuvant treatment in hepatocellular carcinoma: tempted to do something rather than nothing. Future Oncol. 2020;16(32):2587-9.

19.	Abou-Alfa GK, Lau G, Kudo M, Chan SL, Kelley RK, Furuse J, et al. Tremelimumab plus Durvalumab in Unresectable Hepatocellular Carcinoma. NEJM Evid. 2022;1(8):EVIDoa2100070.

20.	Cheng AL, Qin S, Ikeda M, Galle PR, Ducreux M, Kim TY, et al. Updated efficacy and safety data from IMbrave150: Atezolizumab plus bevacizumab vs. sorafenib for unresectable hepatocellular carcinoma. J Hepatol. 2022;76(4):862-73.

**Reviewer #2:**

**1- Some typos and grammatical errors ought to be corrected through the whole manuscript.**

Response: Thank you for pointing out this mistake. We have checked and revised the manuscript carefully with the help of experts.

**2- The article has too many abbreviations, which might cause confusion to readers. I recommend to reduce the number of abbreviated terms within the manuscript.**

Response: Thank you for the comments. Your comments make our paper more intuitive. We have reduced the use of abbreviated terms in the re-submitted manuscript. All abbreviated terms are now included in the "Abbreviations" section to ensure clarity for the readers.

**3- The introduction suffers from poor discussion about Cancer/VEGFR relationship and VEGFR-TK inhibitors. Please improve.**

**Response:** Thank you for your comments. This point you mentioned is very important. We have incorporated information regarding the Cancer/VEGFR relationship and VEGFR-TK inhibitors into the “Introduction” section in lines 38-52. Please see the below revisions.

*“The Vascular Endothelial Growth Factor (VEGF) family comprises VEGF-A, VEGF-B, VEGF-C, VEGF-D, and placental growth factor (2). These proteins predominantly bind VEGF receptor-1 (VEGFR-1) or Fms-like Tyrosine Kinase-1, and VEGFR-2 (also called kinase insert domain-containing receptor) (2, 3). This interaction activates downstream signaling pathways crucial for endothelial cell proliferation, differentiation, and migration, as well as the regulation of vascular permeability, which are essential for angiogenesis. Angiogenesis ensures a steady supply of oxygen and nutrients during tumor development and progression. VEGF, secreted by tumor cells and their microenvironment, binds to VEGFR-2 and exerts a pivotal role in vascular permeability and neo-angiogenesis (4). In 1993, a monoclonal antibody targeting and neutralizing VEGFA was identified to inhibit tumor growth in xenograft models, which opened translational avenues for targeting VEGF-VEGFR signaling (5). These therapeutic agents can be broadly categorized into those which target the VEGF ligand and those which inhibit the cell surface receptor (6). VEGFR-TKIs is a class of small molecules targeted therapies that can selectively inhibit the phosphorylation of tyrosine kinase receptors, thereby suppressing tumor angiogenesis.”*

**References**

2.	Dakowicz D, Zajkowska M, Mroczko B. Relationship between VEGF Family Members, Their Receptors and Cell Death in the Neoplastic Transformation of Colorectal Cancer. Int J Mol Sci. 2022;23(6).

3.	Ferrara N, Gerber HP, LeCouter J. The biology of VEGF and its receptors. Nat Med. 2003;9(6):669-76.

4.	Liu G, Chen T, Ding Z, Wang Y, Wei Y, Wei X. Inhibition of FGF-FGFR and VEGF-VEGFR signalling in cancer treatment. Cell Prolif. 2021;54(4):e13009.

5.	Kim KJ, Li B, Winer J, Armanini M, Gillett N, Phillips HS, et al. Inhibition of vascular endothelial growth factor-induced angiogenesis suppresses tumour growth in vivo. Nature. 1993;362(6423):841-4.

6.	Ferrara N. Vascular endothelial growth factor: basic science and clinical progress. Endocr Rev. 2004;25(4):581-611.

**4- “Meta-analysis was performed through two scientists (HBY and JQH) independently”. I do no prefer to cite the authors’ names here. It is much better to talk about it generally.**

Response: Thank you for the suggestion. We have removed the authors' names from the methodology section.

**5- It is not recommended to use abbreviations in heading sections (e.g QoL, AFP, ADRs, etc…)**

**Response:** Thank you for your comments. In the resubmitted manuscript, we have utilized full names in the heading sections.

**6- The conclusion part has to be improved. It should include deep insights.**

Response: We appreciate for pointing out our shortcomings. We have enriched the conclusions with deeper insights in lines 524-539. Please see the below revisions.

*“This investigation indicated that TMPs + VEGFR-TKIs co-treatment has enhanced efficacy and reduced risk of ADRs, for middle-advance PLC patients. Furthermore, only the “TMPs + sorafenib” combination improved the one-year survival rate in long-term prognosis. In addition, TMPs + lenvatinib co-treatment has better ORR than the combination of TMPs with other VEGFR-TKIs. When it comes to DCR, TMPs + sorafenib co-treatment was better. Moreover, it was indicated that TMPs can reduce AFP and need further exploration. Based on the meta-analysis, it was recommended that for middle-advanced PLC treatment, the following traditional medicines should be combined with VEGFR-TKIs: P. ginseng, T. robiniophila Murr., S. nigrum, R. glutinosa, A. sinensis, Citrus × aurantium, C. aromatica, P. persica, E. sinensis walker, S. barbata, S. s. mutilans, M. pentadactyla Linnaeus, and G. glabra, as they have a significant effect on ORR/DCR. Additionally, following combinations of traditional medicines with VEGFR-TKIs were also recommended: E. sinensis Walker + S. nigrum + R. glutinosa; C. aromatica + P. ternate + Citrus × aurantium; S. barbata + S. s. mutilans. However, further verification is required with well-designed and high-quality clinical trials adhering to CONSORT Extension for Chinese Herbal Medicine Formulas guidelines.”*

**7- All figures’ resolution have to be improved.**

Response: Thank you for the suggestion. We have repositioned the figures according to the recommended format of PLOS ONE, adjusting the resolution to 300 dpi to meet your requirements.

**Reviewer #3:**

**1- The rationale for this review and its aims should be more clear for the readers.**

Response: We appreciate for pointing out our shortcomings. In the Introduction section, we have reiterated the rationale and aims of this review in lines 97-105. Please see the below revisions.

*“Much literature indicated that VEGFR-TKIs have enhanced the efficacy and reduced adverse drug reactions in PLC patients treated with combined TMPs + VEGFR-TKIs therapy compared to VEGFR-TKIs alone. These findings suggest that compared to current therapeutic modalities, TMPs may serve as effective complementary or alternative treatments for PLC with more favorable risk-benefit profiles. However, because of the limited number of clinical trials investigating the co-treatment of TMPs + VEGFR-TKIs and their small sample sizes, the evidence for its potential use is less convincing. Therefore, our objective is to assess the therapeutic efficacy and safety of TMPs in combination with VEGFR-TKIs for middle-to-advanced PLC, aiming to provide substantial evidence.”*

**2- I wonder if the authors could provide more recent studies from 2023, as I can see there is no any study from 2023.**

**- Also, it is worth mentioning that there is a limited number of studies from 2022.**

Response: Thank you for the suggestion. Due to the extended submission cycle, our initial manuscript had an outdated literature search timeframe. Consequently, we have updated our search to include all relevant literature up to April 12, 2024. After removing duplicates, a total of 158 relevant articles published in 2023 were identified. However, only a few met our inclusion criteria. Through literature screening, three articles passed the abstract screening stage. During full-text screening, one article was excluded due to the use of non-fixed TMPs in the trial, and another was excluded as it was not an RCT. Ultimately, one additional article meeting the criteria was included. In total, our study encompasses 26 articles, including six from 2022 and one from 2023. The relatively low number of articles from 2023 may be attributed to ongoing trials whose results have not yet been published, as well as updates to treatment guidelines. Moving forward, we will continue to monitor developments in this field and update our study accordingly.

**3- The manuscript contains some errors and inaccuracies in terms of typography. I kindly request you to review it carefully and make the necessary revisions.**

Response: Thank you for pointing out this mistake. We have checked and revised the manuscript carefully according to PLOS ONE's “Submission Guidelines”.
