# Supplementary material for: Combinatorial treatment with traditional medicinal preparations and VEGFR-tyrosine kinase inhibitors for middle-advanced primary liver cancer: A systematic review and meta-analysis
Source: PLoS One. 2024 Nov 22;19(11):e0313443. doi: 10.1371/journal.pone.0313443 (PMC11584121; doi:10.1371/journal.pone.0313443)
Supplement: Attachment — Submitted filename: Response to Reviewers.docx [file pone.0313443.s007.docx]

Dear Editors and Reviewers:

Thank you for your letter and for the reviewers’ comments concerning our manuscript entitled “Combinatorial treatment with traditional medicinal preparations and VEGFR-Tyrosine Kinase Inhibitors for middle-advanced primary liver cancer: A systematic review and meta-analysis” [Manuscript ID: (PONE-D-23-16652)]. Those comments are all valuable and very helpful for revising and improving our paper, as well as the important guiding significance to our researches. We have studied comments carefully and have made correction. The main corrections in the manuscript and the responds to the reviewer’s comments are as below.

Please let us know if there is further issue need to be revised.

Kind regards,

Hong-Gang Zheng on behalf of the author team

**Response to Editors：**

**Response:** Thank you for your comments. We rechecked the references and confirmed that they are complete and accurate.

**Response to Reviewers：**

**Reviewer #4:**

**-Line 144, what the time point of the inception for the literature search?**

Response: We appreciate your pointing out our shortcomings. We have added the inception time information on lines 143-144. Please see the below revisions.

*“All randomized controlled trials, published in both Chinese and English, were searched from January 1, 2000 until April 12, 2024.”*

**-Line 177, what do you mean dichotomous datasets or continuous datasets? Do you mean that the outcomes are dichotomous1 or continuous variables?**

Response: Thank you for the comments. By "dichotomous datasets" and "continuous datasets," we were referring to dichotomous and continuous variables. We have revised the terminology in lines 176-179 to clarify this and eliminate any potential confusion for the readers. Please see the below revisions.

“*Using the risk ratio (RR), which represents the ratio of the studied outcomes between the co-treatment group and the mono treatment group, along with 95% confidence intervals (CI),* ***dichotomous variables*** *were identified. For* ***continuous variables****, the standardized mean difference (SMD) with 95% CI was employed.*”

**-Nowhere to indicate which results are from Tables 3-5 in the manuscript. It needs to cite Tables 3-5 where their results are described in the text.**

Response: Thank you for pointing out this mistake. In the revised manuscript, we referenced Table 3-4 on lines 284-285 and Table 5 on line 367.

*Lines 284-285 “Sub-cohort assessments for ORR and DCR were performed for the target drug regimen, treatment course, KPS score, and TMP form (Table 3 and Table 4).”*

*Line 367 “Table 5A and Table 5B present the sub-cohort analysis of ORR and DCR for these traditional medicines and their combinations.”*

**-To be easily understood, for all forest plots, please add “Favors” before each treatment group label, e.g “Favors TMP plus VEGFR-TKIs” and “Favors VEGFR-TKIs”.**

Response: Thank you for the comments. We have added "Favors" before each treatment group label in all the forest plots to ensure they are easier for readers to understand.

**-For** **Figures 4-7, the term “Risk Ratio” may be confusing for readers since some of these outcomes (ORR, DCR, or one-year OS) are the bigger the better. The bigger values of “Risk Ratio” don’t mean bad Risk. It’s better to use “Fold Change” to replace “Risk Ratio” and make a note to indicate that Flod Change** **represents the ratio of the studied outcome (ORR, DCR, one-year OS, and ADR) between the co-treatment group versus the mono treatment.**

Response: Thank you very much for your insightful comments. We appreciate your valuable suggestions. In response, we have added an explanation of the risk ratio on lines 183-184. Additionally, we have included a legend in Figure 4 where the RR value first appears to help clarify any potential confusion for the readers. Our study design adheres to the PRISMA guideline, which recommend using the risk ratio to calculate effect sizes. The GRADE criteria for assessing the quality of evidence also requires the use of RR values. After careful consideration and discussion within our team, we kindly request to continue using the term 'Risk ratio' to ensure accuracy and consistency throughout the manuscript. Please see the below revisions.

*Lines 183-184 “Using the risk ratio (RR), which represents the ratio of the studied outcomes between the co-treatment group and the mono treatment group,...”*

*“Fig 4. Forest-plot/pooled RRs comparing the influence of TMPs + VEGFR-TKIs and VEGFR-TKIs on ORR.* ***Risk ratio represents the ratio of the studied outcome between the co-treatment group and the mono treatment group.****”*

**-For Figure 8, please add the title to the** **horizontal Axis. Please add Egger’s test for Fig. 8 C and D.**

Response: Thank you for the comments. We have added the title to the horizontal axis, and we performed Egger’s test for the outcomes of hypertension and hand-foot skin reactions corresponding to Fig. 8 C and D in lines 407-409. Please see the revisions below.

*“Moreover, we additionally performed egger’s test on hypertension (p = 0.1206) and hand-foot skin reactions (p = 0.0701), and similarly found no significant publication bias in these outcomes.”*

*Revised Fig 8.*
