# Supplementary material for: Combinatorial treatment with traditional medicinal preparations and VEGFR-tyrosine kinase inhibitors for middle-advanced primary liver cancer: A systematic review and meta-analysis
Source: PLoS One. 2024 Nov 22;19(11):e0313443. doi: 10.1371/journal.pone.0313443 (PMC11584121; doi:10.1371/journal.pone.0313443)
Supplement: Attachment — Submitted filename: Response to Reviewers.docx [file pone.0313443.s008.docx]

Dear Editors and Reviewers:

Thank you for your letter and for the reviewers’ insightful comments regarding our manuscript, titled “Combinatorial Treatment with Traditional Medicinal Preparations and VEGFR-Tyrosine Kinase Inhibitors for Middle-Advanced Primary Liver Cancer: A Systematic Review and Meta-Analysis” (Manuscript ID: PONE-D-23-16652). We greatly appreciate the valuable and constructive feedback, which has been instrumental in revising and improving our manuscript and guiding our research.

We have carefully considered each comment and made the necessary revisions. Below, we provide a summary of the main changes made to the manuscript and detailed responses to the reviewers' comments.

Please feel free to reach out if further revisions are needed.Kind regards,

Hong-Gang Zheng, on behalf of the author team

**Response to Reviewer：**

**Reviewer #4:**

**-The significant level for publication bias is recommended p<0.1 for both Begg and egger [reference]. Please also report the intercepts estimated from Egger’s regressions.**

Response: We appreciate your insightful suggestion. In response, we have now reported the intercepts estimated from Egger’s regression on lines 384-389. Including these intercepts ensures a more rigorous assessment of potential publication bias in our analysis. Below is the relevant revision:

*“Following the results of the funnel plot and Begg’s test, we further conducted Egger’s tests for hypertension (p = 0.1405,* ***intercept = 0.0526****) and hand-foot skin reactions (p = 0.0952,* ***intercept = 0.0180****) to enhance the rigor of our assessment. A significance level of p < 0.1 is recommended for detecting publication bias in both Begg’s and Egger’s tests [reference]. After a comprehensive evaluation, we concluded that there is potential publication bias in the results for DCR, hypertension, and hand-foot skin reactions.”*

[reference]: Hayashino Y, Noguchi Y, Fukui T. Systematic evaluation and comparison of statistical tests for publication bias. J Epidemiol. 2005 Nov;15(6):235-43. doi: 10.2188/jea.15.235. PMID: 16276033; PMCID: PMC7904376.

We believe these changes enhance the robustness of our analysis, and we welcome any further comments you may have.

**-To be conservative, authors may add discussions about the potential publication bias among the studied outcomes.**

Response: Thank you for your insightful comment. We have added discussions on the potential publication bias across the studied outcomes, as suggested, on lines 515–525. Please see the revisions below:

*“Using funnel plots, Begg’s test, and Egger’s test, we identified potential publication bias in DCR, hypertension, and hand-foot skin reactions. This may be attributable to publication bias favoring positive results, wherein studies with significant findings are more likely to be published. Additionally, some of the included studies had small sample sizes, which, if they happened to demonstrate larger effects, may have been more readily published, even though these effects may not be broadly representative. This can lead to potential publication bias. Furthermore, the literature search was restricted to studies published in English and Chinese, which may have contributed to bias as well. We recommend that future research efforts include pre-registered studies and encourage both researchers and journals to publish studies with non-significant findings to mitigate the impact of publication bias.”*

**-Please revise the table column labels in fig 7, change “M-H, Fixed” to “M-H, Fixed/Random”, so it reflects some of the summarized results are based on random effect models.**

Response: Thank you for pointing out this mistake. we revised the labels in Fig7. Please see the revisions below:

*Revised Fig 7.*
