# Supplementary material for: Combinatorial treatment with traditional medicinal preparations and VEGFR-tyrosine kinase inhibitors for middle-advanced primary liver cancer: A systematic review and meta-analysis
Source: PLoS One. 2024 Nov 22;19(11):e0313443. doi: 10.1371/journal.pone.0313443 (PMC11584121; doi:10.1371/journal.pone.0313443)
Supplement: Attachment — Submitted filename: Response to Reviewers.docx [file pone.0313443.s009.docx]

Dear Editors and Reviewers:

Thank you for your letter and for the reviewers’ insightful comments regarding our manuscript, titled “Combinatorial Treatment with Traditional Medicinal Preparations and VEGFR-Tyrosine Kinase Inhibitors for Middle-Advanced Primary Liver Cancer: A Systematic Review and Meta-Analysis” (Manuscript ID: PONE-D-23-16652). We greatly appreciate the valuable and constructive feedback, which has been instrumental in revising and improving our manuscript and guiding our research.

We have carefully considered each comment and made the necessary revisions. Below, we provide a summary of the main changes made to the manuscript and detailed responses to the reviewers' comments.

Please feel free to reach out if further revisions are needed.Kind regards,

Hong-Gang Zheng, on behalf of the author team

**Response to Reviewer：**

**Reviewer #4:**

**-Lines 382-385, insignificant publication bias should not include either DCR (p = 0.0852) or hypertension (p = 0.0546) because a significance level of p < 0.1 is recommended for detecting publication bias in both Begg’s and Egger’s tests.**

Response: Thank you for pointing out this mistake. We agree that a significance level of p < 0.1 is recommended for detecting publication bias in Begg’s and Egger’s tests. We have revised the section accordingly. Please see the revisions below:

*Line 381-384*

*“These data were validated by Begg’s test, which indicated insignificant publication bias for ORR (p = 0.3323), hand-foot skin reactions (p = 0.3506), and gastrointestinal reactions (p = 0.9562). However, potential publication bias was indicated for DCR (p = 0.0852), and hypertension (p = 0.0546).”*
